# Unraveling heterogeneous susceptibility and the evolution of breast cancer using a systems biology approach

**DOI:** 10.1186/s13059-015-0599-z

**Published:** 2015-02-21

**Authors:** Andrés Castellanos-Martín, Sonia Castillo-Lluva, María del Mar Sáez-Freire, Adrián Blanco-Gómez, Lourdes Hontecillas-Prieto, Carmen Patino-Alonso, Purificación Galindo-Villardon, Luis Pérez del Villar, Carmen Martín-Seisdedos, María Isidoro-Garcia, María del Mar Abad-Hernández, Juan Jesús Cruz-Hernández, César Augusto Rodríguez-Sánchez, Rogelio González-Sarmiento, Diego Alonso-López, Javier De Las Rivas, Begoña García-Cenador, Javier García-Criado, Do Yup Lee, Benjamin Bowen, Wolfgang Reindl, Trent Northen, Jian-Hua Mao, Jesús Pérez-Losada

**Affiliations:** Instituto de Biología Molecular y Celular del Cáncer (CIC-IBMCC), Universidad de Salamanca/CSIC, Salamanca, 37007 Spain; Instituto de Investigación Biomédica de Salamanca (IBSAL), Salamanca, 37007 Spain; Departamento de Fisiología y Farmacología, Universidad de Salamanca, Salamanca, 37007 Spain; Departamento de Estadística, Universidad de Salamanca, Salamanca, 37007 Spain; Departamento de Parasitología CIETUS, Universidad de Salamanca, Salamanca, 37007 Spain; Servicio de Bioquímica Clínica, Hospital Universitario de Salamanca, Salamanca, 37007 Spain; Departamento de Medicina, Universidad de Salamanca, Salamanca, 37007 Spain; Servicio de Oncología, Hospital Universitario de Salamanca, Salamanca, 37007 Spain; Departamento de Anatomía Patológica, Facultad de Medicina Universidad de Salamanca, Salamanca, 37007 Spain; Unidad de Bioinformática, CIC-IBMCC, Salamanca, 37007 Spain; Departamento de Cirugía, Universidad de Salamanca, Salamanca, 37007 Spain; Advanced Fermentation Fusion Science and Technology, Kookmin University, Seoul, 136-702 Korea; Department of Bioenergy/GTL & Structural Biology, Life Sciences Division, Lawrence Berkeley National Laboratory, Berkeley, CA 94720 USA; Life Sciences Division, Lawrence Berkeley National Laboratory (LBNL), University of California, Berkeley, CA 94720 USA; Current address: Institute for Research in Biomedicine (IRB Barcelona), C/Baldiri Reixac 10, 08028 Barcelona, Spain

## Abstract

**Background:**

An essential question in cancer is why individuals with the same disease have different clinical outcomes. Progress toward a more personalized medicine in cancer patients requires taking into account the underlying heterogeneity at different molecular levels.

**Results:**

Here, we present a model in which there are complex interactions at different cellular and systemic levels that account for the heterogeneity of susceptibility to and evolution of ERBB2-positive breast cancers. Our model is based on our analyses of a cohort of mice that are characterized by heterogeneous susceptibility to ERBB2-positive breast cancers. Our analysis reveals that there are similarities between ERBB2 tumors in humans and those of backcross mice at clinical, genomic, expression, and signaling levels. We also show that mice that have tumors with intrinsically high levels of active AKT and ERK are more resistant to tumor metastasis. Our findings suggest for the first time that a site-specific phosphorylation at the serine 473 residue of AKT1 modifies the capacity for tumors to disseminate. Finally, we present two predictive models that can explain the heterogeneous behavior of the disease in the mouse population when we consider simultaneously certain genetic markers, liver cell signaling and serum biomarkers that are identified before the onset of the disease.

**Conclusions:**

Considering simultaneously tumor pathophenotypes and several molecular levels, we show the heterogeneous behavior of ERBB2-positive breast cancer in terms of disease progression. This and similar studies should help to better understand disease variability in patient populations.

**Electronic supplementary material:**

The online version of this article (doi:10.1186/s13059-015-0599-z) contains supplementary material, which is available to authorized users.

## Background

Worldwide, breast cancer is one of the most frequent tumors in women and indeed more than a million women are diagnosed with this disease every year [1]. An essential question underlying this circumstance is why patients who seem to have the same pathological condition, even in hereditary forms of breast cancer, progress differently and have different clinical outcomes [2-4]. Clinical manifestations reflect perturbations of complex intra- and intercellular networks that link molecular and cellular processes with tissue/organ subphenotypes and clinical semiology. Functionally interconnected layers comprising, among others, genotype, gene expression, cell signaling and metabolic pathways form these networks, leading to pathophysiological and clinical manifestations [5,6]. The genetic backgrounds of patients would influence such networks of interactions [7,8]. Identifying associations at different levels, and the influence of the genetic background in them, is crucial if we are to fully understand the different patterns of behavior of the disease, and will help to design better strategies for individualized cancer prevention and therapy. The identification of these complex networks in human populations is a difficult task owing to genetic heterogeneity and complex interactions with the environment [7,9,10]. However, crosses of inbred mouse strains with homogenous genomes and fairly uniform phenotypes offer a unique opportunity to tackle these questions under simpler conditions [7,11,12].

**Figure 1:**
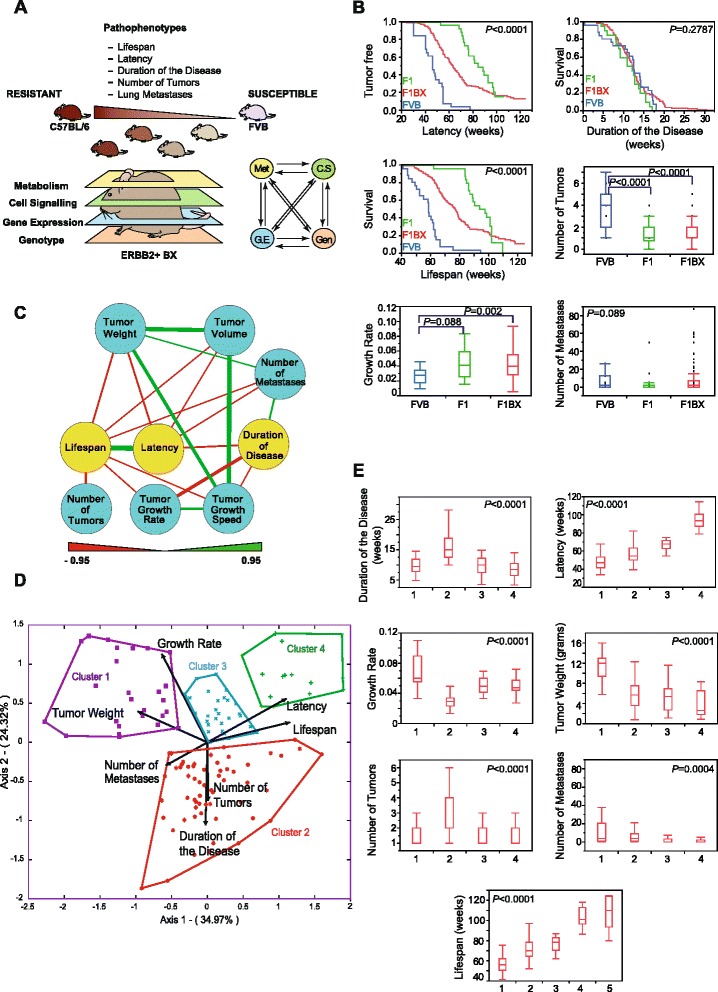
**Differentiation of ERBB2-induced breast cancer according to different traits. (A)** Experimental design. We generated a cohort of mice that were genetically heterogeneous and with different degrees of susceptibility and progression to breast cancer. Thus, we dissected the disease into different pathophenotypes. Then, we classified the mice according to prognosis. We considered different clinical pathophenotypes of ERBB2-positive tumors as the result of multiple molecular associations at different levels from a systems biology perspective to predict clinical outcomes. In sum, we show a global scenario of complex interactions at different levels that account for the heterogeneity in tumor behavior and susceptibility. **(B)** Comparison among different tumor pathophenotypes in FVB, F1 and F1BX mice. We show detailed comparisons of all the different pathophenotypes elsewhere (Table S1 in Additional file 1). **(C)** The network shows associations among different tumor pathophenotypes in the F1BX population (Cytoscape Software). Nodes represent breast cancer pathophenotypes: the yellow nodes are temporal stages of the disease and the blue nodes depict tumor traits. Edges indicate correlation coefficients, green for positive and red for negative correlations. Edge line width is directly proportional to the value of the *r* coefficient. We include all correlations with *P* < 0.05 in the figure. All *r* coefficient values are in Table S2 in Additional file 1. **(D)** Prognosis clusters identified by principal component analysis as shown by an HJ-biplot. A fifth cluster formed by the mice without tumors is not included in the figure. **(E)** Distribution of tumor pathophenotypes in each different cluster. The fifth cluster, which encompasses the mice that did not develop tumors, is not represented in the figure. The numerical values of this figure and *post hoc* tests are reported elsewhere (Table S3 in Additional file 1).

ERBB2/NEU/HER2 (henceforth ERBB2)-positive breast cancers constitute 20 to 30% of all mammary gland tumors. The amplification and overexpression of *ERBB2* is a marker of poor prognosis [13], but the progression of these tumors is heterogeneous [3]. Complex networks at different molecular levels influenced by the genetic background could account for this variable progression of breast cancer [5,6], and the consideration of molecular features from different profiling data types may help to predict therapeutic response [14]. To address this issue, we generated a genetically heterogeneous population of mice with different susceptibilities to breast cancer by a backcross between *MMTV-ErbB2* transgenic mice [15] in a FVB genetic background showing high tumor susceptibility and C57BL/6 resistant mice [16-18]. Here, for the first time we differentiate ERBB2-positive breast cancers according to different pathophenotypes and molecular subphenotypes to evaluate their associations by a systems biology approach (Figure 1A). We report a global scenario of complex interactions at cellular and systemic levels that accounts for the heterogeneity in ERBB2-positive breast cancer behavior and susceptibility. We integrated these different molecular levels to better define cancer prognosis. This global scenario enabled specific achievements: first, we demonstrate parallels between ERBB2-positive tumors from humans and backcross mice at the clinical, genomic, gene expression and signaling levels. We defined different tumor traits and mouse clusters of prognosis at those different levels. Second, the global architecture of signaling pathways was similar in breast tumors and livers (where the oncogene is not expressed), and these signaling pathways also defined both tumor pathophenotypes and mouse clusters of prognosis. For instance, mice with intrinsically higher levels of AKT and ERK pathways were less likely to develop tumors. Third, we recognized specific molecular features of the disease; for example, pAKT1(S473) levels modified metastatic capability. Thus, tumors that metastasized to the lung showed lower levels of pAKT1(S473). In addition, mice that developed tumors with a short latency expressed low levels of pERK_1/2_ due to a partial block in the phosphorylation at the RAF-MEK step. Fourth, we identified a pattern of serum metabolites collected at the disease-free stage that predicted different traits of tumor progression and defined mouse clusters associated with prognosis. Finally, we combined the different data types in Cox-regression models that predict cancer susceptibility and progression to identify the specific risk in single mice. The connections identified at different molecular and phenotypic levels with this approach should eventually permit a better understanding of heterogeneous breast cancer susceptibility and progression among patients and the development of more individualized clinical strategies.

## Results

### The genetic background modifies ERBB2-induced breast cancer susceptibility and progression

We studied the susceptibility and progression of breast cancer in *MMTV-ErbB2* transgenic mice from FVB, F1FVBxC57 (henceforth F1) and backcross (F1BX) genetic backgrounds. The FVB mice had a shorter tumor latency than F1 and F1BX. The tumor latency and lifespan in F1BX mice were between those of the FVB and F1 animals, 50% of them dying by 76.86 weeks of age (Figure 1B; Table S1 in Additional file 1). Furthermore, the FVB mice had a higher incidence, multiplicity, and average number (*P* < 0.0001) of breast tumors than their F1 and F1BX counterparts, but mice with a partial C57BL/6 genetic background showed a faster local tumor growth rate than the FVB animals, in agreement with previous work [17]. FVB mice had a higher incidence of metastases than the F1 and F1BX animals, but the number of metastases per mouse was very similar between the three groups (Table S1 in Additional file 1). These results suggest that the C57BL/6 genetic background carries genetic determinants that build up resistance to most of these breast tumor pathophenotypes in a dominant manner.

Next, we explored which breast tumor traits mainly influenced lifespan variability in the F1BX population. Tumor latency was found to be the most important (*P* < 0.0001); in addition, tumor numbers, local growth and number of metastases were negatively related to lifespan. The F1BX tumors that appeared in older mice (>70 weeks) rarely metastasized (*P* = 0.004) (Figure 1C; Table S2 in Additional file 1), which mirrors the patterns observed in human populations [19,20]. This tumor behavior regarding dissemination in ageing mice was not seen in the FVB or F1 mice (*P* = 0.788 and *P* = 0.975, respectively).

Based on a principal component and biplot analysis of their clinical characteristics, we grouped the F1BX mice in five prognostic clusters. Cluster 1 contained those mice with the shortest lifespans and latencies, the highest number of metastases, and the highest final tumor weight, whereas based on the same pathophenotypes cluster 4 comprised animals with the best prognosis. We also distinguished clusters 2 and 3, considered as medium-poor and medium-good prognoses, respectively. Cluster 5 included mice with no tumors after two years of experimentation (Figure 1D,E; Table S3 in Additional file 1). Intriguingly, the F1 mice with tumors where the disease was least aggressive were mainly located in clusters 3 and 4 (and also in cluster 5 when mice without tumors were considered) whereas FVB were grouped in clusters 1 and 2, with a poorer prognosis (Figure S1 in Additional file 2). In conclusion, we generated a genetically heterogeneous population of mice, with variable degrees of susceptibility to and progression of breast cancer. Based on the behavioral patterns of the different pathophenotypes, we were able to classify mice in different clusters of prognosis.

### Identification of genomic regions associated with heterogeneous susceptibility to and progression of breast cancer

**Figure 2:**
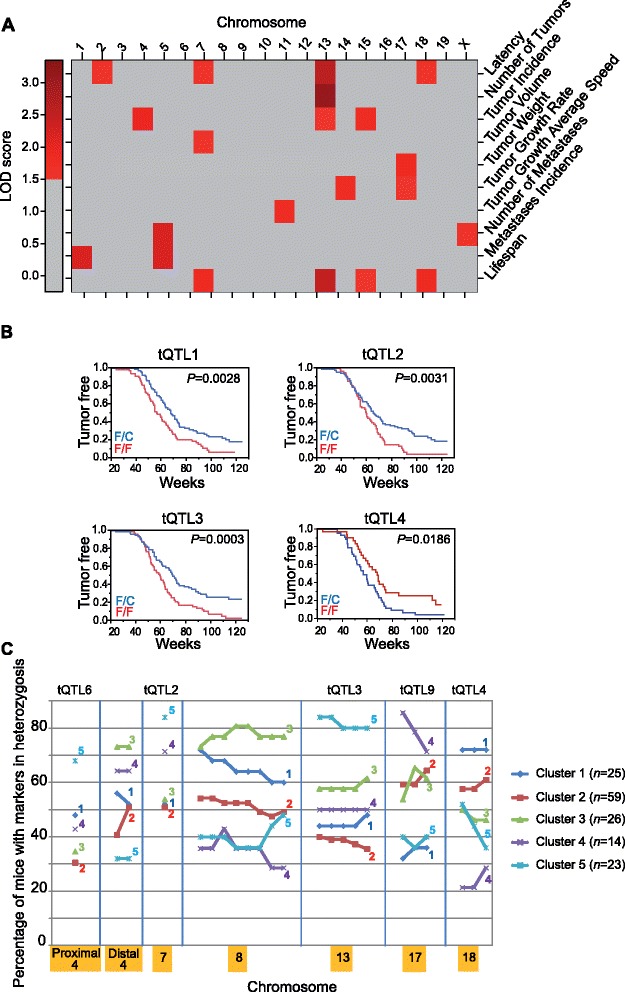
**Genomic regions associated with tumor behavior. (A)** Distribution of QTLs associated with different tumor pathophenotype (tQTL) variability across different mouse chromosomes. The color intensity reflects the LOD score level. LOD scores <1.5 are homogenously represented in gray. Detailed information concerning these results is reported in Table S4 in Additional file 1. **(B)** Effect on tumor latency of different loci identified by the Kaplan-Meier estimator. F/F indicates FVB homozygous and F/C indicates FVB/C57 heterozygous at those loci. **(C)** Distinction of all the clinical prognosis clusters defined at the genetic level. The figure shows the percentage of mice with a particular heterozygous genetic marker differentially present in the five clusters of mice based on prognosis. The genetic markers and percentages represented can be found in Table S5 in Additional file 1b.

Since the genetic background influences the clinical progression of ERBB2-positive breast cancer, we investigated the quantitative trait loci (QTLs) associated with heterogeneous tumor behavior in the F1BX mice by linkage analysis. Four loci, on chromosomes 2, 7, 13 and 18, were found to be associated with tumor latency; they were designated tQTL1 (tumor QTL1) to tQTL4, respectively (Figure 2A; Table S4 in Additional file 1). Mice homozygous for these markers (FVB/FVB) had a significantly shorter latency than heterozygous animals (FVB/C57), except for tQTL4 (Figure 2B; Table S4 in Additional file 1). A single locus, tQTL5, on chromosome 13 was associated with the number of tumors per animal. There were a number of loci associated with tumor incidence on chromosomes 4, 13 and 15. The locus on chromosome 13 seemed to be the same as the one previously identified for tumor latency (tQTL3). We also identified several QTLs related to different local tumor growth characteristics (including tumor volume, weight, average growth speed, and growth rate), and metastases. Four loci on chromosomes 7, 13, 15 and 18 were found to be associated with lifespan. As expected, these QTLs were also related to different tumor pathophenotypes (Figure 2A; Table S4 in Additional file 1).

We next identified genetic markers associated with the clinically defined clusters of prognosis. Significantly, cluster 5 included more mice heterozygous for tQTL2 (chromosome 7), tQTL3 (chromosome 13), and tQTL6 (chromosome 4). The good-prognosis cluster 4 had the highest percentage of mice heterozygous for tQTL9 (chromosome 17), whereas the poor-prognosis cluster 1 had the lowest proportion of heterozygosity for these genetic markers (Figure 2C; Table S5 in Additional file 1). These results showed that prognosis may also be determined genetically.

### Transcriptomic expression patterns from mouse tumors resemble human ERBB2-positive breast cancer

**Figure 3:**
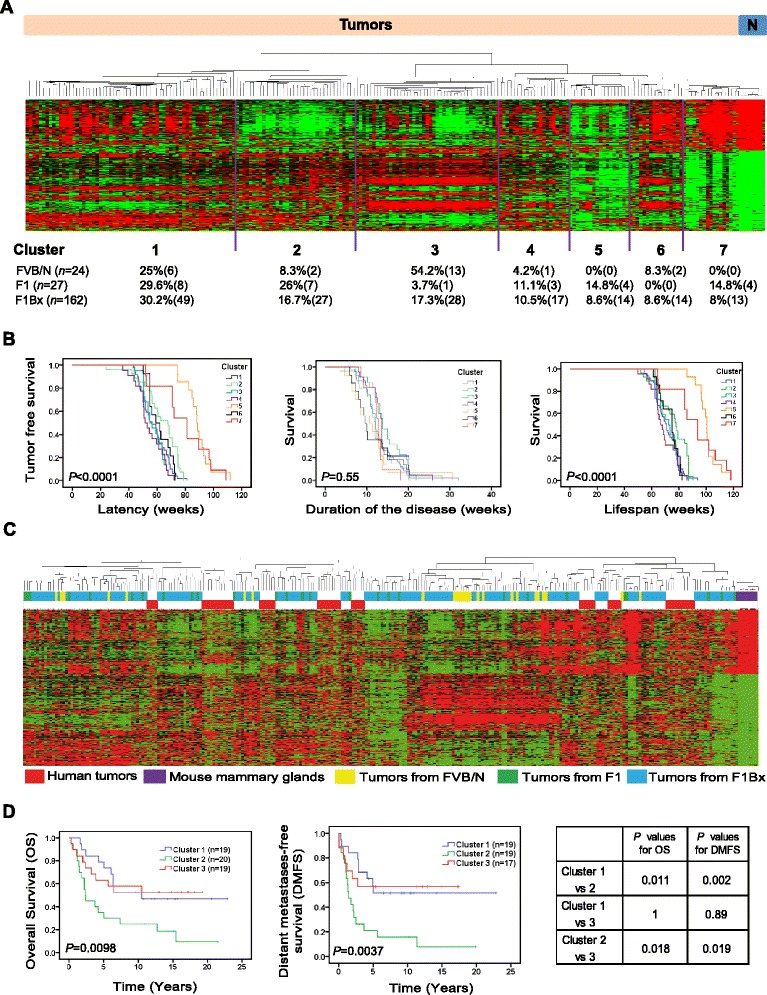
**Transcriptomic expression patterns from mouse and human tumors. (A)** Unsupervised clusters of mouse tumors based on a signature of 782 transcripts that defined seven mouse clusters derived from the mouse signature (MCMS; top). A list of transcripts included in this signature is provided in Table S6 in Additional file 1. The percentage and absolute number of tumors from FVB, F1 and F1BX mice included in each cluster are indicated at the bottom. ‘N’ means normal mammary glands. **(B)** The MCMS identified had prognosis implications in terms of latency and lifespan. The best prognosis MCMS clusters were 5 and 7. **(C)** Mouse and human tumors clustered together independently of the species of origin. The x-axis with the identification numbers is amplified in Figure S2 in Additional file 2. **(D)** Part of the mouse signature was able to classify human ERBB2 tumors in three clusters of prognosis. We include the list of 354 transcripts in Table S8 in Additional file 1 together with the heat map in Figure S3A in Additional file 2. The Kaplan-Meier curves show the different behavior of these clusters of human breast cancer regarding distant metastases, relapse and survival.

We then examined ERBB2-induced breast cancer in mice at the transcriptomic level. Using a fold-change cutoff of 2 and false discovery rate ≤0.005 between normal mammary tissues and tumors, together with a standard deviation of 0.7 relative to the expression values of that gene across all samples, based on the gap statistic [21] the mouse tumors were classified in seven unsupervised clusters with prognostic significance based on a 782-gene mouse signature (mouse clusters from mouse signature (MCMS); Figure 3A; Table S6 in Additional file 1). Expression clusters 5 and 7 encompassed mice with the best prognosis in terms of latency and lifespan (Figure 3B). More than 50% of the tumors of FVB origin were in expression cluster 3, and F1 tumors were mainly located in clusters 1 and 2, pointing to the influence of the genetic background (Figure 3A).

We have already observed some similarities between mouse and human tumors regarding disease evolution - that is, the tumors that appear earlier are more aggressive [19,20]. Interestingly, in the literature we discovered that most of the QTLs identified had homologous syntenic regions in humans related to breast cancer or other tumor types (Table S7 in Additional file 1 and supplementary references listed therein and shown in Additional file 3). Accordingly, we also compared the gene expression patterns of mouse tumors with human ERBB2 breast cancers available from Staaf *et al.* [3]. Interspecies unsupervised cluster analysis between mouse and human tumors revealed that the similarities between tumors from the same cluster were more marked than among tumors from the same species (Figure 3C). By measuring the distances in the dendrogram, we identified the samples closest to the human subgroups. The eight subgroups of human samples (red) were grouped with clusters of tumors formed mainly by backcross and F1 tumors (blue), but rarely of FVB origin (Figure S2 in Additional file 2). This indicates that tumors originating in mice with a heterogeneous background are more similar to human tumors than those originating in the FVB inbred strain. Moreover, 354 out of 782 transcripts were also found in the ERBB2 human transcriptional data [3] (Table S8 in Additional file 1). This 354-gene mouse signature allowed a classification of human tumors in three different clusters with significant differences in prognosis, in agreement with Staaf *et al.* [3] (Figure 3D; Figure S3A in Additional file 2), and, vice versa, the human signature also permitted mouse tumors to be classified in terms of prognosis (mouse clusters from human signature (MCHS)) (Figures S3B,C and S4A in Additional file 2). This suggests a similar pattern of gene expression between human and mouse ERBB2 tumors. Moreover, the distribution of human tumors among the three clusters identified with the mouse signature overlapped the clusters identified by Staaf *et al.* (*P* = 1.05 E^−8^; *R* = 0.667; Figure S3A in Additional file 2).

**Figure 4:**
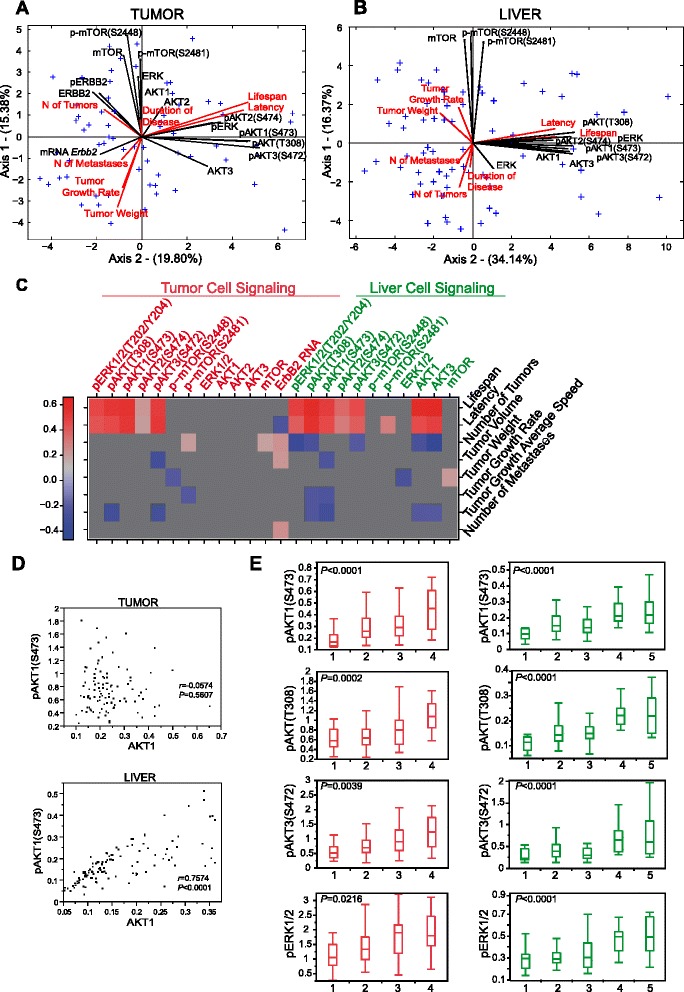
**Associations among signaling molecules from tumors and livers, and tumor pathophenotypes. (A,B)** Multivariate analysis revealed relationships among tumor pathophenotypes and representative molecules from signaling pathways obtained from tumors (A) and liver (B). We show analogous biplots representing only the signaling proteins from tumors and livers without pathophenotype variables elsewhere (Figure S5A,B in Additional file 2). **(C)** Schematic of the correlations between specific signaling molecules from tumors and livers, together with different tumor traits. All correlations with *P* < 0.05 were included. The correlation coefficient *r* is represented by a red-blue scale and for non-significant correlations is represented homogeneously in gray. We show the complete information from this figure in Table S9 in Additional file 1. **(D)** Correlation between pAKT1 and total AKT1 in liver and tumors. There was a strong correlation between total and phosphorylated levels of AKT1 in liver (*P* < 0.0001), but not in tumors. As indicated in the manuscript, this means that the pAKT/total AKT ratio in the liver is constant, but not in the tumors, indicating that the percentage of total AKT phosphorylated in the livers of these mice is always the same, while in the tumors it is random. **(E)** Distribution of some protein signaling molecules from tumors and livers across the different prognosis clusters. Note the similarities between these signaling molecules from tumors (red) and livers (green) regarding differential statistically significant levels among prognosis clusters. We include further, similar examples in Figure S5C in Additional file 2. The values represented in (D) are provided in Table S10 in Additional file 1.

We then wondered whether the prognostic clusters of mouse tumors previously defined (Figure 1D) were enriched in particular expression signatures. As expected, the clinical cluster 4, with the best prognosis (shown in Figure 1D), mainly overlapped the gene expression cluster 5, shown in Figure 3A,B, obtained with the mouse signature (MCMS 5) (Figure S4B in Additional file 2). The tumors from clinical clusters 1, 2 and 3 were not preferentially located in any cluster of gene expression. They were more or less randomly distributed, mainly among the expression clusters 1, 2, 3, 4 and 6 (Figure S4B in Additional file 2), which is in agreement with the overlapping curves of latency of these clusters shown in Figure 4B. This indicates the absence of significant differences among them.

It is important to highlight that the similarity of tumor behavior in the F1BX and F1 mice and human patients with regard to some aspects of clinical, genomic and gene expression levels suggests the importance of F1BX studies to extrapolate additional knowledge to the human population.

### Differences in specific signaling molecules downstream of ERBB2 are associated with heterogeneous susceptibility to and progression of breast cancer

ERBB2 is essential in initiating and driving breast cancer progression [22]. Thus, we wondered whether different levels of *ErbB2* expression in tumors might also contribute to variations in breast cancer pathophenotypes in mice. We found a weak negative correlation between *ErbB2* RNA levels and tumor latency, and positive associations with the number of tumors and metastases (Figure 4A,C; Table S9 in Additional file 1).

We then wondered whether different levels of some specific signaling molecules downstream of ERBB2 might influence susceptibility to and progression of breast cancer among mice. The global scenario of associations among some representative molecules of these pathways evaluated by principal component analysis revealed that they were associated to different extents with breast cancer pathophenotypes. For example, these phospho (p)-proteins, except p-mTOR, were positively correlated with latency and lifespan, but were negatively related to metastasis and tumor numbers (Figure 4A,C; Table S9 in Additional file 1). These signaling molecules from tumors were also able to distinguish among previously described clusters of prognosis. More distant clusters showed more molecular differences than those close to each other. For example, the poor prognosis cluster 1 was different from the good prognosis cluster 4 in having significantly lower levels of activated pAKT1(S473) (*P* = 0.0003) and pAKT(T308) (*P* < 0.0001), among others (Figure 4E; Table S10 in Additional file 1), and the poorest prognosis clusters 1 and 2 were those with the highest levels of *ErbB2* RNA (Table S10 in Additional file 1; Figure S5C,D in Additional file 2).

**Figure 5:**
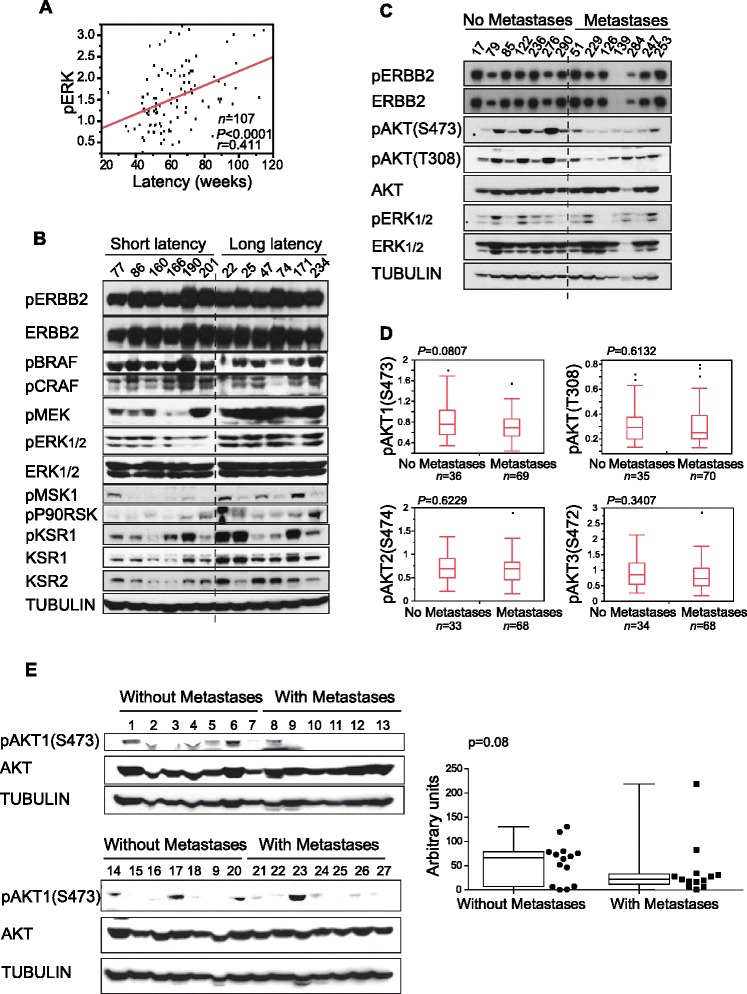
**Analysis of representative signaling molecules from pathways downstream of ERBB2 in the tumors. (A)** Positive correlation between levels of pERK_1/2_ (ELISA) and tumor latency. **(B)** Evaluation of upstream and downstream elements from the ERK_1/2_ pathway in tumors with short and long latencies. Tumors with a short latency show low levels of pERK_1/2_ and pMEK and lower levels of KSR_1/2_. **(C)** Evaluation of some elements of signaling pathways downstream of ERBB2 in F1BX tumors with and without lung metastases. Tumors that metastasize to the lung show low levels of pAKT(S473). We explored the AKT pathway to clarify this defect (Figure S6A in Additional file 2). This effect was not present in mice with a homogeneous genetic background (Figure S6B in Additional file 2). **(D)** The main pAKT isoform associated with dissemination to the lung in the F1BX population was pAKT1 (ELISA). **(E)** Breast tumors of human origin that disseminated show low levels of pAKT compared with those that did not metastasize.

In agreement with previous studies in patients and different types of cancer [23-25], tumor pERK_1/2_ levels were found to be positively associated with mouse lifespan and latency (*P* < 0.0001; Figures 4A,C and 5A; Table S9 in Additional file 1). Consistent with this defect in ERK_1/2_ activation in short latency tumors, the phosphorylation (and hence activation) of p-p90RSK(S380) and pMSK1(T581), known downstream mediators of ERK signaling, was impaired. Furthermore, the levels of pMEK were also low in short latency tumors, despite the normal activation of C- and B-RAF, indicating a partial blockage in the phosphorylation step between RAF and MEK. We evaluated the status of several scaffolding proteins, such as KSR1 and 2, which modulate RAF/MEK/ERK phosphorylation [26]. Breast tumors with a short latency and low levels of pERK_1/2_ and pMEK showed low levels of total KSR1 and 2, consistent with the reduced phosphorylation of MEK and ERK_1/2_ (Figure 5B).

High levels of pAKT(T308), pAKT1(S473) and pAKT3(S472) were associated with lifespan and tumor latency (*P* < 0.0001, for whichever pair was evaluated; Figure 4C; Table S9 in Additional file 1). Within a population of breast tumors with the same latency and similar levels of pERK_1/2_, there was a subpopulation that metastasized to the lung and showed low levels of pAKT(S473) (Figure 5C). Evaluation of different AKT isoforms in tumors from the backcross animals demonstrated that the isoform responsible for this difference was mainly pAKT1(S473) (Figure 5D), which highlights the importance of generating specific AKT isoform inhibitors. Unfortunately, we were unable to identify the mechanism responsible for this hypophosphorylation of AKT1(S473) (Figure S6A in Additional file 2). Furthermore, we failed to detect any changes in pAKT levels in tumors from the FVB and F1 mice with different numbers of lung metastases, showing that this effect only took place in the F1BX population (Figure S6B in Additional file 2). Breast tumors from patients that had metastases had discretely lower levels of pAKT1(S473), similar to tumors from the backcross mice (Figure 5E), which highlights the similarity between breast tumors from human and mice in this respect.

### Specific signaling molecules are controlled in a genetic background-dependent manner in breast tumors and livers

**Figure 6:**
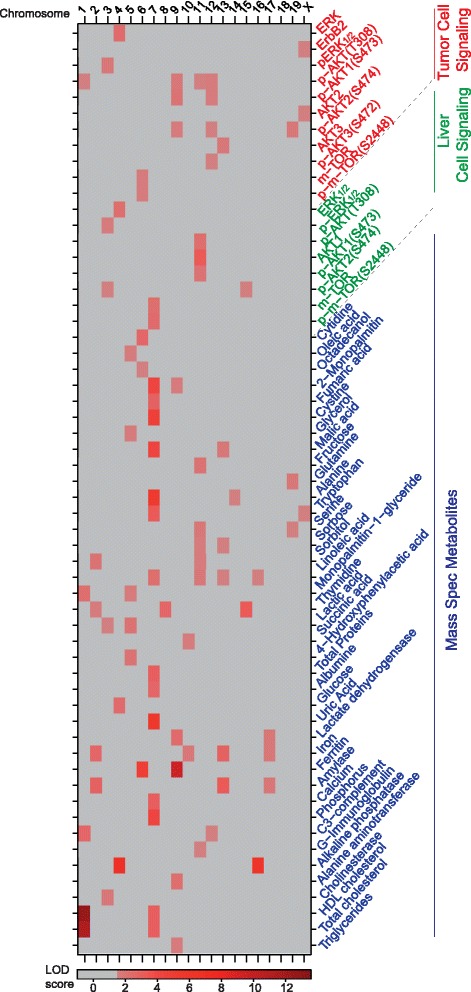
**The heat map summarizes QTLs associated with different subphenotypes, including signaling pathways from tumors and liver, and serum moieties.** LOD scores <1.5 are represented homogeneously in gray. The data depicted here can also be found in Tables S12, S14 and S21 in Additional file 1. HDL, high-density lipoprotein.

We evaluated the influence of the genetic background on the heterogeneous levels of some representative molecules specific to pathways downstream of ERBB2. We found differential protein levels between tumors from FVB and F1 mice, such as mTOR (*P* = 0.0014) or p-mTOR(S2448) (*P* = 0.030), among others (Table S11 in Additional file 1). In the F1BX mice we identified a number of tumor-signaling QTLs (tsQTLs) that could control the heterogeneity of some of those signaling molecules. Thus, for instance, a locus on chromosome 9 (tsQTL5) was simultaneously associated with the phosphorylation of the threonine 308 residue in AKT and serines 473 and 474 in AKT1 and AKT2, respectively (Figure 6; Table S12 in Additional file 1). The locus on chromosome 13 associated with total AKT3 levels (tsQTL11) shared the same marker peak as tQTL3, related to tumor latency, lifespan and tumor incidence, suggesting that the effect of tQTL3 could occur through the regulation of total AKT3 levels. Thus, despite the effect of the oncogene and the somatic progression of the tumors, we were able to detect the influence of the genetic background in the levels of these signaling molecules in breast cancer.

We then asked ourselves whether the influence of the genetic background in these signaling molecules was also present in other tissues, such as liver, spleen or kidney. The parental C57BL/6, FVB and F1 mice already showed differences in the levels of a number of some signaling molecules, such as pAKT1(S473) in kidney (*P* = 0.0019), pAKT(T308) in spleen (*P* = 0.0077) and pERK_1/2_ in liver (*P* = 0.027) (Table S13 in Additional file 1). Intriguingly, the FVB and F1 mice showed differences in signaling levels that were parallel in the different tissues; for instance, the F1 mice had higher levels of pAKT1(S473) than the FVB mice in liver, spleen and kidney. Moreover, the same differences were observed in tumors. Thus, the changes between F1 and FVB at these molecular signaling levels in tumors seemed to mirror what had taken place in other tissues. This suggests that the genetic background could influence these protein levels in the same way in several normal tissues and tumors (Figure S8 in Additional file 2). Accordingly, to further study the influence of the genetic background on protein levels in normal tissues, we evaluated the signaling molecules in livers from the whole F1BX population. We identified a number of possible liver signaling QTLs (LsQTLs; Figure 6; Table S14 in Additional file 1), but few of these overlap with tsQTLs, such as LsQTL3 and tsQTL6, indicating that the genetic control of these signaling molecules could in part be tissue-specific (Figure 6).

### Some signaling molecules in tumors and livers show similar patterns and are associated with breast cancer progression

Despite the observation of different QTLs related to signaling molecules in livers and breast tumors, the architectures of the associations among specific molecules in both tissues were similar (Figure 4A,B; Figure S5A,B in Additional file 2). For example, in both livers and tumors the levels of pERK_1/2_ and different pAKT isoforms were strongly correlated with each other in each tissue (*P* < 0.0001; Tables S15 and S16 in Additional file 1). In fact, although we failed to detect the ERBB2 protein in liver (Figure S5F in Additional file 2), we did note a number of obvious pair-wise correlations between certain signaling molecules from both tissues, particularly evident for pAKT(T308) (*P* < 0.0001), indicating that the levels of this moiety in tumors and livers would be very similar (Table S17 in Additional file 1). This can be seen in the biplot analysis in Figure S5A,B in Additional file 2. Indeed, the global correlations among certain specific signaling molecules in livers were analogous to those observed in tumors. The main difference was that livers showed a good association between the total levels of AKT isoforms and their phosphoprotein counterparts. Thus, there was a strong correlation between the total and phosphorylated levels of AKT1 in liver (*P* < 0.0001), but not in tumors. This indicates that the pAKT/total AKT ratio in liver was constant, whereas this was not the case in tumors. Accordingly, the percentage of total AKT phosphorylated in the livers of these animals is always the same, while in tumors it is random (Figure 4D).

Moreover, in both breast tumors and liver the same signaling molecules were related to variability in tumor pathophenotypes. These included pERK_1/2_ and different pAKT isoforms, both related to tumor latency and lifespan (*P* < 0.0001 for whichever pair was evaluated) (Figure 4C; Table S9 in Additional file 1). Principal component analysis revealed that the relationships among breast tumor pathophenotypes and the signaling molecules were also similar (Figures 4A,B). Moreover, the signaling molecules in the liver also defined the previously described prognostic clusters and allowed them to be discriminated (Figure 4E; Table S10 in Additional file 1; Figure S5C,E in Additional file 2).

### Serum metabolites measured before the onset of breast cancer are associated with prognosis

A close connection has been described between some of these signaling molecules (for example, AKT and mTOR) and metabolic processes [27,28]. Here, we observed an association between signaling proteins from liver and tumor traits and the influence of the genetic background in both of them. We therefore evaluated whether there were any metabolic differences determined by the genetic background among the F1BX mice that might help to predict the variability in tumor characteristics. To address this issue, we determined a number of metabolites in serum collected from the backcross mice at 3 months of age, before the first occurrence of tumors. We identified a number of serum metabolites that were correlated with the behavior of different breast tumor pathophenotypes. For example, the serum levels of N-acetyl-D-mannosamine were negatively associated with lifespan (*P* = 0.007) and latency (*P* = 0.009); cysteine was negatively associated with the duration of disease (*P* = 0.0002), and linoleic acid and sorbitol were negatively associated with the number of metastases, among others (Table S18 in Additional file 1; Figure S7A in Additional file 2).

We also evaluated a number of standard biomarkers routinely determined in clinical practice in humans. A number of these common markers were also found to be significantly associated with the behavior of different breast tumor pathophenotypes. For example, lifespan was positively related to body weight (*P* = 0.0016), total proteins (*P* = 0.018) and glucose, whereas it was negatively associated with amylase levels (*P* = 0.02). Some markers, such as calcium, magnesium, and C3-complement, were associated with tumor numbers (Table S19 in Additional file 1; Figure S7A in Additional file 2).

**Figure 7:**
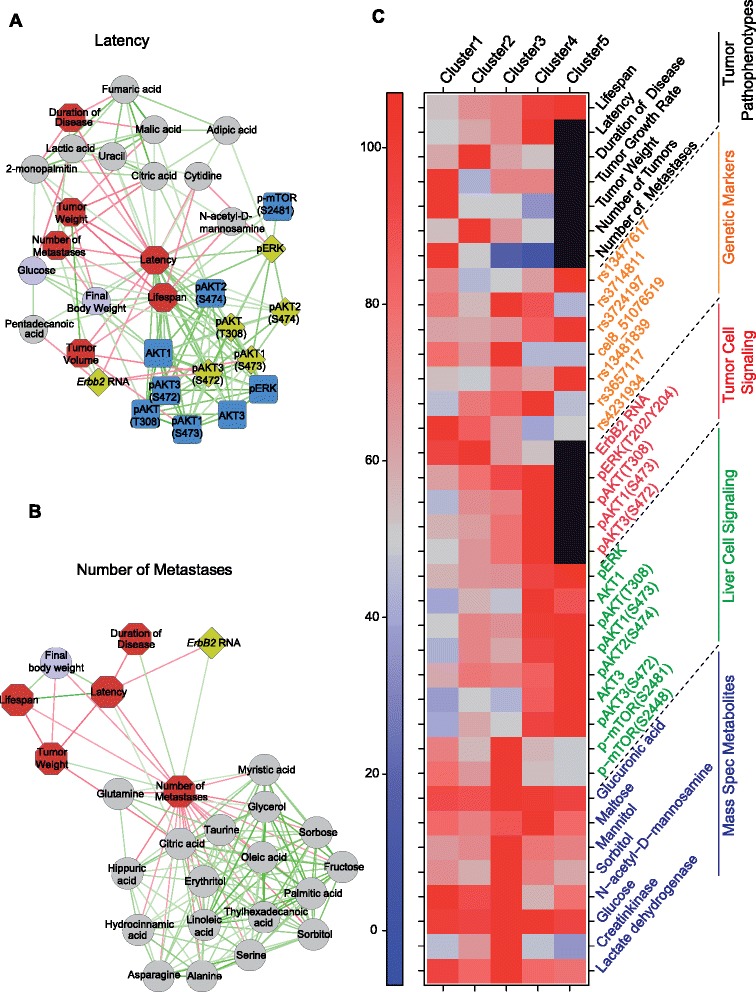
**Global scenario of subphenotypes associated with different tumor pathophenotypes and clusters of prognosis. (A,B)** Networks of subphenotypes associated with latency **(A)** and number of metastases (B). Red edges indicate a negative correlation, green edges a positive correlation. All simple correlations with *P* < 0.05 and *r* > 0.3 were included (Cytoscape Software). Other examples are shown in Figure S7B in Additional file 2. **(C)** This figure summarizes the distribution of tumor pathophenotypes and different levels of subphenotypes through the clusters of prognosis. The values of the data integrated here can be found in Tables S3, S5, S10 and S20 in Additional file 1. In the case of tumor pathophenotypes, signaling pathways and metabolites, the median is represented as a percentage of the highest value in each row. For genetic markers the percentage of mice that are heterozygotic in each cluster is shown. Non-applicable traits are represented in black.

In addition, we also report the global scenario of associations among the serum markers and other subphenotypes connected to each tumor pathophenotype (Figure 7A,B; Figure S7B in Additional file 2). Some of these potential serum biomarkers also defined the prognostic clusters described above. For example, maltose levels could differentiate between clusters 2 and 4 (*P* = 0.016), and mannitol between clusters 2 and 3 (*P* = 0.033), among others (Figure 7C; Table S20 in Additional file 1). Thus, the genetic background influenced the serum concentrations of some of these serum moieties associated with tumor behavior and we identified a number of metabolic QTLs (mQTLs) associated with them (Figure 6; Table S21 in Additional file 1).

**Figure 8:**
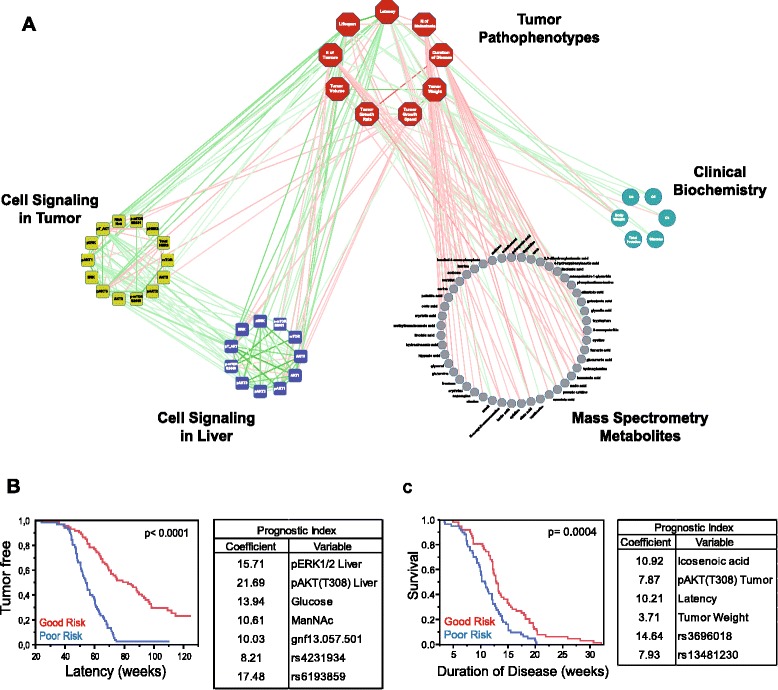
**Global scenario of multiple associations at different molecular levels. (A)** Network representation with tumor pathophenotypes (red octagons), tumor and liver cell signaling (yellow and blue squares, respectively), serum markers from mass spectrometry (gray circles) and clinical biochemical markers (green circles). Red edges indicate a negative correlation, green edges a positive correlation. All simple correlations with *P* < 0.05 and *r* > 0.3 were included. The Cystoscape document with all this information is included as Additional file 4. It should be opened with Cystoscape version 3.1.0. Readers can zoom in on and visualize all specific associations with the freely available Cytoscape software (see Material and methods section). **(B,C)** We constructed prognostic indices with the variables that predicted tumor latency **(B)** and duration of disease or survival with tumor **(C)**. In both models, mice were ranked according to their risk score and divided into two groups (good and poor risk) using the median risk score as cutoff. We show additional information in Table S22 in Additional file 1. The genetic markers used in these prognostic indices were gnf13.057.501 in tQTL 3, rs4231934 in tQTL 4, rs6193859 in tQTL 1, rs3696018 in tQTL 8, and rs13481230 in tQTL 11. ManNAc, N-acetyl-D-mannosamine.

Finally, we integrated the interactions among molecular elements from these different levels associated with the variability in disease susceptibility and progression in a single scenario (Figure 8A; Additional file 4). To better define the variability of the disease regarding tumor latency and disease duration, we implemented two Cox regression models and two corresponding prognostic indices, including genetic markers, tumor traits and signaling and metabolic levels as risk-predictor variables (Figure 8B,C; Table S22 in Additional file 1). All these results show that the heterogeneity of disease behavior among mice can be better explained by taking several levels of variability into account simultaneously.

## Discussion

Breast cancer induced by ERBB2 exhibits a broad and heterogeneous range of clinical progression in different patients [3]. Explaining why individuals who seem to suffer from the same histopathological disease show a different clinical progression is one of the main problems to be understood. Here, we tackled this question with a systems biology approach in a heterogeneous population of mice that developed ERBB2-positive mammary cancer with varied susceptibility and progression. We report a global scenario of complex interactions at cellular and systemic levels that accounts for the heterogeneity in ERBB2-breast cancer behavior and susceptibility. We integrated these different molecular levels to better define cancer prognosis. Thus, we identified multiple associations between specific breast cancer pathophenotypes at different molecular levels. We defined the specific prognosis of each individual mouse based on different pathophenotypes and, based on this, we classified each one in precise clusters of prognosis. These clusters were identified by principal component analysis and demarcated considering the progression of different pathophenotypes together. We then identified the associations at the genomic, transcriptomic, molecular signaling and metabolic levels associated with the progression of both each individual pathophenotype and each prognostic cluster. Thus, we individualized the prognosis of each different mouse regarding the progression of breast cancer, which is the main goal of personalized medicine.

Importantly, the model used here showed some similarity to the behavior of the disease in the human population, such as the development of less aggressive tumors in older mice, the fact that human and mouse ERBB2 tumors partially share gene expression signatures and that, at the protein level, mouse and human mammary tumors that did not disseminate along their progression show high levels of pAKT1(S473).

We noted that the architecture of associations among pERK_1/2_ and pAKT was similar in mammary tumors and livers from the backcrossed mice, and in both tissues pERK_1/2_ and pAKT showed similar associations with different disease pathophenotypes. Although the expression of the mouse mammary tumor virus (MMTV) promoter in different mouse models is not restricted to mammary tissue [15,29], we ruled out the presence of the ERBB2 protein in the liver. This indicates that ERBB2 oncogenic activity in tumors would not modify the global associations among some of these signaling molecules very much. However, there were also important differences; whereas the phosphorylated forms of ERK and AKT showed similar behavior, in terms of statistical associations, in tumors and livers, the case for total levels of ERK and AKT was completely different. Thus, whereas in livers there was a perfect correlation between total and phosphorylated forms, in the tumors there was no correlation at all, and hence they formed an approximately 90° angle in the biplot structure. This was particularly evident for the AKT1 isoform (Figure 4D). We do not know the reason for this different behavior of the AKT1 molecule between tumors and livers. It could be due to ERBB2 overexpression in the tumors, which might interfere with AKT1 phosphorylation. Overexpressed ERBB2 in the tumors could in turn increase the levels of pAKT1 in a non-regulated manner - that is, random - whereas in the livers the levels of pAKT1 would still be tightly regulated and constant without interference from ERBB2 overexpression.

Levels of activated pAKT and pERK_1/2_ in breast tumors would be determined by two main mechanisms: signaling from the ERBB2 oncoprotein itself, and signaling from other receptors and pathways normally present in the cell. These other receptors could influence the absence of positive correlations between ERBB2 and the phospho-proteins in tumor cells. Moreover, the existence of a negative correlation between ERBB2 and some of these phospho-proteins suggests that the pattern of correlation among them may be determined previously by a selection pressure in favor of cells with lower levels of ERBB2 expression and higher levels of phospho-proteins induced by other receptors. These increased levels of pAKT and pERK_1/2_ would be present not only in tumors but also in liver, and perhaps other tissues, due to a common general regulation. Thus, the expression levels of some molecules from different signaling pathways were similar in tumors and organs from F1 and FVB mice (Figure S8 in Additional file 2). In agreement with this possibility is the fact that even when the oncoprotein was not detected, as in liver, the same associations between pAKT and pERK_1/2_ and different breast cancer pathophenotypes were still present in the backcross population. As expected, the mice with a good prognosis were characterized by low levels of *ErbB2* in breast tumors but, surprisingly, also with high levels of pERK_1/2_ and pAKT in both tumors and liver. The fact that pERK_1/2_ and pAKT were increased in both breast tumors and livers from mice with long-latency disease would indicate that mice with naturally higher levels of pAKT and pERK_1/2_ would be more resistant to developing breast tumors induced by ERBB2.

It has been described that the expression of ERK_1/2_ in human breast cancer is heterogeneous and the implications of this for prognosis are controversial [23,30]. Our data indicate that high tumor levels of ERK_1/2_ activation were positively correlated with lifespan. Different studies have implicated high levels of pERK_1/2_ in good prognosis in breast cancer and other types of tumor [23-25]. In our study, the levels of pERK_1/2_ in tumors and liver were positively correlated with tumor latency and less aggressive tumors. In agreement with our findings, it has been suggested that ERBB2-positive tumors that are pERK_1/2_-positive tend to appear in older patients, while pERK_1/2_-negative tumors would predominate in younger individuals [31]. Additionally, high pERK_1/2_ levels have been linked to a significantly higher relapse-free survival rate [23,32]. Regarding metastases, different groups have reported that AKT1 down-regulation by short hairpin RNA in human breast cancer cell lines or its complete elimination by crossing *MMTV-ErbB2* mice with *Akt1* knockout animals leads to a higher rate of dissemination [33,34]. Here, we show that a site-specific phosphorylation defect at serine 473 of AKT1, with normal levels of total AKT1, is associated with higher metastatic capability, and this could help to explain the heterogeneity of this pathophenotype in the backcross population. Interestingly, breast tumors from patients that suffered metastases showed similar behavior in this aspect (Figure 5E).

We detected differences between specific mouse strains in the levels of some components of the AKT/mTOR pathway in liver tissue that are of great importance in metabolism [27,28]. We also identified serum biomarkers before the onset of the disease, associated with the behavior of tumors in mice. In the literature, some of them have already been reported to be modified in the serum of patients with different tumor types (for example, different fatty acids, taurine, inositol, and so on), but our results suggest that these metabolites would not only be useful as tumor progression biomarkers [35,36], but might also be used as predictors in disease-free individuals. We also found associations with a number of metabolites not previously identified as biomarkers, but at some level directly or indirectly related to breast or other types of cancer (Table S18 in Additional file 1 and supplementary references therein); however, more studies are needed to confirm their usefulness as tumor biomarkers.

We observed that ERBB2-induced breast cancer heterogeneity was associated with different molecular subphenotypes at the level of both the tumor pathophenotypes and the prognosis clusters (Figure 7; Figure S7B in Additional file 2). We also identified tQTLs related to the clinical progression of the disease and others associated with different subphenotypes (tsQTLs, LsQTLs and mQTLs), but we only detected a few overlapping QTLs associated with tumor traits and subphenotypes (Table S23 in Additional file 1). This loss of overlapping QTLs between subphenotypes that correlate could be associated with the ‘missing heritability’ described in the susceptibility of complex diseases, which remains a matter of controversy [37,38]. tQTLs associated with tumor progression are detected only if they exceed a particular threshold of linkage [39,40]. These tQTLs influence the heterogeneous behavior of known or unknown subphenotypes, which in turn determine the progression of tumor pathophenotypes. Additionally, the progression of each tumor pathophenotype is influenced by a number of subphenotypes at different levels (Figure 8A; Figure S9 in Additional file 2), each also modified by several QTLs. It is possible that most such subphenotypic QTLs might not be detected as tQTLs because their effect at the tumor level would be very small, and hence it would not be possible to detect their individual influence (Figure S9 in Additional file 2). Thus, it would not be feasible to capture the entire genetic variability of breast cancer (the same would be true for any other complex diseases) directly at the DNA level. This is in agreement with the fact that the multivariate regression models proposed, considering different tumor traits and molecular levels simultaneously, explain the heterogeneous susceptibility and progression of the disease better (Figures 8B,C; Table S22 in Additional file 1; Figure S9 in Additional file 2). The identification of QTLs that control different subphenotypes at different molecular and pathophysiological levels related to the disease could provide a way to identify part of the ‘missing heritability’. Additionally, the associations among all these subphenotypes establish a network of intra- and inter-level interactions leading to a systems biology structure [5,6], whose interpretation should permit a better understanding of disease variability among patients and allow more personalized medical care.

## Conclusions

Progress toward more personalized medicine in cancer patients requires new strategies to analyze the underlying factors determining their heterogeneous clinical evolution. We considered different clinical manifestations of ERBB2-positive mammary cancer as the result of multiple associations at different molecular levels from a systems biology perspective. We integrated genetic, transcriptomic, cell signaling and metabolic profiles to predict clinical outcomes in a population of mice with heterogeneous susceptibility to breast cancer. With this approach we modeled the heterogeneous behavior of the disease among the mice, considering not only the genetic level, but also several molecular layers and tumor traits simultaneously. The generation in human patients of similar structural networks formed by associations among different molecular subphenotypes related to different susceptibility and progression to breast cancer could reflect the global prognosis of the disease better than genetic markers alone or any other individual marker. In addition, the generation of these networks in heterogeneous populations of mice of controlled genetic and phenotypic variability could help identify the underlying molecular components that should contribute to these networks in human studies. For example, it would be interesting to evaluate the association between the levels of signaling molecules such as AKT and ERK in normal tissues and breast cancer susceptibility. The interpretation in human populations of these systems biology interactions should permit a better understanding of disease variability among patients and help towards a more personalized mode of medical care.

## Materials and methods

### Animals

All mice were housed in the Animal Research Facility of the University of Salamanca. All practices were accepted by the Institutional Animal Care and Bioethical Committee. FVB mice carrying the *ErbB2* protooncogene under the control of the mouse mammary tumor virus (MMTV) 3′promoter [15] were obtained from Jackson laboratories, and wild-type FVB/N and C57BL/6 mice were purchased from Charles River. Female mice were weaned at 3 to 4 weeks of age and analyzed for the inheritance of the *ErbB2* transgene. F1BX mice were generated by mating C57BL/6 males with *MMTV-ErbB2* transgenic females from a FVB genetic background; the transgene-positive F1 males generated were mated with FVB non-transgenic females. We generated 147 *ErbB2* F1BX mice. All mice were maintained in ventilated filter cages under specific-pathogen-free conditions and were fed *ad libitum*.

### Patient samples

Human primary breast tumors were collected at the University Hospital of Salamanca, Salamanca, Spain. The collection and the use of patient samples were approved by the institutional ethics review board of the University Hospital of Salamanca. Written informed consent for research using these tumor samples was obtained from all patients. Fresh human tumor tissue samples were obtained at the time of surgical resection of patient tumors. The samples were immediately snap-frozen in liquid nitrogen and then stored at −80°C in a freezer before use. Hematoxylin and eosin stained slides of frozen human tumor tissues were examined by the pathologists involved in the study to ensure that the tumor tissues selected had high-density cancer foci (>80%).

### PCR screening

PCR of the MMTV 3′ long terminal repeat promoter was used to detect the *ErbB2* transgene in positive mice, based on the method previously described by Gendler and colleagues [17]. The presence of the *ErbB2* transgene was detected by PCR on tail DNA. PCR was performed in a total volume of 25 μl in 200 μl PCR tubes or 96-well plates, with the following reagents: 2.5 μl of 10× PCR buffer with 1 mM MgCl_2_ (Takara, Otsu, Shiga, Japan) 200 μM dNTPs (Takara ), 5 μM 5′-CAGGTGCAAGCACTATTGACC-3′ and 5 μM of 5′-CTCAGAGCTCAGATCAGAACC-3′, 10 units of Taq polymerase (Takara), and ddH2O, 1 μl of DNA (approximately 200 ng). The PCR amplification program consisted of one cycle of 5 minutes at 94°C and 35 cycles of 30 s each at 94°C, 58°C and 72°C. The PCR products were analyzed in 1% agarose gels. Amplification of *ErbB2-*positive DNA resulted in a 559 bp fragment, as described elsewhere [17].

### Tumor pathophenotypes

Female transgene-positive mice were observed and palpated once a week for the manifestation of primary mammary tumors. We differentiated temporal stages of progression and tumor progression traits in ERBB2-positive disease. Within the first group, we distinguished: (i) tumor latency, defined as the period of time between the date of birth and the age when the first mammary tumor was palpated, approximately when its size was around 3 mm in diameter; (ii) duration of the disease, defined as the period of time between the appearance of the first tumor and the time of death; and (iii) lifespan.

Regarding tumor progression traits, we differentiated: (i) the number of tumors, determined by counting all visible tumors at necropsy. We distinguished between absolute tumor numbers, incidence and multiplicity. We used the absolute tumor number to designate the limit values within each tumor distribution spanned in each mouse group. Tumor incidence was defined as the proportion of ErbB2 female mice that generated at least one mammary tumor during the experiment, and tumor multiplicity was considered as the percentage of female mice with tumors that developed two or more lesions after a given period of time. (ii) Local tumor progression parameters: to determine these, once the tumor had appeared its location was recorded, the mice were observed, and the tumor volume was calculated every week. Tumor growth was determined with digital calipers, and tumor volume was estimated each week using the formula: Tumor volume = Length × Width^2^ × 0.5. We obtained the tumor growth rate after transforming the data logarithmically, and estimated a linear regression curve for each tumor. Then, we evaluated the means of the slopes of these lines for each genetic background [17]. Average growth speed was obtained using the expression (Final volume - Initial volume)/Duration of disease (weeks). We also considered final tumor weight and volume at the time of necropsy. (iii) Distant tumor progression: the incidence and multiplicity of lung metastases were quantified. The mouse MMTV-ErbB2 breast cancer model only disseminates to the lung [15]. Here, we also considered the absolute number, incidence and multiplicity of metastases. We defined the incidence of metastases as the proportion of ErbB2 female mice that had at least one mammary tumor metastasis in the lung at the time of necropsy; the absolute numbers of metastases were the extreme values between each metastasis distribution range within each genotype, and metastasis multiplicity was the proportion of female mice with metastases that developed more than one (two or more) lung metastases during the experiment. Mice were euthanized when they showed signs of sickness, when a rapidly growing tumor had developed, or wounds were observed. All animals were necropsied; their tumors were removed and fixed in 4% formaldehyde for 24 h, and then fixed in 70% ethanol, embedded in paraffin, and stained with hematoxylin and eosin for microscope examination to evaluate the pathology.

### SNP genotyping

Tail DNA concentrations were measured with a Nanodrop ND-1000 Spectrophotometer and PicoGreen double-stranded quantification (Molecular Probes, Thermo Fisher Scientific Inc., Waltham, MA USA). and were used for genotyping. The genome-wide scan was carried out at the Spanish National Center of Genotyping (CeGEN) at the Centro Nacional de Investigaciones Oncológicas (CNIO, Madrid, Spain). Illumina’s Mouse Low Density Linkage Panel Assay was used to genotype 147 F1BX mice at 377 SNPs. Genotypes were classified as FVB/FVB or FVB/C57BL/6. Ultimately, 250 SNPs were informative among the FVB and C57BL/6 mice; the average genomic distance between these SNPs was 9.9 Mb. The genotype proportion among the F1BX mice showed a normal distribution.

### Gene expression profiling and analysis

The quality and quantity of total RNA were determined using an Agilent 2100 Bioanalyzer and a NanoDrop ND-1000. Affymetrix mouse GeneChip mouse gene 1.0 ST arrays were used according to the manufacturer’s protocol. The data were initially normalized by robust multiarray average (RMA) normalization algorithms in expression console software (Affymetrix). The significance of analysis of microarray (SAM) used a two-class analysis with 100 permutations per comparison of the reference class to the target class, followed by a fold change cutoff of 2 and false discovery rate ≤0.005. The genes were further filtered by a standard deviation of 0.7 relative to the expression values of that gene across all samples. Gene clustering was accomplished by an uncentered correlation, and array clustering was carried out with the Spearman rank correlation using Gene Cluster v3.0 software and visualized using Java TreeView v1.1.6 software. The number of clusters was chosen based on the gap statistic for estimating the number of clusters together with the expression pattern [21]. Human orthologs of murine genes present on the human array platforms were used to cluster human microarray data, using gene clustering as above. To combine the human and mouse datasets, we first identified well-annotated mouse and human orthologous genes, after which we identified the systematic difference present between the two datasets and made a global correction to compensate for global biases using Z-score transformation. Then, we carried out unsupervised clustering analysis. Gene expression data for human ERBB2-positive breast cancers were obtained from Staaf *et al*. [3]. Their data are available through Gene Expression Omnibus, accession number GSE18328. The gene expression data from mouse ERBB2 tumors are also available through the same database with accession number GSE54582 [41].

### Protein analyses

Tumor, liver, kidney and spleen tissues were collected at necropsy, snap-frozen in liquid nitrogen and kept at −80°C. Proteins were extracted from frozen tissues. Ceramic beads, Precellys Lysing Kit CkMix, (Precellys, Bertin Technologies, catalog number 03961-1-009, Montigny le Bretonneux, France) were added to the tissues (10 to 50 mg) and these were homogenized for 10 s, 5.5 m/s (twice), using FastPrep Homogenizer (Thermo Savant, Thermo Fisher Scientific Inc., Waltham, MA USA) in RIPA buffer (150 mM NaCl, 1% (v/v) NP40, 50 mM Tris–HCl at pH 8.0, 0.1% (v/v) SDS, 1 mM EDTA, 0.5% (w/v) deoxycholate) containing protease and phosphatase inhibitor cocktails (Roche,Basel, Switzerland ) for tumors, or in 2× Cell Lysis Buffer containing protease inhibitors (Cell Signaling, catalog number 9803, Danvers, MA, USA) and 2 mM phenylmethylsulfonyl fluoride (PMSF) for liver, kidney and spleen tissues. Samples were incubated for 20 minutes on ice and protein extracts were passed through QIAshredder homogenizer columns (Qiagen, catalog number 79656, Hilden, Germany) to break down DNA. Supernatants were collected and quantified using the BCA Protein Assay Kit (Thermo Fisher Scientific Inc., catalog number 23228, Waltham, MA USA) and Albumin Standard (Thermo Fisher Scientific Inc.,, catalog number 23209, Waltham, MA USA). Equivalent amounts of proteins were resolved by SDS-PAGE and transferred to polyvinylidene difluoride (Immobilon-P, or Immobilon-FL (Millipore, Darmstadt, Germany) membranes for fluorescent secondary antibodies. Immunoblotting was performed using the following primary antibodies: anti-ERK1/2 (catalog number 9102), anti-phospho-ERK1/2 (Thr202/Tyr204) (catalog number 4370), anti-phospho-MEK1/2 (Ser227/221) (catalog number 9154), anti-phospho-c-RAF (Ser338) (catalog number 9427), anti-phospho-B-RAF (Ser445) (catalog number 2696), anti-B-RAF (L12G7) (catalog number 9434), anti-phospho-AKT (Ser473; D9E) (catalog number 4060), anti-AKT (11E7) (catalog number 4685), anti-phospho-AKT (Thr308) (catalog number 4056), anti-AKT1 (2H10) (catalog number 2967S), anti-phospho-KSR1 (Ser392) (catalog number 4951S), anti-KSR1 (catalog number 4640), anti-phospho-p90RSK (Ser380) (catalog number 9335), anti-phospho-MSK1 (Thr581) (catalog number 9595), anti-NFAT (catalog number 4389S), anti-GβL (catalog number 3274S), anti-phospho-RICTOR (D30A3) (catalog number 3806), anti-phospho-TSC2 (Thr1462) (catalog number 3617S), anti-phospho-TSC2 (Tyr1561) (catalog number 3614S), anti-TSC2 (catalog number 3990S), mTOR pathway kit (catalog number 9964S) from Cell Signaling, anti-PROTOR1 (ab113269), anti-FLJ14213 (ab58856), anti-DNAPK (ab69527), anti-PHLPP (ab71972), anti-PHLPP2 (ab71973), anti-ERBB2 (ab2428), anti-phospho-ERBB2 (Tyr1248; ab47755), anti-KSR2 (ab72753) from Abcam, (Cambridge, United kingdom),; anti-TUBULIN (DM1A; Sigma, St Louis, MO, USA) and anti-E-CADHERIN (BD Laboratories, San Jose, CA, USA) and subsequently with horseradish peroxidase-conjugated anti-mouse, anti-rabbit or anti-goat secondary antibodies (1:10,000; (BIO-RAD, (Berkeley, CA, USA), and visualized by enhanced chemiluminescence (Thermo Scientific) or anti-mouse (DYLIGHT 680, catalog number 35518), anti-rabbit (DYLIGHT 800, catalog number 35571) and visualized with an Odyssey scanner.

For ELISA assays, levels of phosphorylated and total AKT2, AKT3, mTOR and total ERK were measured using the Sandwich ELISA Kit (Pathscan Cell Signaling Technology, Danvers, MA, USA) phospho-AKT2 (Ser474) (catalog number 7932); total AKT2 (catalog number 7930); phospho-AKT3 (Ser472) (catalog number 7942); total AKT3 (catalog number 7934); phospho-mTOR (Ser2481) (catalog number 7978); phospho-mTOR (Ser2448) (catalog number 7976); total mTOR (catalog number 7974); total p44/42 MAPK (ERK1/2) (catalog number 7050). The levels of phosphorylated and total AKT1 and phosphorylated ERK were measured using the Sandwich ELISA Antibody Pair, coating a Clear 96-well Microtest Plate (BD Laboratories, 353077, San Jose, CA, USA) with Capture Antibody according to the manufacturer’s instructions (Pathscan Cell Signaling Technology, phospho-Akt (Thr308) (catalog number 7144); phospho-AKT1 (Ser473) (catalog number 7143); total AKT1 (catalog number 7142); phospho-P44/42 MAPK (Thr202/Tyr204) (catalog number 7246)). Absorbance was measured at 450 nm using a Synergy-4 Microplate Reader (Biotek, Winooski, VT, USA). All assays with proteins from tumors were done with 5 μg of protein. In the case of proteins from liver, 5 μg was used for phosphorylated and total AKT1 and 10 μg for the rest of the assays. To monitor inter-assay variability, serial dilutions (1:2) of protein extracts from a tumor sample were assayed on each plate (3 to 50 μg).

### Determination of clinical serum parameters

A serum sample was obtained from each mouse at 3 months of age, when the animals were free of the disease. We also collected serum samples from FVB and F1 parentals. Known blood biomarkers were determined on a modular analyzer (cobas, Roche) at the University Hospital of Salamanca. The levels of alpha1-antitrypsin, albumin, alkaline phosphatase, complement components 3 and 4 (C3 and C4), creatinine, ferritin, urea, and immunoglobulin G (IgG), total proteins, glucose, total cholesterol, cholinesterase, high density lipoproteins (HDL), aspartate transaminase (AST), alanine transaminase (ALT), lactate dehydrogenase (LDH), calcium (Ca), magnesium (Mg), phosphorus (P), iron (Fe), and uric acid were determined, all with reagents from Roche.

### Mass spectrometry

#### Metabolite extraction and derivatization

For extraction, frozen samples were thawed on ice and 15 μl of serum was extracted as described previously [42] with 500 μl of a pre-cooled degassed methanol-isopropanol-water (3:3:2) mixture using a water-sonic bath (10 minutes at 4°C). The supernatant (450 μl) was collected after centrifugation (5 minutes, 15,000 rpm) and dried completely. Dried samples were stored at −80°C until further use. Metabolite derivatization was performed according to the methods developed by Fiehn and colleagues [43]. Briefly, a mixture of internal retention index (RI) markers was prepared using fatty acid methyl esters of C8, C9, C10, C12, C14, C16, C18, C20, C22, C24, C26, C28 and C30 linear chain length, dissolved in chloroform at a concentration of 0.8 mg/ml (C8 to C16) and 0.4 mg/ml (C18 to C30). One microliter of this RI mixture was added to the dried extracts. Ten microliters of a solution containing 40 mg/ml of 98% methoxyamine hydrochloride (CAS number 593-56-6, Sigma) in pyridine (silylation grade; Pierce, Rockford, IL, USA) was added and the mixture was shaken at 30°C for 60 minutes. Ninety microliters of N-Methyl-N-(trimethylsilyl)trifluoroacetamide (MSTFA) containing 1% trimethylchlorosilane (TMCS) (1 ml bottles; Pierce) was added and the mixture was shaken at 37°C for 30 minutes. After spinning the sample for 5 minutes at 15,000 rpm, the supernatant was transferred to a gas chromatography (GC) vial.

#### Gas chromatography-coupled mass spectrometry

Derivatized serum samples were handled using the Gerstel automatic liner exchange system with a multipurpose sampler, MPS 2 Dual Rail PrepStation, controlled by Maestro software, to inject 0.5 μl of sample into a Gerstel CIS cold injection system (Gerstel, Muehlheim, Germany). The injector was operated in splitless mode; the split vent was opened after 25 s. Samples were injected into the 50°C injector port, which was ramped to 250°C at 12°C/minute and held for 3 minutes. Volatilized metabolites were separated using an Agilent (Santa Clara, CA, USA) 6890 gas chromatograph controlled by Leco (St Joseph, MI, USA) ChromaTOF software. The GC was equipped with a 30-m long, 0.25-mm internal diameter Rtx5Sil-MS column (Restek, Bellefonte, PA, USA), a 0.25 mm 5% diphenyl film and an additional 10-m integrated guard column). The gradient used for separation was held at 50°C for 1 minute, after which ramps at 20°C per minute were applied up to 330°C, at which point temperature was held for 5 minutes. Mass spectrometry was performed with a Leco Pegasus III time-of-flight mass spectrometer with a 250°C transfer line temperature, electron ionization at −70 eV and an ion source temperature of 200°C. Mass spectra were acquired from *m/z* 85 to 500 at 17 spectra per second and a detector voltage of 1,800 V. Result files were preprocessed immediately after data acquisition and stored as ChromaTOF-specific *.peg files, as generic *.txt result files and additionally as generic ANDI MS *.cdf files. Metabolite identifications were made based on spectral similarity and the retention time indices using BinBase and were matched against the Fiehn mass spectral library of approximately 1,200 authentic metabolite spectra, using retention indices and mass spectrum information or the NIST05 commercial library [44,45]. Identified metabolites were reported if present in at least 50% of the samples per study group [46] and data were normalized in Matlab using unit vector normalization [47]. Analysis was focused on metabolites of known biological origin in HMDB [48]. Metabolite data sets were imported into the Matlab software (The MathWorks, Natick, MA, USA) for univariate and multivariate statistical analyses.

### Statistical analyses

Mammary tumor latency, disease duration and lifespan were compared among FVB, F1 and F1BX *ErbB2-*positive mice by the Kaplan-Meier estimator, whereas the number of tumors, lung metastases and metabolite determinations were evaluated and compared by the appropriate statistical tests specified above. Generally, *P*-values ≤0.05 were considered significant. For correlation studies, we included the Pearson or Spearman correlation coefficient (r), depending on the distribution of the data. The procedures were performed using the SPSS and JMP/SAS statistical packages. Simple correlations were visualized as networks using Cytoscape Software [49].

#### HJ-biplot

We generated biplots associated with principal component analysis [50-52]. In particular, we used HJ-biplot [52], which is an extension of the classical biplots introduced by Gabriel [51], and is an exploratory method of data analysis that looks for hidden patterns in the data matrix. The HJ-biplot is a joint representation, in a low dimensional vector space (usually a plane), of the rows and columns of X, using markers (points/vectors) for its rows and for its columns. The markers are obtained from the usual singular-value decomposition (SVD) of the data matrix. The HJ-biplot has the advantages of being a simultaneous representation and at the same time achieving an optimum quality of representation for both rows and columns, both represented on the same reference system. The statistical analyses were run using freely available Classical Biplot Software [53].

The rules for the interpretation of HJ-biplots are: 1) the distances among row markers are interpreted as an inverse function of similarities in such a way that closer markers (centers) are more similar. This property allows the identification of clusters of mice with similar profiles. 2) The lengths of the column markers (vectors) approximate the standard deviation of the variables. 3) The cosines of the angles among the column vectors approximate the correlations among variables in such a way that small acute angles are associated with variables with high positive correlations; obtuse angles close to a right angle are associated with variables with high negative correlations, and right angles are associated with non-correlated variables. In the same way, the cosines of the angles among the variable markers and the axes (principal components) approximate the correlations between them. 4) The order of the orthogonal projections of the row markers (points) onto a column marker (vector) approximates the order of the row elements (values) in that column. The larger the projection of an individual point onto a variable vector, the more this center deviates from the mean of that variable.

Several measurements are essential for a correct HJ-biplot interpretation [51]. For example, the relative contribution of the factor to the element is the relative variability of the variable explained by a factor or dimension. Also, for a point (row or column) on a factorial plan, the quality of representation can be defined by adding the two relative contributions of these factors to the element. Only points with a high quality of representation can be interpreted properly. These indices are indicated for the rows and for the columns of the data matrix.

#### Prediction models

We used the Cox proportional hazards model to identify independent prognostic factors in our cohort of mice. A prognostic index was constructed with the variables that predict tumor latency and survival with tumor (disease duration), using the median as cutoff to categorize these variables. To generate the coefficients of the prognostic index, we used B coefficients derived from the Cox model [54]. Mice were ranked according to their risk score and divided into two groups using the median risk score as cutoff.

#### Linkage analysis

Linkage analysis was carried out using the interval mapping with the expectation maximization algorithm [55] and R/qtl software [56]. The criteria for significant and suggestive linkages for single markers were taken from Lander and Kruglyak [57]. Permutation tests were performed (10,000 permutations per phenotype) to determine suggestive and significant *P*-values for interaction.

## Additional files

Additional file 1: Tables S1 to S23.
**Table S1. **Pathophenotypes derived from ERBB2-induced breast cancer in mice. **Table S2.** Pair-wise associations between breast cancer pathophenotypes. **Table S3.** Clusters of F1BX mice with a different prognosis. **Table S4.** Quantitative trait loci (QTLs) associated with tumor pathophenotypes. **Table S5.** Genetic markers define each cluster of prognosis. **Table S6.** List of transcripts differentially expressed between tumors and normal mammary glands from mice. **Table S7.** Human syntenic genomic regions. **Table S8.** List of 354 mouse transcripts from the mouse signature also present in the human tumor data. **Table S9.** Molecules from signaling pathways in tumor and liver correlate with tumor pathophenotypes. **Table S10.** Levels of some components of the signaling pathways from tumors and livers in each cluster of prognosis. **Table S11.** Comparison between signaling pathways in tumors from the FVB and F1 genetic backgrounds. **Table S12.** Genomic regions associated with levels of representative molecules from signaling pathways in the F1BX tumors. **Table S13.** Comparison between molecules from signaling pathways in liver, spleen and kidney from C57BL/6, FVB, F1 and F1BX mice. **Table S14.** Genomic regions associated with levels of molecules from signaling pathways in the F1BX livers. **Table S15.** Pairwise associations between molecules from signaling pathways in ERBB2 tumors from F1BX mice. **Table S16.** Pairwise associations between molecules from signaling pathways in mouse livers. **Table S17.** Pairwise correlations between molecules from signaling pathways in ERBB2 tumors and livers from F1BX mice. **Table S18.** Subphenotypes related to the levels of serum metabolites associated with tumor pathophenotypes. **Table S19.** Serum clinical biochemical markers associated with tumor pathophenotypes. **Table S20.** Levels of serum metabolites among clusters of prognosis. **Table S21.** Genomic regions associated with the levels of several metabolites simultaneously. **Table S22.** Prediction models. **Table S23.** Genetic marker peaks common to some tQTLs, tsQTLs, LsQTLs, and mQTLs. Additional file 2:
**Figures S1 to S9. Figure S1.** Localization of FVB and F1 mice in the clusters of prognosis. **Figure S2.** Similarity between human and mouse tumors at the gene expression level. **Figure S3.** RNA expression analysis in tumors. **Figure S4.** Distribution of tumors from other clusters along the seven unsupervised clusters formed with the mouse gene expression signature (MCMS) shown in Figure 3A. **Figure S5.** Evaluation of signaling pathways in tumors and livers from F1BX mice. **Figure S6.** Analysis of pAKT pathway expression in the tumors. **Figure S7.** Serum metabolites are associated with cancer progression before disease onset. **Figure S8.** Signaling levels in tumor, liver, spleen and kidney from FVB and F1 mice. **Figure S9.** Hypothesis to identify part of the missing heritability. Additional file 3:
**Supplementary references for Tables S7 and S18.**
Additional file 4:
**Cystoscape document with all the information from Figure** 8 **A.** It includes network representation with tumor pathophenotypes, tumor and liver cell signaling, serum markers from mass spectrometry and clinical biochemical markers.

## Abbreviations

bp: base pair
ELISA: enzyme-linked immunosorbent assays
GC: gas chromatography
LsQTL: liver signaling quantitative trait locus
MCHS: mouse clusters from human signature
MCMS: mouse clusters from mouse signature
MMTV: mouse mammary tumor virus
mQTL: metabolic quantitative trait locus
PCR: polymerase chain reaction
QTL: quantitative trait locus
SNP: single-nucleotide polymorphism
tQTL: tumor quantitative trait locus
tsQTL: tumor signaling quantitative trait locus
