# Supplementary material for: Unraveling heterogeneous susceptibility and the evolution of breast cancer using a systems biology approach
Source: Genome Biol. 2015 Feb 21;16(1):40. doi: 10.1186/s13059-015-0599-z (PMC4389302; doi:10.1186/s13059-015-0599-z)
Supplement: Additional file 1: Tables S1 to S23. — Table S1. Pathophenotypes derived from ERBB2-induced breast cancer in mice. Table S2. Pair-wise associations between breast cancer pathophenotypes. Table S3. Clusters of F1BX mice with a different prognosis. Table S4. Quantitative trait loci (QTLs) associated with tumor pathophenotypes. Table S5. Genetic markers define each cluster of prognosis. Table S6. List of transcripts differentially expressed between tumors and normal mammary glands from mice. Table S7. Human syntenic genomic regions. Table S8. List of 354 mouse transcripts from the mouse signature also present in the human tumor data. Table S9. Molecules from signaling pathways in tumor and liver correlate with tumor pathophenotypes. Table S10. Levels of some components of the signaling pathways from tumors and livers in each cluster of prognosis. Table S11. Comparison between signaling pathways in tumors from the FVB and F1 genetic backgrounds. Table S12. Genomic regions associated with levels of representative molecules from signaling pathways in the F1BX tumors. Table S13. Comparison between molecules from signaling pathways in liver, spleen and kidney from C57BL/6, FVB, F1 and F1BX mice. Table S14. Genomic regions associated with levels of molecules from signaling pathways in the F1BX livers. Table S15. Pairwise associations between molecules from signaling pathways in ERBB2 tumors from F1BX mice. Table S16. Pairwise associations between molecules from signaling pathways in mouse livers. Table S17. Pairwise correlations between molecules from signaling pathways in ERBB2 tumors and livers from F1BX mice. Table S18. Subphenotypes related to the levels of serum metabolites associated with tumor pathophenotypes. Table S19. Serum clinical biochemical markers associated with tumor pathophenotypes. Table S20. Levels of serum metabolites among clusters of prognosis. Table S21. Genomic regions associated with the levels of several metabolites simultaneously. Table S22. Prediction models. Table S23. Genetic marker [file 13059_2015_599_MOESM1_ESM.pdf]

Additional File 1

Unraveling heterogeneous susceptibility and the evolution of breast cancer using a systems biology approach

Andres Castellanos-Martin, *et al.*

Supplementary Tables Index……………………………..……….........Page 1

Table S1. Pathophenotypes studied in ERBB2-induced breast cancer in FVB, F1 and F1BX mice.
[Related to Figure 1B]……………………………………………………... Page 3

Table S2. Pairwise associations between different breast cancer pathophenotypes in F1BX mice.
[Related to Figure 1C]...Page 4

Table S3. Clusters of F1BX mice with different global prognosis obtained after HJ-biplot analysis.
[Related to Figures 1E and 7C].. Page 5

Table S4. QTL associated with variability of different tumor pathophenotypes.
[Related to Figure 2A]………………………………………………………… Page 6

Table S5. Genetic markers define each different prognosis cluster.
[Related to Figures 2C and 6C]……………………………………………….. Page 7

Table S6. List of transcripts differentially expressed between tumors and normal mammary glands from mice.
[Related to Figure 3A]……..………………………………………………...Page 8

Table S7. Human genomic regions syntenic to the mouse regions identified in the backcross population.
[Related to Figure 2]…………………………………………………………....Page 21

Table S8. List of 354 mouse transcripts from the mouse signature also present in the human data.
[Related to Figure 3C and Figure S3A in Additional file 2]……...................Page 22

Table S9. Some representative molecules from signaling pathways in tumors (red) and livers (green) correlated with different tumor pathophenotypes in the F1BX mice.
[Related to Figure 4C]…………………………………………………….....Page 27

Table S10. Level of different components of signaling pathways in tumors (red) and livers (green) in each different prognosis cluster.
[Related to Figure 4E and Figures S5C, S5D, and S5Ein Additional file 2]………………………….... Page 28

Table S11. Comparison between levels of some representative molecules from signaling pathways in tumors from FVB and F1 genetic backgrounds.
[Related to Figure S8 in Additional file 2]…………………………............Page 29

Table S12. Genomic regions associated with variable levels of some representative molecules from signaling pathways in the F1BX tumors.
[Related to Figure 6]………………………………………………………....Page 30

Table S13. Comparison between levels of different proteins from signaling pathways in liver, spleen and kidney from C57BL/6, FVB, F1 and F1BX genetic background mice.
[Related to Figure S8 in Additional file 2]………………………………... Page 31

Table S14. Genomic regions associated with variable levels of some proteins from signaling pathways in the F1BX livers.
[Related to Figure 6]…………………………………………………………Page 32

Table S15. Pairwise associations between levels of some representative molecules from cell signaling pathways in ERBB2 tumors from F1BX mice…………………………………………...Page 33

Table S16. Pairwise associations between levels of some molecules from cell signaling pathways in mouse livers from F1BX………………………………………………………………… Page 34

Table S17. Pairwise correlations between levels of some molecules from cell signaling pathways in ERBB2 tumors and livers from F1BX mice………………………………………………….Page 35

Table S18. Subphenotypes related to levels of different serum metabolites associated with tumor pathophenotypes obtained by mass spectrometry.
[Related to Figure S7 in Additional file 2]……….................................... Page 36

Table S19. Subphenotypes of several typical serum clinical biochemical markers associated with tumor pathophenotypes
[Related to Figure S7 in Additional file 2]……………………………........Page 37

Table S20. Different serum metabolites levels among clusters of prognosis based on tumor pathophenotypes.
[Related to Figure 7C]…………………………………………………... Page 38

Table S21. Genomic regions associated with the variable levels of different metabolites simultaneously.
[Related to Figure 6]………………………………………………………....Page 39

Table S22. Prediction models.
[Related to Figures 8B and 8C]…...Page 40

Table S23. Genetic marker peaks common to some tQTLs, tsQTLs, LsQTLs, and mQTLs.
[Related to Figures 2 and 5]………….. Page 41

Table S1. Pathophenotypes studied in ERBB2-induced breast cancer in FVB, F1 and F1BX mice. (#) We compared FVB, F1 and F1BX mice using the Kruskal-Wallis test and performed pairwise comparisons with the Dunn Method for Joint Ranking. We compared temporal stages using the Log-Rank Test. We performed contingency analysis of incidence and multiplicity of tumors and metastasis using the Pearson Chi-squared Test. SEM, standard error of the mean. IR, interquartile range. Blue: *P*-values with statistical trend. n.s., non-significant *P*-values. We represent some examples from these data in Figure 1B.

Pathophenotypes	FVB	F1	F1BX	*P*- value (#)	Pairwise Comparisons		
					F1-FVB	F1-F1BX	FVB-F1BX
A. <u>TEMPORAL STAGES</u>							
**1. Tumor Latency** (weeks)							
Latency Interval	29.71-77.43	52.57-98.71	23.86-114.29				
Average (SEM)<br>Median (IR)<br>(Proportion of sick mice)	46.36 (2.13)<br>45.71 (14.85)<br>(26/26)	80.44 (2.68)<br>78.5 (20.04)<br>(20/26)	61.61 (1.54)<br>59.36 (21.58)<br>(124/147)	<0.0001	<0.0001	0.002	<0.0001
**2. Duration of the Disease** (weeks)							
Disease Duration Interval	0.29-18.00	4.43-17.00	3.29-32.00				
Average (SEM)<br>Median (IR)<br>(Number of mice)	11.57 (1.01)<br>12.43 (9.03)<br>(*n* =26)	11.11 (0.83)<br>11.43 (5.65)<br>(*n* =20)	12.64 (0.46)<br>12.14 (5.79)<br>(*n* =124)	n.s.			
**3. Lifespan (weeks)**							
Lifespan Interval	40.71- 94.29	61.71-109.57	41.57-124.47				
Average (SEM)<br>Median (IR)<br>(Number of mice)	57.93 (2.18)<br>58.29 (12.86)<br>(*n* =26)	91.98 (2.09)<br>91.93 (16.61)<br>(*n* =26)	79.62 (1.68)<br>76.86 (27.00)<br>(n=147)	<0.0001	<0.0001	0.029	<0.0001
B. <u>TUMOR TRAITS</u>							
**1. Tumor Number**							
Incidence<br>(Proportion)	100.00%<br>(26/26)	76.92%<br>(20/26) (*1)	84.35%<br>(124/147)	0.048			
Multiplicity<br>(Proportion)	84.62%<br>(22/26)	55.00%<br>(11/20)	58.06%<br>(72/124)	0.022			
Absolute Number	1.00-7.00	0.00-5.00 (*2)	0.00-7.00 (*3)				
Average (SEM)<br>Median (IR)	3.65 (0.35)<br>4.00 (3.00)	1.5 (0.24)<br>1.00 (1.5)	1.79 (0.12)<br>1.00 (1.00)	<0.0001	<0.0001	n.s.	<0.0001
**2. Local Growth Rate**							
Average (SEM)<br>Median (IR) (*4)	0.02 (0.004)<br>0.03 (0.02)	0.04 (0.005)<br>0.04 (0.03)	0.04 (0.002)<br>0.04 (0.03)	0.003	0.088	n.s.	0.002
**3.Tumor Weight** (1st tumor)							
Average (SEM)<br>Median (IR) (grams)	4.17 (0.54)<br>3.94 (2.05)	4.98 (0.78)<br>4.30 (2.08)	6.18 (0.35)<br>5.70 (5.15)	0.047	n.s.	n.s.	0.099
**4. Metastasis**							
Incidence	71.43%<br>(15/21) (*5)	38.89%<br>(7/18) (*6)	65.52%<br>(76/116) (*7)	n.s.			
Multiplicity	73.33%<br>(11/15)	71.43%<br>(5/7)	80.26%<br>(61/76)	n.s.			
Absolute Number	0.00-26.00	0.00-50.00	0.00-87.00				
Average (SEM)<br>Median (IR)	6.14 (1.78)<br>2.00 (13.00)	4.28 (2.82)<br>0.00 (2.25)	7.39 (1.32)<br>2.00 (7.00)	0.089	n.s.	n.s.	n.s.

(*1) The last three F1 mice had to be euthanized without tumors at 100, 101.5 and 106 weeks of age, respectively, and the last F1 mouse with tumor died at 109.5 weeks. (*2) A single F1 mouse developed 4 tumors. (*3) Two and six F1BX mice developed 5 and 6 tumors, respectively. (*4) Slope arbitrary units. (*5) Four FVB mice had missing information for metastasis. (*6) Notice that 6 F1 mice did not develop tumors. Two additional F1 mice had missing information for metastasis. (*7) We censored eight F1BX mice without metastasis information.

Table S2. Pairwise associations between different breast cancer pathophenotypes in F1BX mice. n.s., not statistically significant. We included these associations in Figure 1C.

	Latency	Duration of Disease	Number of Tumors	Tumor Weight	Tumor Volume	Tumor Growth Rate	Tumor Growth Speed	Number of Metastasis
Lifespan	r = 0.955<br>n = 124<br>P < 0.0001	n.s.	r = -0.418<br>n = 147<br>P < *0.0001*	r = -0.352<br>n = 105<br>P = 0.0002	r = -0.210<br>n = 120<br>P = 0.022	r = -0.225<br>n = 104<br>P = 0.022	r =*-0.223*<br>n = 120<br>P = 0.014	r = -0.199<br>n = 116<br>P = 0.033
Latency		r = -0.310<br>n = 124<br>P = 0.0005	n.s.	r = -0.346<br>n = 105<br>P = 0.0003	r = -0.254<br>n = 120<br>P = 0.005	n.s.	n.s.	r = -0.266<br>n = 116<br>P = 0.004
Duration of Disease			n.s.	n.s.	n.s.	r = -0.599<br>n = 104<br>P < 0.0001	r =*-0.197*<br>n = 120<br>P = 0.031	r = 0.301<br>n = 116<br>P = 0.001
Number of Tumors				n.s.	n.s.	n.s.	n.s.	n.s.
Tumor Weight					r = 0.726<br>n = 104<br>P < 0.0001	n.s.	r = 0.663<br>n = 104<br>P < *0.0001*	r = 0.270<br>n = 104<br>P = 0.006
Tumor Volume						n.s.	r = 0.875<br>n = 120<br>P < *0.0001*	n.s.
Tumor Growth Rate							r = 0.466<br>n = 104<br>P < *0.0001*	n.s.

Table S3. Clusters of F1BX mice with different global prognoses obtained after HJ-biplot analysis. (*) ANOVA; (#) Tukey´s post-test. Upper values: mean and SEM. Lower values: median and IR. n/a, not applicable in mice without tumors. We represent these values in Figures 1E and 7C.

	**Cluster 1**<br>**Bad**<br>***n*** **=25**	**Cluster 2**<br>**Middle-Bad**<br>***n*** **=59**	**Cluster 3**<br>**Middle-Good**<br>***n*** **=26**	**Cluster 4**<br>**Good**<br>***n*** **=14**	**Cluster 5**<br>**Excellent**<br>***n*** **=23**	***P*** **-value** (*)	**Pairwise Comparison**		**Post-test**<br>***P*** **-value** (#)
**Lifespan**	57.22±1.63<br>56.14 (11.285)	73.27±1.59<br>70.00 (14.28)	76.97±1.43<br>78.71 (12.75)	103.69±2.63<br>101.07	108.58±3.<br>110.14(30.	< 0.0001	Cluster 5	Cluster 1	< 0.0001
							Cluster 4	Cluster 1	< 0.0001
							Cluster 5	Cluster 2	< 0.0001
							Cluster 4	Cluster 2	< 0.0001
							Cluster 3	Cluster 1	< 0.0001
							Cluster 5	Cluster 3	< 0.0001
							Cluster 2	Cluster 1	< 0.0001
							Cluster 4	Cluster 3	< 0.0001
**Latency**	47.53±1.72<br>47.14 (11.575)	57.18±1.66<br>54.57 (13.43)	67.11±1.06<br>68 (9.1425)	95.19±2.79<br>93.57	n/a	< 0.0001	Cluster 4	Cluster 1	< 0.0001
							Cluster 4	Cluster 2	< 0.0001
							Cluster 3	Cluster 1	< 0.0001
							Cluster 3	Cluster 2	0.0006
							Cluster 2	Cluster 1	0.0011
							Cluster 4	Cluster 3	< 0.0001
**Duration of Disease**	9.70±0.53<br>9.58 (4.07)	16.09±0.63<br>15.00 (6.29)	9.86±0.54<br>10.00 (4.75)	8.51±0.79<br>8.50 (4.07)	n/a	< 0.0001	Cluster 2	Cluster 1	< 0.0001
							Cluster 3	Cluster 2	< 0.0001
							Cluster 4	Cluster 2	< 0.0001
**Tumor Growth Rate**	0.07±0.00<br>0.06 (0.035)	0.03±0.00<br>0.03(0.01)	0.05±0.00 0.05<br>(0.02)	0.05±0.00<br>0.05 (0.01)	n/a	< 0.0001	Cluster 2	Cluster 1	< 0.0001
							Cluster 3	Cluster 2	< 0.0001
							Cluster 4	Cluster 2	< 0.0001
							Cluster 1	Cluster 3	< 0.0001
							Cluster 1	Cluster 4	0.0007
**Tumor Weight**	11.00±0.95<br>12 (3.6)	5.61±0.36<br>5.65 (3.92)	5.61±0.73<br>5.40 (4.21)	3.84±0.75<br>2.55 (4.72)	n/a	< 0.0001	Cluster 4	Cluster 1	< 0.0001
							Cluster 2	Cluster 1	< 0.0001
							Cluster 3	Cluster 1	< 0.0001
**Number of Tumors**	1.48±0.18<br>1 (1)	2.73±0.19<br>2(2)	1.77±0.17<br>2(1)	1.36±0.17<br>1(1)	n/a	< 0.0001	Cluster 2	Cluster 1	0.0001
							Cluster 3	Cluster 2	0.0046
							Cluster 4	Cluster 2	0.001
**Number of Metastasis**	15.65±5.45<br>4 (19.5)	8.12±1.65<br>4.00 (8)	2.40±0.92<br>0.00 (3.5)	1.00±0.48<br>0.00 (2)	n/a	0.0004	Cluster 4	Cluster 1	0.0166
							Cluster 3	Cluster 1	0.0085

Table S4. QTLs associated with the variability of different tumor pathophenotypes. tQTL: tumor QTL. Chr, chromosome. We represent a summary of these data in Figure 2A.

Pathophenotype	tQTL	Chr.	Marker Peak	Confidence Interval (0.05)	Location (cM)	Location (Mb)	LOD Score	Average ± SEM FF	Average ± SEM FB	Higher Effect Allele
Latency				rs13476573	49.41	73.18	0.2			
	**tQTL 1**	2	c2.loc87.5		90.59	158.98	1.6	64.08 ± 3.44	76.97 ± 2.94	C57BL/6
				rs13476913	102.57	170.65	1.5			
				rs3724197	4.68	7.96	1.6			
	**tQTL 2**	7	rs3724197		4.68	7.96	1.6	64.28 ± 3.48	76.67 ± 2.93	C57BL/6
				c7.loc60	64.68	124.23	0.0			
				c13.loc25	27.19	42.02	1.6			
	**tQTL 3**	13	gnf13.057.501		40.08	58.83	2.6	65.05 ± 3.17	77.92 ± 3.15	C57BL/6
				c13.loc60	62.19	102.40	1.7			
				c18.loc10	16.59	24.22	0.3			
	**tQTL 4**	18	rs4231934		55.31	74.00	1.6	78.89 ± 3.47	65.14 ± 3.24	FVB
				rs4231934	55.31	74.00	1.6			
Number of Tumors				rs6215262	2.19	3.55	0.3			
	**tQTL 5**	13	c13.loc47.5		49.69	79.65	3.1	2.218975 ± 0.16	1.36 ± 0.16	FVB
				rs6316705	70.93	112.85	1.1			
Tumor Incidence				c4.loc7.5	12.17	20.43	0.9			
	**tQTL 6**	4	rs13477617		16.31	27.10	2.0	91.9%	73.8%	FVB
				rs13478069	94.61	153.40	1.2			
				c13.loc25	27.19	42.02	1.6			
	**tQTL 3**	13	gnf13.057.501		40.08	58.83	2.5	94.4%	74.3%	FVB
				c13.loc62.5	64.69	103.94	1.4			
				c15.loc12.5	14.46	25.58	0.3			
	**tQTL 7**	15	rs6326790		53.91	90.94	1.8	76.0%	93.0%	C57BL/6
				rs6326790	53.91	90.94	1.8			
Tumor Volume				rs3724197	4.68	7.96	0.3			
	**tQTL 8**	7	rs3696018		24.85	44.77	1.6	6781.84 ± 882.45	9822.41 ± 671.44	C57BL/6
				rs6216320	73.10	133.56	0.5			
Tumor Weight				rs3703275	16.29	21.42	0.9			
	**tQTL 9**	17	rs13459154		43.01	64.44	1.9	7.35 ± 0.52	5.30 ± 0.45	FVB
				c17.loc32.5	48.79	70.82	1.0			
Tumor Growth Rate				rs3672461	4.34	7.52	0.2			
	**tQTL 10**	14	rs3698545		68.30	113.99	1.8	0.04 ± 0.003	0.05 ± 0.003	C57BL/6
				cel-14_116404928	68.79	116.39	1.7			
				rs13459150	18.10	24.80	1.2			
	**tQTL 9**	17	c17.loc25		41.29	60.57	1.5	0.05 ± 0.003	0.038 ± 0.003	FVB
				c17.loc35	51.29	72.75	0.9			
Tumor Growth Average Speed				rs3716790	12.33	18.07	0.3			
	**tQTL 11**	11	rs13481230		75.32	110.83	1.8	862.61 ± 64.89	576.55 ± 72.95	FVB
				rs13481230	75.32	110.83	1.8			
Number of Metastasis				c5.loc2.5	18.08	29.19	1.2			
	**tQTL 12**	5	c5.loc20		35.58	61.39	2.2	3.50 ± 1.79	11.28 ± 1.79	C57BL/6
				rs3662161	60.53	116.56	1.2			
				cel-x_67980997	36.10	67.99	0.7			
	**tQTL 13**	X	rs13483994		49.82	124.29	1.6	9.17 ± 1.54	3.09 ± 2.39	FVB
				gnfx.146.867	73.95	156.38	1.1			
Metastasis Incidence				rs13475706	2.04	6.65	1.0			
	**tQTL 14**	1	gnf01.037.906		21.72	41.15	2.2	50%	78.1%	C57BL/6
				c1.loc37.5	39.54	73.82	0.5			
				rs6159963	15.58	26.81	1.1			
	**tQTL 12**	5	rs6256504		23.20	37.72	2.2	52.5%	80%	C57BL/6
				c5.loc35	50.58	97.06	1.0			
Lifespan				rs3724197	4.68	7.96	1.9			
	**tQTL 2**	7	rs3724197		4.68	7.96	1.9	75.59 ± 2.95	86.86 ± 2.48	C57BL/6
				c7.loc65	69.68	129.76	0.0			
				c13.loc22.5	24.69	39.48	1.5			
	**tQTL 3**	13	gnf13.057.501		40.08	58.83	2.5	76.15 ± 2.70	87.98 ± 2.65	C57BL/6
				c13.loc60	62.19	102.40	1.6			
				c15.loc12.5	14.46	25.58	0.5			
	**tQTL 7**	15	c15.loc50		51.96	88.63	1.5	86.66 ± 2.68	77.52 ± 2.74	FVB
				rs6326790	53.91	90.94	1.5			
				c18.loc12.5	19.09	26.19	0.5			
	**tQTL 4**	18	rs4231934		55.31	74.00	1.9	89.57 ± 2.95	75.67 ± 2.78	FVB
				rs4231934	55.31	74.00	1.9			

Table S5. Genetic markers define each different prognosis cluster. Percentages indicate the proportion of mice that carry genetic markers in heterozygosis in each cluster. Note that most of the genetic markers identified seem to correspond to some tQTLs described in Table S4. **(a)** This table compares mice without and with tumors. **(b)** This section shows the comparison among all the prognosis clusters identified. We represent these values in Figure 2C and, partially, in Figure 6C. n.s., not statistically significant. *P*-values were calculated with the Phi-coefficient.

a

Marker	Chr.	Location (Mb)	tQTL	Mice Without Tumors (*n* = 23)	Mice With Tumors (*n* = 124)	*P*-value
rs3653593	4	22.88	tQTL 6	64%	38.70%	0.02
rs13477617		27.11		68%	36.30%	0.003
rs4138630		41.75		64%	38.70%	0.02
rs6386918	4	138.22		32%	54%	0.044
rs3023025		141.64		32%	57.30%	0.021
rs13478069		153.39		32%	57.30%	0.021
rs3724197	7	7.95	tQTL 2	84%	54%	0.005
rs3704385	8	25.08		40%	61.30%	0.049
gnf08.026.892		27.24		40%	62.10%	0.041
rs13479753		49.71		36%	58.90%	0.036
cel8_51076519		51.08		36%	58.90%	0.036
rs3693942	13	54.04	tQTL3	84%	46%	0.001
gnf13.057.501		58.59		84%	45.20%	<0.001
rs13481839		59.56		80%	45.20%	0.001
rs3712144	13	63.57	tQTL5	80%	44.40%	0.001
gnf13.092.499		88.55		80%	45.20%	0.001
rs6316705		112.89		72%	46%	0.018
rs6326790	15	90.94	tQTL7	28%	54%	0.018
rs3657117	17	67.48	tQTL9	36%	58.10%	0.043
rs13483765	X	49.19		8%	28.20%	0.033
cel-x_67980997		67.98		8%	26.60%	0.048

b

Marker	Chr.	Location (Mb)	tQTL	Cluster 1 (*n* = 25)	Cluster 2 (*n* = 59)	Cluster 3 (*n* = 26)	Cluster 4 (*n* = 14)	Cluster 5 (*n* = 23)	*P*-value
rs13477617	4	27.11	tQTL 6	48%	30.50%	34.60%	42.90%	68%	0.015
rs3714811	4	122.58		56%	40.70%	73.10%	64.30%	32%	0.025
rs3023025		141.64		52%	50.80%	73.10%	64.30%	32%	0.05
rs3724197	7	7.95	tQTL 2	52%	50.80%	53.80%	71.40%	84%	0.044
rs13479622	8	16.27		72%	54.20%	73.10%	35.70%	40%	0.03
gnf08.021.719		22		68%	54.20%	76.90%	35.70%	40%	0.025
rs13479735		44.68		68%	52.50%	76.90%	42.90%	40%	0.041
rs13479753		49.71		64%	52.50%	80.80%	35.70%	36%	0.008
cel8_51076519		51.08		64%	52.50%	80.80%	35.70%	36%	0.008
rs3685424		56.55		64%	49.20%	76.90%	35.70%	36%	0.016
rs8253516		67.91		60%	47.50%	76.90%	28.60%	44%	0.023
rs6296891		79.15		60%	49.20%	76.90%	28.60%	48%	0.034
rs3693942	13	54.04	tQTL3	44%	40.00%	57.70%	50%	84%	0.006
gnf13.057.501		58.59		44%	39%	57.70%	50%	84%	0.004
rs13481839		59.56		44%	39%	57.70%	50%	80%	0.012
rs3712144	13	63.57	tQTL5	44%	37.30%	57.70%	50%	80%	0.0508
gnf13.092.499		88.55		48%	35.60%	61.50%	50%	80%	0.004
rs13459154	17	64.22	tQTL9	32%	59.30%	53.80%	85.70%	40%	0.01
rs3657117		67.48		36%	59.30%	65.40%	78.60%	36%	0.018
rs3670607		77.91		36%	64.40%	61.50%	71.40%	40%	0.04
rs4231899	18	63.02	tQTL4	72%	57.60%	50%	21.40%	52%	0.045
rs6320743		65.69		72%	57.60%	46.20%	21.40%	44%	0.027
rs4231934		74.8		72%	61%	46.20%	28.60%	36%	0.018

Table S6. List of transcripts differentially expressed between tumors and normal mammary glands from mice. Green: upregulated transcripts. Red: downregulated transcripts. This signature differentiated seven unsupervised clusters represented in Figure 3A.

Affymetrix Probe ID	Genes in Mouse Signature	Mouse Gene Accession	mRNA Accession
10490273	100043387	NM_001099327	NM_001099327
10345089	---	---	mmu-mir-30c-2
10349634	---	---	ENSMUST00000097584
10350341	---	---	mmu-mir-181b-1
10352916	---	---	mmu-mir-29b-2
10362208	---	---	---
10362289	---	---	---
10366739	---	---	GENSCAN00000043523
10388269	---	---	ENSMUST00000119400
10397000	---	---	ENSMUST00000082780
10398416	---	---	mmu-mir-487b
10401931	---	---	AF263910
10401933	---	---	AF043690
10401937	---	---	AF263910
10402994	---	---	ENSMUST00000095374
10405779	---	---	mmu-mir-23b
10405781	---	---	mmu-mir-27b
10405783	---	---	mmu-mir-24-1
10406461	---	---	mmu-mir-9-2
10406896	---	---	ENSMUST00000083744
10407022	---	---	ENSMUST00000095488
10414711	---	---	X14387
10414747	---	---	ENSMUST00000103587
10417068	---	---	---
10419726	---	---	---
10422942	---	---	ENSMUST00000082632
10433195	---	---	GENSCAN00000044232
10435787	---	---	ENSMUST00000093651
10436594	---	---	AK076276
10436600	---	---	mmu-mir-99a
10436602	---	---	mmu-let-7c-1
10436662	---	---	mmu-mir-155
10438415	---	---	M34598
10456291	---	---	mmu-mir-122
10458888	---	---	ENSMUST00000082886
10460146	---	---	BC028925
10462086	---	---	AK164996
10466344	---	---	ENSMUST00000087769
10471880	---	---	mmu-mir-181b-2
10473471	---	---	ENSMUST00000121914
10477120	---	---	AK040886
10485357	---	---	ENSMUST00000099683
10491897	---	---	ENSMUST00000083084
10491960	---	---	ENSMUST00000083136
10492452	---	---	GENSCAN00000018345
10497998	---	---	GENSCAN00000029574
10504753	---	---	AF043688
10504759	---	---	AF043690
10504761	---	---	AF043688
10506052	---	---	ENSMUST00000082949
10506820	---	---	ENSMUST00000083365
10507326	---	---	ENSMUST00000083251
10507473	---	---	AK154548
10507475	---	---	ENSMUST00000093716
10507477	---	---	ENSMUST00000093725
10514185	---	---	---
10515280	---	---	---
10519475	---	---	---
10525829	---	---	---
10525914	---	---	---
10529091	---	---	ENSMUST00000101411
10534716	---	---	---
10536111	---	---	ENSMUST00000099245
10538880	---	---	U65535
10538890	---	---	AF043688
10538892	---	---	AF043690
10538921	---	---	BC019760
10538924	---	---	AF466768
10542878	---	---	---
10544715	---	---	mmu-mir-148a
10545177	---	---	ENSMUST00000103331
10545245	---	---	ENSMUST00000103392
10546889	---	---	ENSMUST00000083375
10548752	---	---	---
10549055	---	---	---
10550200	---	---	ENSMUST00000102013
10551195	---	---	ENSMUST00000082604

10559818	---	---	---
10564209	---	---	ENSMUST00000097241
10568731	---	---	---
10571599	---	---	---
10572046	---	---	ENSMUST00000098716
10578959	---	---	ENSMUST00000118929
10582882	---	---	---
10582884	---	---	ENSMUST00000099046
10582888	---	---	ENSMUST00000099042
10582890	---	---	ENSMUST00000099042
10582896	---	---	---
10582899	---	---	ENSMUST00000099035
10582916	---	---	---
10584580	---	---	ENSMUST00000082857
10587778	---	---	ENSMUST00000083947
10596381	---	---	ENSMUST00000122710
10598023	---	---	NC_005089
10598027	---	---	NC_005089
10598041	---	---	NC_005089
10598062	---	---	NC_005089
10598064	---	---	NC_005089
10598073	---	---	NC_005089
10598075	---	---	NC_005089
10598077	---	---	NC_005089
10598081	---	---	NC_005089
10598083	---	---	NC_005089
10598089	---	---	NC_005089
10598794	---	---	---
10605559	---	---	---
10607950	---	---	ENSMUST00000101072
10608650	---	---	NM_010399.2
10608668	---	---	NM_001081285.1
10608680	---	---	NM_010395.5
10608712	---	---	NM_138758.1
10439018	0610012G03Rik	NR_027897	NR_027897
10369806	1700040L02Rik	BC087900	BC087900
10460251	1700055N04Rik	AK081788	AK081788
10408647	1810022C23Rik	BC014724	BC014724
10501048	2010016I18Rik	AK140363	AK140363
10599581	2610018G03Rik	NM_133729	NM_133729
10490302	2810021G02Rik	NM_001162922	NM_001162922
10491977	2810046L04Rik	NM_173382	NM_173382
10436590	2810055G20Rik	AK148800	AK148800
10601846	2900062L11Rik	NR_003642	NR_003642
10601588	3110007F17Rik	BC027572	BC027572
10601595	3110007F17Rik	BC027572	BC027572
10601598	3110007F17Rik	BC027572	BC027572
10491825	3110057O12Rik	BC145212	BC145212
10474057	4631405J19Rik	BC100298	BC100298
10459643	4930503L19Rik	BC057927	BC057927
10498053	4930583H14Rik	NM_026358	NM_026358
10591022	4931406C07Rik	BC016078	BC016078
10551423	4933426I21Rik	BC024577	BC024577
10501374	5330417C22Rik	NM_001033304	NM_001033304
10549171	5730419I09Rik	NM_029081	NM_029081
10527649	6330406I15Rik	BC116246	BC116246
10584561	9030425E11Rik	NM_133733	NM_133733
10530259	9130230L23Rik	NR_027961	NR_027961
10565152	9330120H11Rik	ENSMUST00000098326	ENSMUST00000098326
10369171	9530009G21Rik	AK079179	AK079179
10586718	9530091C08Rik	NM_177159	NM_177159
10541354	A2m	NM_175628	NM_175628
10441339	A630089N07Rik	ENSMUST00000099483	ENSMUST00000099483
10344879	A830018L16Rik	NM_001160369	NM_001160369
10543333	Aass	NM_013930	NM_013930
10392522	Abca8a	NM_153145	NM_153145
10519555	Abcb1b	NM_011075	NM_011075
10355742	Abcb6	NM_023732	NM_023732
10436304	Abi3bp	NM_001014423	NM_001014423
10562314	Abpb	NM_001100464	NM_001100464
10417544	Acox2	NM_053115	NM_053115
10399820	Acp1	NM_001110239	NM_001110239
10596303	Acpp	NM_019807	NM_019807
10488482	Acss1	NM_080575	NM_080575
10467124	Acta2	NM_007392	NM_007392
10489391	Ada	NM_007398	NM_007398
10440522	Adamts1	NM_009621	NM_009621
10531173	Adamts3	NM_001081401	NM_001081401
10531175	Adamts3	NM_001081401	NM_001081401
10531177	Adamts3	NM_001081401	NM_001081401
10531179	Adamts3	NM_001081401	NM_001081401
10531183	Adamts3	NM_001081401	NM_001081401
10531187	Adamts3	NM_001081401	NM_001081401

10531191	Adamts3	NM_001081401	NM_001081401
10531193	Adamts3	NM_001081401	NM_001081401
10531195	Adamts3	NM_001081401	NM_001081401
10531197	Adamts3	NM_001081401	NM_001081401
10531201	Adamts3	NM_001081401	NM_001081401
10531203	Adamts3	NM_001081401	NM_001081401
10351551	Adamts4	NM_172845	NM_172845
10440534	Adamts5	NM_011782	NM_011782
10546450	Adamts9	NM_175314	NM_175314
10546454	Adamts9	NM_175314	NM_175314
10429029	Adcy8	NM_009623	NM_009623
10556297	Adm	NM_009627	NM_009627
10357878	Adora1	NM_001008533	NM_001008533
10464167	Adrb1	NM_007419	NM_007419
10398859	Adssl1	NM_007421	NM_007421
10374083	Aebp1	NM_009636	NM_009636
10459241	Afap1l1	NM_178928	NM_178928
10473024	Agps	NM_172666	NM_172666
10360377	AI607873	BC150711	BC150711
10471247	Aif1l	NM_145144	NM_145144
10472923	Ak3l1	NM_009647	NM_009647
10407435	Akr1c18	NM_134066	NM_134066
10439895	Alcam	NM_009655	NM_009655
10461979	Aldh1a1	NM_013467	NM_013467
10460253	Aldh3b2	ENSMUST00000100032	ENSMUST00000100032
10379153	Aldoc	NM_009657	NM_009657
10517587	Alpl	NM_007431	NM_007431
10450038	Angptl4	NM_020581	NM_020581
10423363	Ank	NM_020332	NM_020332
10467191	Ankrd1	NM_013468	NM_013468
10467115	Ankrd22	NM_024204	NM_024204
10569618	Ano1	NM_178642	NM_178642
10564818	Anpep	NM_008486	NM_008486
10545974	Antxr1	NM_054041	NM_054041
10500140	Anxa9	NM_001085383	NM_001085383
10425321	Apobec3	NM_001160415	NM_001160415
10425333	Apobec3	ENSMUST00000100423	ENSMUST00000100423
10560618	Apoc1	NM_007469	NM_007469
10430174	Apol9a	NM_173786	NM_173786
10425049	Apol9b	NM_173743	NM_173743
10538459	Aqp1	NM_007472	NM_007472
10426767	Aqp5	NM_009701	NM_009701
10512145	Aqp7	NM_007473	NM_007473
10368343	Arg1	NM_007482	NM_007482
10604713	Arhgef6	NM_152801	NM_152801
10564507	Arrdc4	NM_001042592	NM_001042592
10361091	Atf3	NM_007498	NM_007498
10438854	Atp13a4	NM_172613	NM_172613
10359689	Atp1b1	NM_009721	NM_009721
10378216	Atp2a3	NM_016745	NM_016745
10575926	Atp2c2	NM_026922	NM_026922
10538082	Atp6v0e2	NM_133764	NM_133764
10539449	Atp6v1b1	NM_134157	NM_134157
10399559	Atp6v1c2	NM_001159632	NM_001159632
10489985	Atp9a	NM_015731	NM_015731
10466127	AW112010	EF660528	EF660528
10485361	B230118H07Rik	BC025075	BC025075
10437191	B3galt5	NM_033149	NM_033149
10457733	B4galt6	NM_019737	NM_019737
10502335	Bank1	NM_001033350	NM_001033350
10485700	Bbox1	NM_130452	NM_130452
10568601	BC002195	BC002195	BC002195
10401935	BC005685	BC005685	BC005685
10504757	BC005685	BC005685	BC005685
10538901	BC005685	BC005685	BC005685
10392685	BC006965	NR_024085	NR_024085
10392687	BC006965	NR_024085	NR_024085
10455970	BC023105 // BC023105	BC023105 // BC023105	BC023105
10498710	Bche	NM_009738	NM_009738
10561063	Bckdha	NM_007533	NM_007533
10542287	Bcl2l14	NM_025778	NM_025778
10353775	Bend6	NM_177235	NM_177235
10600169	Bgn	NM_007542	NM_007542
10527323	Bhlha15	NM_010800	NM_010800
10601844	Bhlhb9	NM_198161	NM_198161
10549276	Bhlhe41	NM_024469	NM_024469
10414269	Bnip3	NM_009760	NM_009760
10439612	Boc	NM_172506	NM_172506
10450197	Btnl5	NR_004051	NR_004051
10487480	Bub1	NM_001113179	NM_001113179
10356248	C130026I21Rik	NM_175219	NM_175219
10541683	C1rb	NM_001113356	NM_001113356
10547740	C1s	NM_144938	NM_144938

10452316	C3	NM_009778	NM_009778
10458992	C330018D20Rik	ENSMUST00000025488	ENSMUST00000025488
10436106	C330027C09Rik	NM_172616	NM_172616
10543369	Cadps2	NM_153163	NM_153163
10423024	Capsl	NM_029341	NM_029341
10586591	Car12	NM_178396	NM_178396
10607738	Car5b	NM_181315	NM_181315
10518751	Car6	NM_009802	NM_009802
10511429	Car8	NM_007592	NM_007592
10504337	Car9	NM_139305	NM_139305
10474875	Casc5	NM_029617	NM_029617
10582985	Casp1	NM_009807	NM_009807
10583008	Casp12	NM_009808	NM_009808
10536499	Cav1	NM_007616	NM_007616
10536494	Cav2	NM_016900	NM_016900
10571978	Cbr4	NM_145595	NM_145595
10379524	Ccl11	NM_011330	NM_011330
10379511	Ccl2	NM_011333	NM_011333
10504127	Ccl21a	NM_011124	NM_011124
10504154	Ccl21a	NM_011124	NM_011124
10504183	Ccl21a	NM_011124	NM_011124
10512377	Ccl21a	NM_011124	NM_011124
10389231	Ccl3	NM_011337	NM_011337
10389207	Ccl5	NM_013653	NM_013653
10379518	Ccl7	NM_013654	NM_013654
10411739	Ccnb1	NM_172301	NM_172301
10515836	Ccnb1	NM_172301	NM_172301
10562637	Ccnb1	NM_172301	NM_172301
10569646	Ccnd1	NM_007631	NM_007631
10375313	Ccnjl	NM_001045530	NM_001045530
10390763	Ccr7	NM_007719	NM_007719
10458382	Cd14	NM_009841	NM_009841
10560886	Cd177	NM_026862	NM_026862
10499160	Cd1d1	NM_007639	NM_007639
10500677	Cd2	NM_013486	NM_013486
10462390	Cd274	NM_021893	NM_021893
10501063	Cd53	NM_007651	NM_007651
10474229	Cd59a	NM_001111060	NM_001111060
10548333	Cd69	NM_001033122	NM_001033122
10456005	Cd74	NM_001042605	NM_001042605
10551025	Cd79a	NM_007655	NM_007655
10501629	Cdc14a	NM_001080818	NM_001080818
10369815	Cdc2a	NM_007659	NM_007659
10439483	Cdgap	NM_020260	NM_020260
10357103	Cdh19	NM_001081386	NM_001081386
10607619	Cdkl5	NM_001024624	NM_001024624
10515090	Cdkn2c	NM_007671	NM_007671
10550994	Ceacam10	NM_007675	NM_007675
10481278	Cel	NM_009885	NM_009885
10345074	Cetn4	NM_145825	NM_145825
10450325	Cfb	NM_008198	NM_008198
10358339	Cfh	NM_009888	NM_009888
10380398	Chad	NM_007689	NM_007689
10349968	Chi3l1	NM_007695	NM_007695
10601303	Chic1	NM_009767	NM_009767
10540298	Chl1	NM_007697	NM_007697
10538356	Chn2	NM_023543	NM_023543
10407591	Chrm3	NM_033269	NM_033269
10365290	Chst11	NM_021439	NM_021439
10507726	Cited4	NM_019563	NM_019563
10475324	Ckmt1	NM_009897	NM_009897
10502552	Clca1	NM_009899	NM_009899
10502565	Clca2	NM_030601	NM_030601
10502638	Clca5	NM_178697	NM_178697
10438769	Cldn1	NM_016674	NM_016674
10417027	Cldn10	NM_021386	NM_021386
10422312	Cldn10	NM_023878	NM_023878
10602033	Cldn2	NM_016675	NM_016675
10534395	Cldn4	NM_009903	NM_009903
10440647	Cldn8	NM_018778	NM_018778
10541614	Clec4d	NM_010819	NM_010819
10547664	Clec4e	NM_019948	NM_019948
10436958	Clic6	NM_172469	NM_172469
10402473	Clmn	NM_053155	NM_053155
10417167	Clybl	NM_029556	NM_029556
10404132	Cmah	NM_001111110	NM_001111110
10545881	Cml2	NM_053096	NM_053096
10381154	Cnp	NM_009923	NM_009923
10381226	Coasy	NM_027896	NM_027896
10495449	Col11a1	NM_007729	NM_007729
10380419	Col1a1	NM_007742	NM_007742
10536220	Col1a2	NM_007743	NM_007743
10346015	Col3a1	NM_009930	NM_009930

10356520	Col6a3	AF064749	AF064749
10345101	Col9a1	NM_007740	NM_007740
10572378	Comp	NM_016685	NM_016685
10497451	Cpa3	NM_007753	NM_007753
10578904	Cpe	NM_013494	NM_013494
10361292	Cr2	NM_007758	NM_007758
10493108	Crabp2	NM_007759	NM_007759
10528102	Crot	NM_023733	NM_023733
10385912	Csf2	NM_009969	NM_009969
10425066	Csf2rb	NM_007780	NM_007780
10522827	Csn1s1	NM_007784	NM_007784
10522845	Csn1s2a	NM_007785	NM_007785
10362201	Ctgf	NM_010217	NM_010217
10409876	Ctla2a	NM_007796	NM_007796
10494262	Ctsk	NM_007802	NM_007802
10531415	Cxcl10	NM_021274	NM_021274
10541075	Cxcl12	NM_001012477	NM_001012477
10523359	Cxcl13	NM_018866	NM_018866
10409579	Cxcl14	NM_019568	NM_019568
10523156	Cxcl2	NM_009140	NM_009140
10523120	Cxcl5	NM_009141	NM_009141
10392056	Cyb561	NM_007805	NM_007805
10453057	Cyp1b1	NM_009994	NM_009994
10502655	Cyr61	NM_010516	NM_010516
10482802	Cytip	NM_139200	NM_139200
10368101	D10Bwg1379e	NM_001033258	NM_001033258
10418185	D14Ertd449e	BC031854	BC031854
10418198	D14Ertd449e	BC031854	BC031854
10418210	D14Ertd449e	BC031854	BC031854
10474825	D2Ertd750e	NM_026412	NM_026412
10538214	D330028D13Rik	BC055818	BC055818
10422728	Dab2	NM_023118	NM_023118
10553092	Dbp	NM_016974	NM_016974
10492136	Dclk1	NM_019978	NM_019978
10365974	Dcn	NM_007833	NM_007833
10496727	Ddah1	NM_026993	NM_026993
10362701	Ddo	NM_027442	NM_027442
10359929	Ddr2	NM_022563	NM_022563
10599696	Ddx26b	NM_172779	NM_172779
10571984	Ddx60	NM_001081215	NM_001081215
10497122	Depdc1a	NM_029523	NM_029523
10424126	Depdc6	NM_001037937	NM_001037937
10364093	Derl3	NM_024440	NM_024440
10400941	Dhrs7	NM_025522	NM_025522
10391207	Dhx58	NM_030150	NM_030150
10446596	Dlgap1	NM_177639	NM_177639
10448034	Dll1	NM_007865	NM_007865
10599120	Dock11	NM_001009947	NM_001009947
10596583	Dock3	NM_153413	NM_153413
10576235	Dpep1	NM_007876	NM_007876
10357300	Dpp10	NM_199021	NM_199021
10351293	Dpt	NM_019759	NM_019759
10495625	Dpyd	NM_170778	NM_170778
10458663	Dpysl3	NM_009468	NM_009468
10457686	Dsc2	NM_013505	NM_013505
10454113	Dsg1a	NM_010079	NM_010079
10404649	Dsp	NM_023842	NM_023842
10475456	Duox1	NM_001099297	NM_001099297
10486988	Duoxa1	NM_145395	NM_145395
10449284	Dusp1	NM_013642	NM_013642
10366043	Dusp6	NM_026268	NM_026268
10590690	Dync2h1	NM_029851	NM_029851
10413932	E130203B14Rik	NM_178791	NM_178791
10443367	E230001N04Rik	BC096647	BC096647
10410386	E430024C06Rik	AK149411	AK149411
10410388	E430024C06Rik	AK149411	AK149411
10375360	Ebf1	NM_007897	NM_007897
10362379	Echdc1	NM_025855	NM_025855
10500204	Ecm1	NM_007899	NM_007899
10404783	Edn1	NM_010104	NM_010104
10558936	Efcab4a	NM_001025103	NM_001025103
10374777	Efemp1	NM_146015	NM_146015
10415081	EG665955	FJ556972	FJ556972
10520121	EG665955	FJ556972	FJ556972
10578405	EG665955	FJ556972	FJ556972
10586076	EG665955	FJ556972	FJ556972
10563706	EG668725	DQ386867	DQ386867
10502105	Egf	NM_010113	NM_010113
10400304	Egln3	NM_028133	NM_028133
10454782	Egr1	NM_007913	NM_007913
10446777	Ehd3	NM_020578	NM_020578
10485445	Ehf	NM_007914	NM_007914
10474171	Elf5	NM_010125	NM_010125

10383953	Emid1	NM_080595	NM_080595
10542355	Emp1	NM_010128	NM_010128
10437639	Emp2	NM_007929	NM_007929
10368289	Enpp1	NM_008813	NM_008813
10428619	Enpp2	NM_015744	NM_015744
10368317	Enpp3	NM_134005	NM_134005
10445338	Enpp5	NM_032003	NM_032003
10458052	Epb4.1l4a	NM_013512	NM_013512
10503659	Epha7	NM_010141	NM_010141
10420935	Ephx2	NM_007940	NM_007940
10380896	Erbb2	NM_001003817	NM_001003817
10373467	Erbb3	NM_010153	NM_010153
10355278	Erbb4	NM_010154	NM_010154
10407281	Esm1	NM_023612	NM_023612
10511560	Esrp1	NM_194055	NM_194055
10479228	Etohi1	ENSMUST00000098999	ENSMUST00000098999
10395457	Etv1	NM_007960	NM_007960
10406934	Etv1	NM_007960	NM_007960
10363901	Etv5	NM_023794	NM_023794
10438626	Etv5	NM_023794	NM_023794
10371502	Fabp3	NM_010174	NM_010174
10508614	Fabp3	NM_010174	NM_010174
10497265	Fabp4	NM_024406	NM_024406
10465895	Fads2	NM_019699	NM_019699
10545045	Fam13a	NM_153574	NM_153574
10395136	Fam150b	NM_001159743	NM_001159743
10439276	Fam162a	NM_027342	NM_027342
10554156	Fam174b	NM_001162532	NM_001162532
10392464	Fam20a	NM_153782	NM_153782
10457780	Fam59a	NM_001033445	NM_001033445
10350506	Fam5c	NM_153539	NM_153539
10595070	Fam83b	BC120577	BC120577
10399465	Fam84a	NM_029007	NM_029007
10517967	Fblim1	NM_133754	NM_133754
10524941	Fbxo21	NM_145564	NM_145564
10434698	Fetub	NM_021564	NM_021564
10568436	Fgfr2	NM_010207	NM_010207
10492735	Fgg	NM_133862	NM_133862
10505994	Fggy	NM_001113412	NM_001113412
10505998	Fggy	NM_001113412	NM_001113412
10519983	Fgl2	NM_008013	NM_008013
10432243	Fkbp11	NM_024169	NM_024169
10449452	Fkbp5	NM_010220	NM_010220
10536845	Flnc	NM_001081185	NM_001081185
10397633	Flrt2	NM_201518	NM_201518
10359571	Fmo1	NM_010231	NM_010231
10355403	Fn1	NM_010233	NM_010233
10383556	Fn3krp	NM_181420	NM_181420
10447649	Fndc1	NM_001081416	NM_001081416
10397346	Fos	NM_010234	NM_010234
10560481	Fosb	NM_008036	NM_008036
10504534	Frmpd1	NM_001081172	NM_001081172
10527732	Fry	ENSMUST00000087204	ENSMUST00000087204
10505030	Fsd1l	NM_176966	NM_176966
10412260	Fst	NM_008046	NM_008046
10435641	Fstl1	NM_008047	NM_008047
10420846	Fzd3	NM_021458	NM_021458
10534426	Fzd9	NM_010246	NM_010246
10361246	G0s2	NM_008059	NM_008059
10385083	Gabrp	NM_146017	NM_146017
10364950	Gadd45b	NM_008655	NM_008655
10405211	Gadd45g	NM_011817	NM_011817
10483249	Galnt3	NM_015736	NM_015736
10531987	Gbp4	NM_008620	NM_008620
10496539	Gbp5	NM_153564	NM_153564
10580233	Gcdh	NM_008097	NM_008097
10466521	Gcnt1	NM_173442	NM_173442
10557470	Gdpd3	NM_024228	NM_024228
10503334	Gem	NM_010276	NM_010276
10375614	Gfpt2	NM_013529	NM_013529
10503508	Ggh	NM_010281	NM_010281
10503523	Ggh	NM_010281	NM_010281
10482059	Ggta1	NM_010283	NM_010283
10544588	Gimap3	NM_031247	NM_031247
10363173	Gja1	NM_010288	NM_010288
10601161	Gjb1	NM_008124	NM_008124
10420366	Gjb6	NM_001010937	NM_001010937
10350753	Glul	NM_008131	NM_008131
10374453	Glul	NM_008131	NM_008131
10433172	Glycam1	NM_008134	NM_008134
10412517	Gm10021	ENSMUST00000090647	ENSMUST00000090647
10566624	Gm10081	NM_001162940	NM_001162940
10479973	Gm10115	ENSMUST00000076071	ENSMUST00000076071

10379344	Gm10387	ENSMUST00000100735	ENSMUST00000100735
10601270	Gm10456	ENSMUST00000101339	ENSMUST00000101339
10516042	Gm10573	ENSMUST00000097904	ENSMUST00000097904
10501046	Gm10673	ENSMUST00000098753	ENSMUST00000098753
10494419	Gm10685	ENSMUST00000098840	ENSMUST00000098840
10545202	Gm1077	ENSMUST00000103355	ENSMUST00000103355
10425265	Gm10863	ENSMUST00000100455	ENSMUST00000100455
10545184	Gm10880	ENSMUST00000103344	ENSMUST00000103344
10487506	Gm14005	ENSMUST00000099395	ENSMUST00000099395
10545196	Gm1419	AY135151	AY135151
10479182	Gm14434	NM_001101804	NM_001101804
10479189	Gm14434	NM_001101804	NM_001101804
10479198	Gm14434	NM_001101804	NM_001101804
10490259	Gm14434	NM_001101804	NM_001101804
10490262	Gm14434	NM_001101804	NM_001101804
10490265	Gm14434	NM_001101804	NM_001101804
10490268	Gm14434	NM_001101804	NM_001101804
10490291	Gm14434	NM_001101804	NM_001101804
10545187	Gm1502	AJ416331	AJ416331
10352104	Gm16432	ENSMUST00000111149	ENSMUST00000111149
10352110	Gm16432	NM_001034899	NM_001034899
10476939	Gm4979	NM_001142411	NM_001142411
10428447	Gm5472	XM_484465	XM_484465
10538882	Gm5571 // Gm5571	ENSMUST00000103316 // ENSMUST00000103316	ENSMUST00000103316
10562657	Gm5595	NM_001008427	NM_001008427
10605522	Gm7173	NM_001099307	NM_001099307
10347915	Gm7609	NM_001081746	NM_001081746
10566583	Gm8995	AK172683	AK172683
10416705	Gm9264	XR_034445	XR_034445
10584252	Gm9513	EU703629	EU703629
10461856	Gna14	NM_008137	NM_008137
10512640	Gne	NM_015828	NM_015828
10363070	Gp49a	NM_008147	NM_008147
10604576	Gpc3	NM_016697	NM_016697
10426812	Gpd1	NM_010271	NM_010271
10538187	Gpnmb	NM_053110	NM_053110
10445251	Gpr110	NM_133776	NM_133776
10602896	Gpr64	NM_178712	NM_178712
10560237	Gpr77	NM_176912	NM_176912
10376201	Gpx3	NM_001083929	NM_001083929
10384398	Grb10	NM_010345	NM_010345
10547789	Grcc10	NM_013535	NM_013535
10394954	Grhl1	NM_001161406	NM_001161406
10577114	Grtp1	NM_025768	NM_025768
10471655	Gsn	NM_146120	NM_146120
10600814	Gspt2	NM_008179	NM_008179
10345065	Gsta3	NM_001077353	NM_001077353
10587315	Gsta4	NM_010357	NM_010357
10501235	Gstm4	NM_026764	NM_026764
10495243	Gstm5	NM_010360	NM_010360
10370000	Gstt1	NM_008185	NM_008185
10605571	Gyk	NM_212444	NM_212444
10485131	Gyltl1b	NM_172670	NM_172670
10450154	H2-Aa	NM_010378	NM_010378
10444291	H2-Ab1	NM_207105	NM_207105
10444298	H2-Eb1	NM_010382	NM_010382
10444814	H2-gs10	NM_001143689	NM_001143689
10444824	H2-Q6	NM_207648	NM_207648
10444821	H2-Q8	NM_023124	NM_023124
10450723	H2-T10	NM_010395	NM_010395
10450731	H2-t9	XR_034252	XR_034252
10562169	Hamp	NM_032541	NM_032541
10562166	Hamp2	NM_183257	NM_183257
10448117	Has1	NM_008215	NM_008215
10428707	Has2	NM_008216	NM_008216
10575095	Has3	NM_008217	NM_008217
10497203	Hey1	NM_010423	NM_010423
10598126	Hhipl2	BC034362	BC034362
10492860	Higd1a	NM_019814	NM_019814
10368527	Hint3	NM_025798	NM_025798
10404063	Hist1h2ab	NM_175660	NM_175660
10572897	Hmox1	NM_010442	NM_010442
10480459	Hnmt	NM_080462	NM_080462
10544768	Hoxa5	NM_010453	NM_010453
10562234	Hpn	NM_001110252	NM_001110252
10428081	Hrsp12	NM_008287	NM_008287
10529732	Hs3st1	NM_010474	NM_010474
10376956	Hs3st3a1	NM_178870	NM_178870
10386951	Hs3st3b1	NM_018805	NM_018805
10450367	Hspa1a	NM_010479	NM_010479
10450369	Hspa1a	NM_010479	NM_010479
10408928	Hspb1	NM_013560	NM_013560
10526410	Hspb1	NM_013560	NM_013560

10533050	Hspb8	NM_030704	NM_030704
10535904	Hsph1	NM_013559	NM_013559
10558150	Htra1	NM_019564	NM_019564
10436788	Hunk	NM_015755	NM_015755
10583519	Icam1	NM_010493	NM_010493
10346799	Icos	NM_017480	NM_017480
10355214	Idh1	NM_010497	NM_010497
10360398	Ifi202b	NM_008327	NM_008327
10360391	Ifi203	NM_001045481	NM_001045481
10360382	Ifi204	NM_008329	NM_008329
10360406	Ifi205	NM_172648	NM_172648
10502791	Ifi44	NM_133871	NM_133871
10462613	Ifit2	NM_008332	NM_008332
10442625	Igfals	NM_008340	NM_008340
10427125	Igfbp6	NM_008344	NM_008344
10403015	Igh	BC018535	BC018535
10403034	Igh	BC092065	BC092065
10403063	Igh	BC092065	BC092065
10403028	Igh	BC088837	BC088837
10403069	Igh-6	BC053409	BC053409
10403018	IghmAC38.205.12	ENSMUST00000103510	ENSMUST00000103510
10531126	Igj	NM_152839	NM_152839
10545173	Igk	BC128281	BC128281
10545247	Igk-V19-14	U59155	U59155
10545215	Igk-V28	DQ078272	DQ078272
10545198	Igkv4-71	ENSMUST00000103354	ENSMUST00000103354
10487597	Il1b	NM_008361	NM_008361
10345762	Il1r1	NM_008362	NM_008362
10345791	Il1rl1	NM_001025602	NM_001025602
10596027	Il20rb	NM_001033543	NM_001033543
10606016	Il2rg	NM_013563	NM_013563
10462442	Il33	NM_133775	NM_133775
10520452	Il6	NM_031168	NM_031168
10427628	Il7r	NM_008372	NM_008372
10544932	Inmt	NM_009349	NM_009349
10466932	Insl6	NM_013754	NM_013754
10404389	Irf4	NM_013674	NM_013674
10569102	Irf7	NM_016850	NM_016850
10416837	Irg1	NM_008392	NM_008392
10356084	Irs1	NM_010570	NM_010570
10433114	Itga5	NM_010577	NM_010577
10480090	Itga8	NM_001001309	NM_001001309
10469151	Itih5	NM_172471	NM_172471
10606369	Itm2a	NM_008409	NM_008409
10488060	Jag1	NM_013822	NM_013822
10442904	Jmjd8	NM_028101	NM_028101
10514576	Kank4	NM_172872	NM_172872
10576581	Kcnk1	NM_008430	NM_008430
10417798	Kcnk5	NM_021542	NM_021542
10550877	Kcnn4	NM_008433	NM_008433
10607403	Kctd12b	NM_175429	NM_175429
10462796	Kif11	NM_010615	NM_010615
10522530	Kit	NM_001122733	NM_001122733
10517727	Klhdc7a	NM_173427	NM_173427
10391052	Krt14	NM_016958	NM_016958
10391066	Krt17	NM_010663	NM_010663
10391036	Krt19	NM_008471	NM_008471
10390860	Krt23	NM_033373	NM_033373
10432785	Krt5	NM_027011	NM_027011
10432157	Lalba	NM_010679	NM_010679
10446763	Lbh	NM_029999	NM_029999
10478048	Lbp	NM_008489	NM_008489
10493907	Lce3f	NM_001018079	NM_001018079
10462035	Ldhb	NM_008492	NM_008492
10549097	Ldhb	NM_008492	NM_008492
10581865	Ldhd	NM_027570	NM_027570
10421853	Lect1	NM_010701	NM_010701
10438753	Leprel1	NM_173379	NM_173379
10393573	Lgals3bp	NM_011150	NM_011150
10363082	Lilrb4	NM_013532	NM_013532
10522217	Limch1	NM_001001980	NM_001001980
10438592	Liph	NM_001083894	NM_001083894
10403945	LOC100041230	BC139425	BC139425
10508800	LOC433762	AY140896	AY140896
10573865	LOC433762	AY140896	AY140896
10367744	LOC629446 // LOC629446	BC057932	BC057932
10573939	Lpcat2	NM_173014	NM_173014
10474526	Lpcat4	NM_207206	NM_207206
10522749	Lphn3	NM_198702	NM_198702
10572130	Lpl	NM_008509	NM_008509
10373223	Lrp1	NM_008512	NM_008512
10469984	Lrrc26	NM_146117	NM_146117
10559207	Lsp1	NM_019391	NM_019391

10401527	Ltbp2	NM_013589	NM_013589
10365983	Lum	NM_008524	NM_008524
10553537	Luzp2	NM_178705	NM_178705
10429564	Ly6a	NM_010738	NM_010738
10429568	Ly6c1	NM_010741	NM_010741
10429573	Ly6c2	NM_001099217	NM_001099217
10429520	Ly6d	NM_010742	NM_010742
10566934	Lyve1	NM_053247	NM_053247
10372652	Lyz1	NM_013590	NM_013590
10399202	Macc1	ENSMUST00000048880	ENSMUST00000048880
10607283	Maged2	NM_030700	NM_030700
10424113	Mal2	NM_178920	NM_178920
10369154	Man1a	NM_008548	NM_008548
10548729	Mansc1	NM_026345	NM_026345
10603746	Maob	NM_172778	NM_172778
10581645	Marveld3	NM_028584	NM_028584
10394990	Mboat2	NM_026037	NM_026037
10420247	Mcpt4	NM_010779	NM_010779
10510861	Megf6	NM_001162977	NM_001162977
10510878	Megf6	NM_001162977	NM_001162977
10541496	Mfap5	NM_015776	NM_015776
10434975	Mfi2	NM_013900	NM_013900
10382852	Mfsd11	NM_178620	NM_178620
10357660	Mfsd4	NM_001114662	NM_001114662
10397364	Mfsd7c	NM_145447	NM_145447
10539894	Mgll	NM_011844	NM_011844
10561187	Mia1	NM_019394	NM_019394
10532954	Mlec	NM_175403	NM_175403
10442458	Mlst8	NM_019988	NM_019988
10492355	Mme	NM_008604	NM_008604
10583044	Mmp13	NM_008607	NM_008607
10415052	Mmp14	NM_008608	NM_008608
10574350	Mmp15	NM_008609	NM_008609
10503448	Mmp16	NM_019724	NM_019724
10573924	Mmp2	NM_008610	NM_008610
10583071	Mmp3	NM_010809	NM_010809
10454353	Mocos	NM_026779	NM_026779
10360848	Mosc2	NM_133684	NM_133684
10584827	Mpzl2	NM_007962	NM_007962
10466172	Ms4a1	NM_007641	NM_007641
10461594	Ms4a4c	NM_029499	NM_029499
10461622	Ms4a6b	NM_027209	NM_027209
10574023	Mt2	NM_008630	NM_008630
10435112	Muc4	NM_080457	NM_080457
10513437	Mup2	NM_008647	NM_008647
10513455	Mup2	NM_008647	NM_008647
10513467	Mup2	NM_001045550	NM_001045550
10513472	Mup2	NM_008647	NM_008647
10513504	Mup2	NM_001045550	NM_001045550
10513420	Mup7	NM_001134675	NM_001134675
10437885	Myh11	NM_013607	NM_013607
10477920	Myl9	NM_172118	NM_172118
10565634	Myo7a	NM_008663	NM_008663
10497501	Naaladl2	XM_975226	XM_975226
10504743	Nans	NM_053179	NM_053179
10521731	Ncapg	NM_019438	NM_019438
10429140	Ndrg1	NM_008681	NM_008681
10493114	Nes	NM_016701	NM_016701
10409278	Nfil3	NM_017373	NM_017373
10498146	Nhlrc3	NM_172501	NM_172501
10428089	Nipal2	NM_145469	NM_145469
10574102	Nlrc5	FJ889356	FJ889356
10400157	Nova1	ENSMUST00000021438	ENSMUST00000021438
10502240	Npnt	NM_033525	NM_033525
10500780	Nr1h5	NM_198658	NM_198658
10572290	Nr2c2ap	NM_001025586	NM_001025586
10504838	Nr4a3	NM_015743	NM_015743
10587639	Nt5e	NM_011851	NM_011851
10606792	Nxf7	NM_130888	NM_130888
10533256	Oas1a	NM_145211	NM_145211
10533246	Oas1g	NM_011852	NM_011852
10522875	Odam	NM_027128	NM_027128
10353524	Ogfrl1	NM_001081079	NM_001081079
10405063	Ogn	NM_008760	NM_008760
10500808	Olfml3	NM_133859	NM_133859
10473481	Olfr1028	NM_001011774	NM_001011774
10473483	Olfr1029	NM_001011852	NM_001011852
10484569	Olfr1045	NM_147017	NM_147017
10484622	Olfr1085	NM_146590	NM_146590
10484839	Olfr1249	NM_001011796	NM_001011796
10484846	Olfr1252	NM_207568	NM_207568
10466268	Olfr1418	NM_001011524	NM_001011524
10434287	Olfr166	NM_147068	NM_147068

10440134	Olfr172	NM_147001	NM_147001
10471780	Olfr367	NM_001081010	NM_001081010
10583444	Olfr39	NM_146825	NM_146825
10566211	Olfr604	NM_147070	NM_147070
10591188	Olfr843	NM_146567	NM_146567
10479192	OTTMUSG00000016609	NM_001100416	NM_001100416
10479195	OTTMUSG00000016609	NM_001100416	NM_001100416
10525439	P2rx4	NM_011026	NM_011026
10601416	P2ry10	NM_172435	NM_172435
10509838	Padi2	NM_008812	NM_008812
10501734	Palmd	NM_023245	NM_023245
10474129	Pamr1	NM_173749	NM_173749
10397112	Papln	NM_130887	NM_130887
10496110	Papss1	NM_011863	NM_011863
10412345	Parp8	NM_001081009	NM_001081009
10416037	Pbk	NM_023209	NM_023209
10491885	Pcdh10	NM_001098171	NM_001098171
10498018	Pcdh18	NM_130448	NM_130448
10521972	Pcdh7	NM_018764	NM_018764
10455084	Pcdhb10	NM_053135	NM_053135
10455088	Pcdhb11	NM_053136	NM_053136
10455092	Pcdhb12	NM_053137	NM_053137
10455108	Pcdhb16	NM_053141	NM_053141
10455112	Pcdhb17	NM_053142	NM_053142
10455118	Pcdhb18	NM_053143	NM_053143
10455123	Pcdhb19	NM_053144	NM_053144
10455071	Pcdhb7	NM_053132	NM_053132
10455080	Pcdhb9	NM_053134	NM_053134
10556528	Pde3b	NM_011055	NM_011055
10443786	Pde9a	NM_008804	NM_008804
10583021	Pdgfd	NM_027924	NM_027924
10522503	Pdgfra	NM_011058	NM_011058
10472846	Pdk1	NM_172665	NM_172665
10543017	Pdk4	NM_013743	NM_013743
10518147	Pdpn	NM_010329	NM_010329
10427816	Pdzd2	NM_001081064	NM_001081064
10533085	Pebp1	NM_018858	NM_018858
10355464	Pecr	NM_023523	NM_023523
10511363	Penk	NM_001002927	NM_001002927
10564805	Pex11a	NM_011068	NM_011068
10480035	Pfkfb3	NM_133232	NM_133232
10370376	Pfkl	NM_008826	NM_008826
10498309	Pfn2	NM_019410	NM_019410
10550509	Pglyrp1	NM_009402	NM_009402
10506188	Pgm2	NM_028132	NM_028132
10442454	Pgp	NM_025954	NM_025954
10545528	Pigp	NM_001159616	NM_001159616
10349580	Pigr	NM_011082	NM_011082
10399924	Pik3cg	NM_020272	NM_020272
10507273	Pik3r3	NM_181585	NM_181585
10466779	Pip5k1b	NM_008846	NM_008846
10490794	Pkia	NM_008862	NM_008862
10413047	Plau	NM_008873	NM_008873
10476443	Plcb4	NM_013829	NM_013829
10565910	Plekhb1	NM_013746	NM_013746
10562576	Plekhf1	NM_024413	NM_024413
10557156	Plk1	NM_011121	NM_011121
10587799	Plscr2	NM_008880	NM_008880
10606495	Pof1b	NM_181579	NM_181579
10400649	Pole2	NM_011133	NM_011133
10542993	Pon3	NM_173006	NM_173006
10492021	Postn	NM_015784	NM_015784
10529977	Ppargc1a	NM_008904	NM_008904
10529979	Ppargc1a	NR_027710	NR_027710
10467206	Ppp1r3c	NM_016854	NM_016854
10358476	Prg4	NM_021400	NM_021400
10514779	Prkaa2	NM_178143	NM_178143
10399908	Prkar2b	NM_011158	NM_011158
10561335	Prkcz	NM_008860	NM_008860
10483856	Prkra	NM_011871	NM_011871
10423049	Prlr	BC096586	BC096586
10477717	Procr	NM_011171	NM_011171
10529824	Prom1	NM_008935	NM_008935
10487405	Prom2	NM_138750	NM_138750
10359624	Prrx1	NM_175686	NM_175686
10495316	Psrc1	NM_019976	NM_019976
10481518	Ptges	NM_022415	NM_022415
10397189	Ptgr2	NM_029880	NM_029880
10350516	Ptgs2	NM_011198	NM_011198
10494978	Ptpn22	NM_008979	NM_008979
10492448	Ptx3	NM_008987	NM_008987
10488797	Pxmp4	NM_021534	NM_021534
10393887	Pycr1	NM_144795	NM_144795

10351873	Pyhin1	NM_175026	NM_175026
10401114	Rab15	NM_134050	NM_134050
10499412	Rab25	NM_016899	NM_016899
10591643	Rab3d	NM_031874	NM_031874
10349724	Rab7l1	NM_144875	NM_144875
10509410	Rap1gap	NM_001081155	NM_001081155
10513922	Rasef	NM_001017427	NM_001017427
10486061	Rasgrp1	NM_011246	NM_011246
10372844	Rassf3	NM_138956	NM_138956
10366153	Rassf9	NM_146240	NM_146240
10467319	Rbp4	NM_001159487	NM_001159487
10384725	Rel	NM_009044	NM_009044
10436095	Retnla	NM_020509	NM_020509
10358408	Rgs1	NM_015811	NM_015811
10350733	Rgs16	NM_011267	NM_011267
10552156	Rhpn2	NM_027897	NM_027897
10602009	Rnf128	NM_023270	NM_023270
10381105	RP23-212C14.7	NM_001101613	NM_001101613
10543114	Rpa3	NM_026632	NM_026632
10503835	Rragd	NM_027491	NM_027491
10394978	Rrm2	NM_009104	NM_009104
10399710	Rsad2	NM_021384	NM_021384
10400926	Rtn1	NM_153457	NM_153457
10434778	Rtp4	NM_023386	NM_023386
10563611	Saa1	NM_009117	NM_009117
10553274	Saa2	NM_011314	NM_011314
10563597	Saa3	NM_011315	NM_011315
10416023	Scara5	NM_028903	NM_028903
10463355	Scd2	NM_009128	NM_009128
10483353	Scn7a	NM_009135	NM_009135
10557124	Scnn1b	NM_011325	NM_011325
10557111	Scnn1g	NM_011326	NM_011326
10571865	Scrg1	NM_009136	NM_009136
10350864	Sec16b	NM_033354	NM_033354
10401900	Sel1l	NM_001039089	NM_001039089
10351182	Sele	NM_011345	NM_011345
10373768	Selm	NM_053267	NM_053267
10351206	Selp	NM_011347	NM_011347
10519717	Sema3a	NM_009152	NM_009152
10585778	Sema7a	NM_011352	NM_011352
10408557	Serpinb1a	NM_025429	NM_025429
10349157	Serpinb2	NM_011111	NM_011111
10534667	Serpine1	NM_008871	NM_008871
10355984	Serpine2	NM_009255	NM_009255
10388430	Serpinf1	NM_011340	NM_011340
10484463	Serping1	NM_009776	NM_009776
10570957	Sfrp1	NM_013834	NM_013834
10492798	Sfrp2	NM_009144	NM_009144
10403834	Sfrp4	NM_016687	NM_016687
10375065	Sh3pxd2b	NM_177364	NM_177364
10395142	Sh3yl1	NM_013709	NM_013709
10487139	Shc4	NM_199022	NM_199022
10487000	Shf	NM_001013829	NM_001013829
10439583	Sidt1	NM_001159419	NM_001159419
10360173	Slamf7	NM_144539	NM_144539
10497296	Slc10a5	NM_001010834	NM_001010834
10495035	Slc16a1	NM_009196	NM_009196
10372988	Slc16a7	NM_011391	NM_011391
10387922	Slc25a11	NM_024211	NM_024211
10363563	Slc25a16	NM_175194	NM_175194
10517988	Slc25a34	NM_001013780	NM_001013780
10409713	Slc28a3	NM_022317	NM_022317
10451123	Slc29a1	NM_022880	NM_022880
10529671	Slc2a9	NM_001102414	NM_001102414
10505270	Slc31a2	NM_025286	NM_025286
10521892	Slc34a2	NM_011402	NM_011402
10511975	Slc35a1	NM_011895	NM_011895
10496324	Slc39a8	NM_001135149	NM_001135149
10354374	Slc40a1	NM_016917	NM_016917
10473384	Slc43a3	NM_021398	NM_021398
10501811	Slc44a3	NM_145394	NM_145394
10523021	Slc4a4	NM_018760	NM_018760
10521038	Slc5a1	NM_019810	NM_019810
10436945	Slc5a3	NM_017391	NM_017391
10365640	Slc5a8	NM_145423	NM_145423
10515115	Slc5a9	NM_145551	NM_145551
10599008	Slc6a14	NM_020049	NM_020049
10498024	Slc7a11	NM_011990	NM_011990
10571444	Slc7a2	NM_007514	NM_007514
10574985	Slc7a6	NM_178798	NM_178798
10549041	Slco1a5	NM_130861	NM_130861
10379615	Slfn5	NM_183201	NM_183201
10441902	Smoc2	NM_022315	NM_022315

10604844	Sms	NM_009214	NM_009214
10607524	Sms	NM_009214	NM_009214
10388185	Smtnl2	NM_177776	NM_177776
10461148	Snhg1	AK051045	AK051045
10564211	Snrpn	NM_013670	NM_013670
10428698	Sntb1	NM_016667	NM_016667
10594590	Snx1	NM_019727	NM_019727
10394674	Socs2	NM_007706	NM_007706
10571567	Sorbs2	NM_172752	NM_172752
10495285	Sort1	NM_019972	NM_019972
10549200	Sox5	NM_011444	NM_011444
10347948	Sp100	NM_013673	NM_013673
10379127	Spag5	NM_017407	NM_017407
10483401	Spc25	NM_025565	NM_025565
10523717	Spp1	NM_009263	NM_009263
10499899	Sprr1a	NM_009264	NM_009264
10386473	Srebf1	NM_011480	NM_011480
10369615	Srgn	NM_011157	NM_011157
10539080	St3gal5	NM_011375	NM_011375
10393166	St6galnac2	NM_009180	NM_009180
10471457	St6galnac4	NM_011373	NM_011373
10471464	St6galnac6	NM_016973	NM_016973
10480238	St8sia6	NM_145838	NM_145838
10390560	Stac2	NM_146028	NM_146028
10363181	Stard6	NM_029019	NM_029019
10456556	Stard6	NM_029019	NM_029019
10346168	Stat4	NM_011487	NM_011487
10519497	Steap4	NM_054098	NM_054098
10483381	Stk39	NM_016866	NM_016866
10578572	Stox2	NM_001114311	NM_001114311
10400143	Stxbp6	NM_144552	NM_144552
10441530	Sytl3	NM_031395	NM_031395
10496192	Tacr3	NM_021382	NM_021382
10375973	Taf13	NM_025444	NM_025444
10593123	Tagln	NM_011526	NM_011526
10372831	Tbc1d30	NM_029057	NM_029057
10408870	Tbc1d7	NM_025935	NM_025935
10354168	Tbc1d8	NM_018775	NM_018775
10402195	Tc2n	NM_028924	NM_028924
10478997	Tcfap2c	NM_009335	NM_009335
10494043	Tdrkh	NM_028307	NM_028307
10524955	Tesc	NM_021344	NM_021344
10401673	Tgfb3	NM_009368	NM_009368
10385533	Tgtp	NM_011579	NM_011579
10488378	Thbd	NM_009378	NM_009378
10447951	Thbs2	NM_011581	NM_011581
10393559	Timp2	NM_011594	NM_011594
10365482	Timp3	NM_011595	NM_011595
10492428	Tiparp	NM_178892	NM_178892
10578493	Tlr3	NM_126166	NM_126166
10595664	Tmed3	NM_025360	NM_025360
10504891	Tmeff1	NM_021436	NM_021436
10562096	Tmem147	NM_027215	NM_027215
10345869	Tmem182	NM_001081198	NM_001081198
10535174	Tmem184a	NM_001161548	NM_001161548
10465990	Tmem216	NM_026798	NM_026798
10400984	Tmem30b	NM_178715	NM_178715
10440918	Tmem50b	NM_030018	NM_030018
10501802	Tmem56	NM_178936	NM_178936
10517980	Tmem82	NM_145987	NM_145987
10441254	Tmprss2	NM_015775	NM_015775
10372177	Tmtc2	NM_177368	NM_177368
10513739	Tnc	NM_011607	NM_011607
10398665	Tnfaip2	NM_009396	NM_009396
10368144	Tnfaip3	NM_009397	NM_009397
10472050	Tnfaip6	NM_009398	NM_009398
10518300	Tnfrsf1b	NM_011610	NM_011610
10390748	Tns4	NM_172564	NM_172564
10444459	Tnxb	NM_031176	NM_031176
10512499	Tpm2	NM_009416	NM_009416
10448916	Tpsab1	NM_031187	NM_031187
10481949	Traf1	NM_009421	NM_009421
10362420	Trdn	NM_029726	NM_029726
10362440	Trdn	NM_029726	NM_029726
10362446	Trdn	NM_029726	NM_029726
10512766	Trim14	NM_029077	NM_029077
10462039	Trpm3	NM_001035244	NM_001035244
10536593	Tsen15	NM_025677	NM_025677
10515282	Tspan1	NM_133681	NM_133681
10543306	Tspan12	NM_173007	NM_173007
10369531	Tspan15	NM_197996	NM_197996
10606609	Tspan6	NM_019656	NM_019656
10366446	Tspan8	NM_146010	NM_146010

10514133	Ttc39b	NM_027238	NM_027238
10503502	Ttpa	NM_015767	NM_015767
10503520	Ttpa	NM_015767	NM_015767
10501963	Ugt8a	NM_011674	NM_011674
10397912	Unc79	NM_001081017	NM_001081017
10541307	Usp18	NM_011909	NM_011909
10367734	Ust	NM_177387	NM_177387
10546238	V1ra3	NM_053218	NM_053218
10408130	V1rh15	ENSMUST00000072369	ENSMUST00000072369
10408125	V1ri8	NM_145846	NM_145846
10541877	Vamp1	NM_001080557	NM_001080557
10501608	Vcam1	NM_011693	NM_011693
10384212	Wap	NM_011709	NM_011709
10394498	Wdr35	NM_172470	NM_172470
10587051	Wdr72	NM_001033500	NM_001033500
10409118	Wnk2	NM_029361	NM_029361
10385175	Wwc1	NM_170779	NM_170779
10605034	Xlr4c	NM_183094	NM_183094
10593225	Zbtb16	NM_001033324	NM_001033324
10571399	Zdhhc2	NM_178395	NM_178395
10453678	Zeb1	NM_011546	NM_011546
10442250	Zfp229	ENSMUST00000065871	ENSMUST00000065871
10541098	Zfp239	NM_001001792	NM_001001792
10415991	Zfp395	NM_199029	NM_199029
10416004	Zfp395	NM_199029	NM_199029
10488459	Zfp442	BC023805	BC023805
10591161	Zfp558	ENSMUST00000034647	ENSMUST00000034647
10594426	Zwilch	NM_026507	NM_026507
10499751	Creb3l4	NM_030080	NM_030080
10523151	Cxcl1	NM_008176	NM_008176
10537712	Gstk1	NM_029555	NM_029555
10580382	Neto2	NM_001081324	NM_001081324
10413710	Nt5dc2	NM_027289	NM_027289
10400844	Pygl	NM_133198	NM_133198

Table S7. Human genomic regions syntenic to the mouse regions identified in the backcross population. Note: Additional file 3 includes the list of supplementary references from this table.

tQTL	5' Marker (cM)	Marker Peak (cM)	3' Marker (cM)	Mouse Chr.	Human Homolog (±1 Mb interval)	Supplemental References	Candidate Genes	Supplemental References
**tQTL 1**	rs13476573<br>(49.41)	c2.loc87.5<br>(90.59)	rs13476913<br>(102.57)	2	20q11-q12	(1-4)	*ARHGAP40, DHX35, FAM83D, RALGAPB, SLC32A1 , TGM2*	(5-10)
**tQTL 2**	rs3724197<br>(4.68)<br>rs3724197<br>(4.68)	rs3724197<br>(4.68)<br>rs3724197<br>(4.68)	c7.loc60<br>(64.68)<br>c7.loc65<br>(69.68)	7	19q13; Xp22	(11-14)	*AURKC, XRCC1, TGFB1, CLCN4*	(15-19)
**tQTL 3**	c13.loc25<br>(27.19)<br>c13.loc25<br>(27.19)<br>c13.loc22.5<br>(24.69)	gnf13.057.501<br>(40.08)<br>gnf13.057.501<br>(40.08)<br>gnf13.057.501<br>(40.08)	c13.loc60<br>(62.19)<br>c13.loc62.5<br>(64.69)<br>c13.loc60<br>(62.19)	13	5q31; 9q21-22	(20-23)	*SPOCK1, MIR874, HNRNPA0, GOLM1, HNRNPK, NTRK2, RMI1*	(24-30)
**tQTL 4**	c18.loc10<br>(16.59)<br>c18.loc12.5<br>(19.09)	rs4231934<br>(55.31)<br>rs4231934<br>(55.31)	rs4231934<br>(55.31)<br>rs4231934<br>(55.31)	18	18q21	(31-33)	*MAPK4, SMAD4, SERPINB5, BCL2*	(34-38)
**tQTL 5**	rs6215262<br>(2.19)	c13.loc47.5<br>(49.69)	rs6316705<br>(70.93)	13	5q14	(12, 39)	*ARRDC3, CETN3, NR2F1, MIR2277, XRCC4*	(40-44)
**tQTL 6**	c4.loc7.5<br>(12.17)	rs13477617<br>(16.31)	rs13478069<br>(94.61)	4	6q16	(45-47)	*EPHA7, MAP3K7, CCNC, TSG1, CASP8AP2*	(48-52)
**tQTL 7**	c15.loc12.5<br>(14.46)<br>c15.loc12.5<br>(14.46)	rs6326790<br>(53.91)<br>c15.loc50<br>(51.96)	rs6326790<br>(53.91)<br>rs6326790<br>(53.91)	15	12p11; 22q13	(53-58)	*PIM3, MLC1, HDAC10, MAPK12, MAPK11*	(38, 59-62)
**tQTL 8**	rs3724197<br>(4.68)	rs3696018<br>(24.85)	rs6216320<br>(73.10)	7	19q13	(11, 12, 20)	*XRCC1, FGF21, BAX, CGB, RRAS, ATF5, NR1H2*	(16, 63-70)
**tQTL 9**	rs3703275<br>(16.29)<br>rs13459150<br>(18.10)	rs13459154<br>(43.01)<br>c17.loc25<br>(41.29)	c17.loc32.5<br>(48.79)<br>c17.loc35<br>(51.29)	17	5q21-q22	(71-74)	*LNPEP, FBXL17, FER*	(72,75-76)
**tQTL 10**	rs3672461<br>(4.34)	rs3698545<br>(68.30)	cel-14_116404928<br>(68.79)	14	13q31	(77-79)	*MIR17, MIR18A, MIR19A, MIR20A, MIR92A*	(80-84)
**tQTL 11**	rs3716790<br>(12.33)	rs13481230<br>(75.32)	rs13481230<br>(75.32)	11	17q24	(85-88)	*GNA13, AXIN2, WIPI1, MAP2K6*	(88-91)
**tQTL 12**	c5.loc2.5<br>(18.08)<br>rs6159963<br>(15.58)	c5.loc20<br>(35.58)<br>rs6256504<br>(23.20)	rs3662161<br>(60.53)<br>c5.loc35<br>(50.58)	5	4p14<br>4p16	(31,92)<br>(92, 97-99)	*KLB, RFC1, TLR6, TLR1, TLR10*<br>*STX18, MSX1, CRMP1, JAKMIP1*	(93-96)<br>(100-103)
**tQTL 13**	cel-x_67980997<br>(36.10)	rs13483994<br>(49.82)	gnfx.146.867<br>(73.95)	X	Xq21-q22	(104-106)	*RPA4, DIAPH2*	(107, 108)
**tQTL 14**	rs13475706<br>(2.04)	gnf01.037.906<br>(21.72)	c1.loc37.5<br>(39.54)	1	2q11-12	(109-111)	*NPAS2, MAP4K4, MFSD9, POU3F3*	(111-114)

Table S8. List of 354 mouse transcripts from the mouse signature also present in the human data from ERBB2 tumors described by Borg and colleagues (Staaf *et al.,* 2010), permitting the separation of three clusters with prognostic value represented in Figure S3A in Additional file 2. These genes are also used in Figure 3C. Green: upregulated genes. Red: downregulated genes.

Genes Present in Human data	Affymetrix Probe ID	Genes in Mouse Signature	mRNA Accession
A2M	10541354	A2m	NM_175628
ABCB6	10355742	Abcb6	NM_023732
ABI3BP	10436304	Abi3bp	NM_001014423
ACPP	10596303	Acpp	NM_019807
ACSS1	10488482	Acss1	NM_080575
ACTA2	10467124	Acta2	NM_007392
ADA	10489391	Ada	NM_007398
ADAMTS1	10440522	Adamts1	NM_009621
ADAMTS4	10351551	Adamts4	NM_172845
ADAMTS5	10440534	Adamts5	NM_011782
ADAMTS9	10546450	Adamts9	NM_175314
ADM	10556297	Adm	NM_009627
ADORA1	10357878	Adora1	NM_001008533
ADRB1	10464167	Adrb1	NM_007419
AEBP1	10374083	Aebp1	NM_009636
AGPS	10473024	Agps	NM_172666
ALCAM	10439895	Alcam	NM_009655
ALDH1A1	10461979	Aldh1a1	NM_013467
ALDH3B2	10460253	Aldh3b2	ENSMUST00000100032
ALDOC	10379153	Aldoc	NM_009657
ALPL	10517587	Alpl	NM_007431
ANGPTL4	10450038	Angptl4	NM_020581
ANPEP	10564818	Anpep	NM_008486
ANTXR1	10545974	Antxr1	NM_054041
APOC1	10560618	Apoc1	NM_007469
AQP1	10538459	Aqp1	NM_007472
AQP5	10426767	Aqp5	NM_009701
ARHGEF6	10604713	Arhgef6	NM_152801
ARRDC4	10564507	Arrdc4	NM_001042592
ATF3	10361091	Atf3	NM_007498
ATP2A3	10378216	Atp2a3	NM_016745
ATP2C2	10575926	Atp2c2	NM_026922
ATP6V1B1	10539449	Atp6v1b1	NM_134157
B4GALT6	10457733	B4galt6	NM_019737
BCKDHA	10561063	Bckdha	NM_007533
BGN	10600169	Bgn	NM_007542
BOC	10439612	Boc	NM_172506
BUB1	10487480	Bub1	NM_001113179
C1S	10547740	C1s	NM_144938
C3	10452316	C3	NM_009778
CADPS2	10543369	Cadps2	NM_153163
CAV1	10536499	Cav1	NM_007616
CAV2	10536494	Cav2	NM_016900
CBR4	10571978	Cbr4	NM_145595
CCL11	10379524	Ccl11	NM_011330
CCL2	10379511	Ccl2	NM_011333
CCNB1	10411739	Ccnb1	NM_172301
CCND1	10569646	Ccnd1	NM_007631
CCNJL	10375313	Ccnjl	NM_001045530
CCR7	10390763	Ccr7	NM_007719
CD2	10500677	Cd2	NM_013486
CD274	10462390	Cd274	NM_021893
CD53	10501063	Cd53	NM_007651
CD69	10548333	Cd69	NM_001033122
CD74	10456005	Cd74	NM_001042605
CDC14A	10501629	Cdc14a	NM_001080818
CDGAP	10439483	Cdgap	NM_020260
CDKN2C	10515090	Cdkn2c	NM_007671
CEL	10481278	Cel	NM_009885
CFB	10450325	Cfb	NM_008198
CFH	10358339	Cfh	NM_009888
CHIC1	10601303	Chic1	NM_009767
CHN2	10538356	Chn2	NM_023543

CHST11	10365290	Chst11	NM_021439
CITED4	10507726	Cited4	NM_019563
CLCA1	10502552	Clca1	NM_009899
CLDN10	10417027	Cldn10	NM_021386
CLDN4	10534395	Cldn4	NM_009903
CMAH	10404132	Cmah	NM_001111110
CNP	10381154	Cnp	NM_009923
COASY	10381226	Coasy	NM_027896
COL1A1	10380419	Col1a1	NM_007742
COL1A2	10536220	Col1a2	NM_007743
COL6A3	10356520	Col6a3	AF064749
COMP	10572378	Comp	NM_016685
CPE	10578904	Cpe	NM_013494
CRABP2	10493108	Crabp2	NM_007759
CREB3L4	10499751	Creb3l4	NM_030080
CROT	10528102	Crot	NM_023733
CSF2	10385912	Csf2	NM_009969
CSF2RB	10425066	Csf2rb	NM_007780
CSN1S1	10522827	Csn1s1	NM_007784
CTGF	10362201	Ctgf	NM_010217
CTSK	10494262	Ctsk	NM_007802
CXCL1	10523151	Cxcl1	NM_008176
CXCL12	10541075	Cxcl12	NM_001012477
CXCL14	10409579	Cxcl14	NM_019568
CYB561	10392056	Cyb561	NM_007805
CYP1B1	10453057	Cyp1b1	NM_009994
CYR61	10502655	Cyr61	NM_010516
DAB2	10422728	Dab2	NM_023118
DBP	10553092	Dbp	NM_016974
DCN	10365974	Dcn	NM_007833
DDAH1	10496727	Ddah1	NM_026993
DDR2	10359929	Ddr2	NM_022563
DEPDC6	10424126	Depdc6	NM_001037937
DHRS7	10400941	Dhrs7	NM_025522
DLGAP1	10446596	Dlgap1	NM_177639
DLL1	10448034	Dll1	NM_007865
DPYD	10495625	Dpyd	NM_170778
DPYSL3	10458663	Dpysl3	NM_009468
DSC2	10457686	Dsc2	NM_013505
DSP	10404649	Dsp	NM_023842
DUSP1	10449284	Dusp1	NM_013642
DUSP6	10366043	Dusp6	NM_026268
DYNC2H1	10590690	Dync2h1	NM_029851
ECHDC1	10362379	Echdc1	NM_025855
ECM1	10500204	Ecm1	NM_007899
EGR1	10454782	Egr1	NM_007913
EHD3	10446777	Ehd3	NM_020578
EMP1	10542355	Emp1	NM_010128
EMP2	10437639	Emp2	NM_007929
ENPP1	10368289	Enpp1	NM_008813
ENPP2	10428619	Enpp2	NM_015744
ENPP5	10445338	Enpp5	NM_032003
EPHX2	10420935	Ephx2	NM_007940
ERBB2	10380896	Erbb2	NM_001003817
ERBB3	10373467	Erbb3	NM_010153
ESM1	10407281	Esm1	NM_023612
ETV5	10363901	Etv5	NM_023794
FABP3	10371502	Fabp3	NM_010174
FABP4	10497265	Fabp4	NM_024406
FADS2	10465895	Fads2	NM_019699
FAM20A	10392464	Fam20a	NM_153782
FAM84A	10399465	Fam84a	NM_029007
FBLIM1	10517967	Fblim1	NM_133754
FBXO21	10524941	Fbxo21	NM_145564
FKBP11	10432243	Fkbp11	NM_024169
FKBP5	10449452	Fkbp5	NM_010220
FLNC	10536845	Flnc	NM_001081185
FN1	10355403	Fn1	NM_010233
FN3KRP	10383556	Fn3krp	NM_181420
FOS	10397346	Fos	NM_010234
FRMPD1	10504534	Frmpd1	NM_001081172
FSD1L	10505030	Fsd1l	NM_176966
FST	10412260	Fst	NM_008046

FSTL1	10435641	Fstl1	NM_008047
G0S2	10361246	G0s2	NM_008059
GADD45B	10364950	Gadd45b	NM_008655
GADD45G	10405211	Gadd45g	NM_011817
GCDH	10580233	Gcdh	NM_008097
GEM	10503334	Gem	NM_010276
GFPT2	10375614	Gfpt2	NM_013529
GGH	10503508	Ggh	NM_010281
GGTA1	10482059	Ggta1	NM_010283
GJA1	10363173	Gja1	NM_010288
GJB1	10601161	Gjb1	NM_008124
GLUL	10350753	Glul	NM_008131
GNE	10512640	Gne	NM_015828
GPC3	10604576	Gpc3	NM_016697
GPD1	10426812	Gpd1	NM_010271
GPNMB	10538187	Gpnmb	NM_053110
GPR77	10560237	Gpr77	NM_176912
GPX3	10376201	Gpx3	NM_001083929
GRB10	10384398	Grb10	NM_010345
GRHL1	10394954	Grhl1	NM_001161406
GRTP1	10577114	Grtp1	NM_025768
GSN	10471655	Gsn	NM_146120
GSPT2	10600814	Gspt2	NM_008179
GSTA4	10587315	Gsta4	NM_010357
GSTK1	10537712	Gstk1	NM_029555
GYLTL1B	10485131	Gyltl1b	NM_172670
HAMP	10562169	Hamp	NM_032541
HAS2	10428707	Has2	NM_008216
HEY1	10497203	Hey1	NM_010423
HMOX1	10572897	Hmox1	NM_010442
HNMT	10480459	Hnmt	NM_080462
HOXA5	10544768	Hoxa5	NM_010453
HPN	10562234	Hpn	NM_001110252
HRSP12	10428081	Hrsp12	NM_008287
HSPA1A	10450367	Hspa1a	NM_010479
HSPB1	10408928	Hspb1	NM_013560
HSPB8	10533050	Hspb8	NM_030704
HSPH1	10535904	Hsph1	NM_013559
HTRA1	10558150	Htra1	NM_019564
ICAM1	10583519	Icam1	NM_010493
IFI44	10502791	Ifi44	NM_133871
IGFALS	10442625	Igfals	NM_008340
IGFBP6	10427125	Igfbp6	NM_008344
IL2RG	10606016	Il2rg	NM_013563
INMT	10544932	Inmt	NM_009349
IRF4	10404389	Irf4	NM_013674
IRF7	10569102	Irf7	NM_016850
IRS1	10356084	Irs1	NM_010570
ITGA5	10433114	Itga5	NM_010577
ITM2A	10606369	Itm2a	NM_008409
JAG1	10488060	Jag1	NM_013822
KCNK1	10576581	Kcnk1	NM_008430
KCNN4	10550877	Kcnn4	NM_008433
KIF11	10462796	Kif11	NM_010615
KIT	10522530	Kit	NM_001122733
KLHDC7A	10517727	Klhdc7a	NM_173427
LBH	10446763	Lbh	NM_029999
LBP	10478048	Lbp	NM_008489
LEPREL1	10438753	Leprel1	NM_173379
LGALS3BP	10393573	Lgals3bp	NM_011150
LILRB4	10363082	Lilrb4	NM_013532
LRP1	10373223	Lrp1	NM_008512
LTBP2	10401527	Ltbp2	NM_013589
LUM	10365983	Lum	NM_008524
LY6D	10429520	Ly6d	NM_010742
MAGED2	10607283	Maged2	NM_030700
MAL2	10424113	Mal2	NM_178920
MANSC1	10548729	Mansc1	NM_026345
MAOB	10603746	Maob	NM_172778
MARVELD3	10581645	Marveld3	NM_028584
MEGF6	10510861	Megf6	NM_001162977
MFI2	10434975	Mfi2	NM_013900
MGLL	10539894	Mgll	NM_011844

MMP13	10583044	Mmp13	NM_008607
MMP14	10415052	Mmp14	NM_008608
MMP15	10574350	Mmp15	NM_008609
MMP2	10573924	Mmp2	NM_008610
MOCOS	10454353	Mocos	NM_026779
MYH11	10437885	Myh11	NM_013607
MYO7A	10565634	Myo7a	NM_008663
NANS	10504743	Nans	NM_053179
NDRG1	10429140	Ndrg1	NM_008681
NES	10493114	Nes	NM_016701
NETO2	10580382	Neto2	NM_001081324
NFIL3	10409278	Nfil3	NM_017373
NT5DC2	10413710	Nt5dc2	NM_027289
NT5E	10587639	Nt5e	NM_011851
OGFRL1	10353524	Ogfrl1	NM_001081079
OGN	10405063	Ogn	NM_008760
OLFML3	10500808	Olfml3	NM_133859
P2RX4	10525439	P2rx4	NM_011026
PAPSS1	10496110	Papss1	NM_011863
PBK	10416037	Pbk	NM_023209
PCDHB17	10455112	Pcdhb17	NM_053142
PDE3B	10556528	Pde3b	NM_011055
PDGFRA	10522503	Pdgfra	NM_011058
PDK1	10472846	Pdk1	NM_172665
PDK4	10543017	Pdk4	NM_013743
PDPN	10518147	Pdpn	NM_010329
PEBP1	10533085	Pebp1	NM_018858
PECR	10355464	Pecr	NM_023523
PEX11A	10564805	Pex11a	NM_011068
PFKFB3	10480035	Pfkfb3	NM_133232
PFKL	10370376	Pfkl	NM_008826
PFN2	10498309	Pfn2	NM_019410
PIGR	10349580	Pigr	NM_011082
PIK3CG	10399924	Pik3cg	NM_020272
PIK3R3	10507273	Pik3r3	NM_181585
PKIA	10490794	Pkia	NM_008862
PLAU	10413047	Plau	NM_008873
PLCB4	10476443	Plcb4	NM_013829
PLEKHB1	10565910	Plekhb1	NM_013746
PLEKHF1	10562576	Plekhf1	NM_024413
POLE2	10400649	Pole2	NM_011133
PON3	10542993	Pon3	NM_173006
POSTN	10492021	Postn	NM_015784
PPP1R3C	10467206	Ppp1r3c	NM_016854
PRKAR2B	10399908	Prkar2b	NM_011158
PRKCZ	10561335	Prkcz	NM_008860
PRKRA	10483856	Prkra	NM_011871
PRLR	10423049	Prlr	BC096586
PROCR	10477717	Procr	NM_011171
PRRX1	10359624	Prrx1	NM_175686
PSRC1	10495316	Psrc1	NM_019976
PTGES	10481518	Ptges	NM_022415
PTGS2	10350516	Ptgs2	NM_011198
PTPN22	10494978	Ptpn22	NM_008979
PTX3	10492448	Ptx3	NM_008987
PXMP4	10488797	Pxmp4	NM_021534
PYGL	10400844	Pygl	NM_133198
RAB15	10401114	Rab15	NM_134050
RAB3D	10591643	Rab3d	NM_031874
RAB7L1	10349724	Rab7l1	NM_144875
RBP4	10467319	Rbp4	NM_001159487
REL	10384725	Rel	NM_009044
RGS16	10350733	Rgs16	NM_011267
RHPN2	10552156	Rhpn2	NM_027897
RNF128	10602009	Rnf128	NM_023270
RPA3	10543114	Rpa3	NM_026632
RRM2	10394978	Rrm2	NM_009104
RTN1	10400926	Rtn1	NM_153457
SCARA5	10416023	Scara5	NM_028903
SCN7A	10483353	Scn7a	NM_009135
SEL1L	10401900	Sel1l	NM_001039089
SELE	10351182	Sele	NM_011345
SELM	10373768	Selm	NM_053267

SEMA7A	10585778	Sema7a	NM_011352
SERPINE1	10534667	Serpine1	NM_008871
SERPINE2	10355984	Serpine2	NM_009255
SERPINF1	10388430	Serpinf1	NM_011340
SERPING1	10484463	Serping1	NM_009776
SFRP1	10570957	Sfrp1	NM_013834
SFRP2	10492798	Sfrp2	NM_009144
SH3PXD2B	10375065	Sh3pxd2b	NM_177364
SH3YL1	10395142	Sh3yl1	NM_013709
SHF	10487000	Shf	NM_001013829
SIDT1	10439583	Sidt1	NM_001159419
SLAMF7	10360173	Slamf7	NM_144539
SLC16A1	10495035	Slc16a1	NM_009196
SLC16A7	10372988	Slc16a7	NM_011391
SLC25A11	10387922	Slc25a11	NM_024211
SLC29A1	10451123	Slc29a1	NM_022880
SLC2A9	10529671	Slc2a9	NM_001102414
SLC31A2	10505270	Slc31a2	NM_025286
SLC34A2	10521892	Slc34a2	NM_011402
SLC35A1	10511975	Slc35a1	NM_011895
SLC39A8	10496324	Slc39a8	NM_001135149
SLC40A1	10354374	Slc40a1	NM_016917
SLC43A3	10473384	Slc43a3	NM_021398
SLC6A14	10599008	Slc6a14	NM_020049
SLC7A11	10498024	Slc7a11	NM_011990
SLC7A2	10571444	Slc7a2	NM_007514
SLC7A6	10574985	Slc7a6	NM_178798
SNTB1	10428698	Sntb1	NM_016667
SNX1	10594590	Snx1	NM_019727
SORT1	10495285	Sort1	NM_019972
SOX5	10549200	Sox5	NM_011444
SP100	10347948	Sp100	NM_013673
SPAG5	10379127	Spag5	NM_017407
SPP1	10523717	Spp1	NM_009263
SREBF1	10386473	Srebf1	NM_011480
ST3GAL5	10539080	St3gal5	NM_011375
ST6GALNAC2	10393166	St6galnac2	NM_009180
ST6GALNAC4	10471457	St6galnac4	NM_011373
ST6GALNAC6	10471464	St6galnac6	NM_016973
STAC2	10390560	Stac2	NM_146028
STEAP4	10519497	Steap4	NM_054098
STK39	10483381	Stk39	NM_016866
TBC1D8	10354168	Tbc1d8	NM_018775
TDRKH	10494043	Tdrkh	NM_028307
TESC	10524955	Tesc	NM_021344
THBD	10488378	Thbd	NM_009378
THBS2	10447951	Thbs2	NM_011581
TIMP2	10393559	Timp2	NM_011594
TIMP3	10365482	Timp3	NM_011595
TIPARP	10492428	Tiparp	NM_178892
TMED3	10595664	Tmed3	NM_025360
TMEFF1	10504891	Tmeff1	NM_021436
TMEM30B	10400984	Tmem30b	NM_178715
TMEM50B	10440918	Tmem50b	NM_030018
TMEM56	10501802	Tmem56	NM_178936
TNC	10513739	Tnc	NM_011607
TNFAIP2	10398665	Tnfaip2	NM_009396
TNFAIP3	10368144	Tnfaip3	NM_009397
TNFRSF1B	10518300	Tnfrsf1b	NM_011610
TRAF1	10481949	Traf1	NM_009421
TRDN	10362420	Trdn	NM_029726
TRIM14	10512766	Trim14	NM_029077
TRPM3	10462039	Trpm3	NM_001035244
TSPAN1	10515282	Tspan1	NM_133681
TSPAN15	10369531	Tspan15	NM_197996
TSPAN6	10606609	Tspan6	NM_019656
TSPAN8	10366446	Tspan8	NM_146010
VAMP1	10541877	Vamp1	NM_001080557
WDR35	10394498	Wdr35	NM_172470
WNK2	10409118	Wnk2	NM_029361
ZBTB16	10593225	Zbtb16	NM_001033324
ZWILCH	10594426	Zwilch	NM_026507

Table S9. Some representative molecules from signaling pathways in tumors (red) and livers (green) correlated with different tumor pathophenotypes in the F1BX mice. n.s. indicates non-significant *P*-values. We schematize these data in Figure 4C.

Cell Signaling	Lifespan	Latency	Number of Tumors	Tumor Volume	Tumor Weight	Tumor Growth Rate	Tumor Growth Average Speed	Number of Metastasis
Tumor *ErbB2* RNA	n.s.	*r*=-0.238<br>*n*=101<br>*P*=0.0167	*r*=0.225<br>*n*=101<br>*P*=0.0234	*r*=0.213<br>*n*=99<br>*P*=0.0341	n.s.	n.s.	n.s.	*r*=0.233<br>*n*=98<br>*P*=0.021
Tumor ERK1/2	n.s.	n.s.	n.s.	n.s.	n.s.	n.s.	n.s.	n.s.
Tumor pERK1/2(T202/Y204)	*r*=0.458<br>*n*=107<br>*P*=<0.0001	*r*=0.412<br>*n*=107<br>*P*=<0.0001	n.s.	n.s.	n.s.	n.s.	n.s.	n.s.
Tumor pAKT(T308)	*r*=0.516<br>*n*=108<br>*P*=<0.0001	*r*=0.446<br>*n*=108<br>*P*=<0.0001	n.s.	n.s.	n.s.	n.s.	*r*=-0.271<br>*n*=105<br>*P*=0.0052	n.s.
Tumor AKT1	n.s.	n.s.	n.s.	n.s.	n.s.	n.s.	n.s.	n.s.
Tumor pAKT1(S473)	*r*=0.525<br>*n*=108<br>*P*=<0.0001	*r*=0.516<br>*n*=108<br>*P*=<0.0001	n.s.	n.s.	n.s.	n.s.	n.s.	n.s.
Tumor AKT2	n.s.	n.s.	n.s.	n.s.	n.s.	n.s.	n.s.	n.s.
Tumor pAKT2(S474)	*r*=0.22<br>*n*=104<br>*P*=0.025	*r*=0.194<br>*n*=104<br>*P*=0.0489	n.s.	n.s.	n.s.	n.s.	n.s.	n.s.
Tumor AKT3	n.s.	n.s.	n.s.	n.s.	n.s.	n.s.	n.s.	n.s.
Tumor pAKT3(S472)	*r*=0.489<br>*n*=105<br>*P*=<0.0001	*r*=0.503<br>*n*=105<br>*P*=<0.0001	n.s.	*r*=-0.276<br>*n*=102<br>*P*=0.0049	n.s.	n.s.	*r*=-0.264<br>*n*=102<br>*P*=0.0074	n.s.
Tumor mTOR	n.s.	n.s.	*r*=0.188<br>*n*=110<br>*P*=0.0494	n.s.	n.s.	n.s.	n.s.	n.s.
Tumor p-mTOR(S2481)	n.s.	n.s.	*r*=0.192<br>*n*=110<br>*P*=0.0439	n.s.	n.s.	*r*=-0.205<br>*n*=93<br>*P*=0.0488	n.s.	n.s.
Tumor p-mTOR(S2448)	n.s.	n.s.	n.s.	n.s.	*r*=-0.204<br>*n*=98<br>*P*=0.0436	n.s.	n.s.	n.s.
Liver ERK1/2	n.s.	n.s.	n.s.	n.s.	*r*=-0.241<br>*n*=101<br>*P*=0.0151	n.s.	n.s.	n.s.
Liver pERK1/2(T202/Y204)	*r*=0.478<br>*n*=120<br>*P*<0.0001	*r*=0.385<br>*n*=105<br>*P*<0.0001	*r*=-0.334<br>*n*=120<br>*P*=0.0002	n.s.	n.s.	n.s.	n.s.	n.s.
Liver pAKT(T308)	*r*=0.572<br>*n*=115<br>*P*<0.0001	*r*=0.532<br>*n*=102<br>*P*<0.0001	*r*=-0.245<br>*n*=115<br>*P*=0.0083	n.s.	n.s.	*r*=-0.22<br>*n*=91<br>*P*=0.0359	*r*=-0.212<br>*n*=101<br>*P*=0.0329	n.s.
Liver AKT1	*r*=0.573<br>*n*=116<br>*P*<0.0001	*r*=0.436<br>*n*=102<br>*P*<0.0001	*r*=-0.244<br>*n*=116<br>*P*=0.0083	n.s.	n.s.	n.s.	*r*=-0.207<br>*n*=101<br>*P*=0.0381	n.s.
Liver pAKT1(S473)	*r*=0.483<br>*n*=116<br>*P*<0.0001	*r*=0.444<br>*n*=102<br>*P*<0.0001	n.s.	*r*=-0.229<br>*n*=101<br>*P*=0.0212	n.s.	*r*=-0.223<br>*n*=91<br>*P*=0.0339	*r*=-0.275<br>*n*=101<br>*P*=0.0054	n.s.
Liver pAKT2(S474)	r=0.35<br>N=125<br>p<0.0001	r=0.294<br>N=109<br>p=0.0019	n.s.	n.s.	n.s.	n.s.	n.s.	n.s.
Liver AKT3	*r*=0.586<br>*n*=125<br>*P*<0.0001	*r*=0.479<br>*n*=109<br>*P*<0.0001	*r*=-0.399<br>*n*=125<br>*P*<0.0001	n.s.	n.s.	n.s.	*r*=-0.192<br>*n*=108<br>*P*=0.0461	n.s.
Liver pAKT3(S472)	*r*=0.437<br>*n*=125<br>*P*<0.0001	*r*=0.388<br>*n*=109<br>*P*<0.0001	*r*=-0.193<br>*n*=125<br>*P*=0.0312	n.s.	n.s.	n.s.	n.s.	n.s.
Liver mTOR	n.s.	n.s.	n.s.	n.s.	*r*=0.204<br>*n*=96<br>*P*=0.0457	n.s.	n.s.	n.s.
Liver p-mTOR(S2481)	n.s.	*r*=0.286<br>*n*=98<br>*P*=0.0043	n.s.	n.s.	n.s.	n.s.	n.s.	n.s.
Liver p-mTOR(S2448)	n.s.	n.s.	n.s.	n.s.	n.s.	n.s.	n.s.	n.s.

Table S10. Level of different components of signaling pathways in tumors (red) and livers (green) in each different prognosis cluster. Upper values: mean and SEM. Lower values: median and IR; n.s. indicates non-significant *P*-values; n/a, not applicable in mice without tumors. These values are represented in Figure 4E and in Figures S5C, S5D, and S5E in Additional file 2. (*) ANOVA; (#) Tukey´s post-test.

Tumor Signaling	Cluster 1<br>Bad<br>*n* =25	Cluster 2<br>Middle-Bad<br>*n* =59	Cluster 3<br>Middle-Good<br>*n* =26	Cluster 4<br>Good<br>*n* =14	Cluster 5<br>Excellent<br>*n* =23	*P* -value (*)	Pairwise Comparison	Post-test<br>*P* -value (#)
Tumor *ErbB2* RNA	0.787±0.114<br>0.694 (0.544)	0.85±0.062<br>0.74 (0.581)	0.546±0.064<br>0.451 (0.353)	0.457±0.076<br>0.38 (0.273)	n/a	0.003	Cluster 2 Cluster 3<br>Cluster 2 Cluster 4	0.0198<br>0.0211
Tumor ERBB2	1.423±0.215<br>1.44 (0.87)	1.743±0.097<br>1.627 (0.595)	1.497±0.101<br>1.5 (0.761)	1.623±0.217<br>1.48 (1.156)	n/a	n.s.		
Tumor pERBB2	1.887±0.312<br>1.858 (1.343)	2.64±0.148<br>2.598 (1.629)	2.576±0.164<br>2.667 (1.35)	2.643±0.375<br>2.38 (1.982)	n/a	n.s.		
Tumor ERK	3.472±0.057<br>3.352 (0.452)	3.539±0.032<br>3.531 (0.319)	3.511±0.043<br>3.466 (0.375)	3.504±0.047<br>3.551 (0.324)	n/a	n.s.		
Tumor pERK(T202/Y204)	1.191±0.151<br>1.044 (0.723)	1.5±0.092<br>1.334 (0.783)	1.737±0.152<br>1.893 (0.963)	1.901±0.192<br>1.808 (1.024)	n/a	0.021	Cluster 1 Cluster 4	0.0277
Tumor AKT1	0.224±0.012<br>0.229 (0.061)	0.246±0.014<br>0.222 (0.128)	0.211±0.018<br>0.191 (0.081)	0.229±0.02<br>0.208 (0.1)	n/a	n.s.		
Tumor pAKT(T308)	0.198±0.019<br>0.167 (0.094)	0.307±0.02<br>0.257 (0.162)	0.322±0.029<br>0.293 (0.167)	0.445±0.046<br>0.454 (0.335)	n/a	0.0002	Cluster 1 Cluster 4<br>Cluster 2 Cluster 4<br>Cluster 1 Cluster 2<br>Cluster 1 Cluster 3	<0.0001<br>0.0096<br>0.0499<br>0.0470
Tumor pAKT1(S473)	0.617±0.058<br>0.583 (0.365)	0.683±0.034<br>0.643 (0.322)	0.831±0.077<br>0.798 (0.493)	1.078±0.082<br>1.079 (0.486)	n/a	<0.0001	Cluster 1 Cluster 4<br>Cluster 2 Cluster 4	0.0003<br><0.0001
Tumor AKT2	3.27±0.057<br>3.165 (0.433)	3.295±0.028<br>3.276 (0.247)	3.277±0.05<br>3.259 (0.284)	3.234±0.031<br>3.267 (0.127)	n/a	n.s.		
Tumor pAKT2(S474)	0.549±0.054<br>0.505 (0.373)	0.722±0.045<br>0.699 (0.405)	0.666±0.07<br>0.579 (0.533)	0.818±0.079<br>0.791 (0.513)	n/a	n.s.		
Tumor AKT3	1.747±0.155<br>1.611 (1.325)	1.753±0.066<br>1.694 (0.647)	1.832±0.096<br>1.837 (0.414)	1.689±0.165<br>1.524 (1.184)	n/a	n.s.		
Tumor pAKT3(S472)	0.612±0.096<br>0.514 (0.378)	0.809±0.061<br>0.695 (0.426)	0.953±0.103<br>0.899 (0.701)	1.231±0.162<br>1.233 (0.985)	n/a	0.004	Cluster 1 Cluster 4<br>Cluster 2 Cluster 4	0.0033<br>0.0222
Tumor mTOR	2.833±0.073<br>2.844 (0.306)	2.958±0.046<br>3.023 (0.456)	2.936±0.059<br>2.972 (0.353)	2.735±0.109<br>2.793 (0.309)	n/a	n.s.		
Tumor p-mTOR(S2481)	0.646±0.07<br>0.575 (0.286)	0.866±0.056<br>0.796 (0.601)	0.89±0.069<br>0.817 (0.418)	0.679±0.058<br>0.677 (0.229)	n/a	n.s.		
Tumor p-mTOR(S2448)	1.107±0.07<br>1.157 (0.39)	1.351±0.069<br>1.278 (0.665)	1.316±0.099<br>1.219 (0.658)	1.11±0.07<br>1.162 (0.343)	n/a	n.s.		
Liver ERK1/2	1.7518±0.1568<br>1.5595 (0.659)	1.8118±0.068<br>1.709 (0.6195)	1.723±0.0926<br>1.693 (0.6925)	1.9597±0.1325<br>1.7335 (0.7773)	1.9649±0.114<br>1.927 (0.5448)	n.s.		
Liver pERK1/2(T202/Y204)	0.2876±0.0284<br>0.295 (0.1293)	0.3269±0.0179<br>0.292 (0.093)	0.3337±0.028<br>0.304 (0.222)	0.4651±0.0325<br>0.494 (0.175)	0.5011±0.042<br>0.489 (0.312)	< 0.0001	Cluster 1 Cluster 4<br>Cluster 1 Cluster 5<br>Cluster 2 Cluster 4<br>Cluster 2 Cluster 5<br>Cluster 3 Cluster 4<br>Cluster 3 Cluster 5	0.0065<br>0.0003<br>0.0087<br>0.0002<br>0.0404<br>0.0021
Liver AKT1	0.1047±0.0116<br>0.094 (0.0438)	0.1528±0.0098<br>0.128 (0.086)	0.1247±0.0115<br>0.122 (0.067)	0.265±0.0152<br>0.2695 (0.061)	0.2322±0.0256<br>0.227 (0.189)	< 0.0001	Cluster 1 Cluster 4<br>Cluster 1 Cluster 5<br>Cluster 2 Cluster 4<br>Cluster 2 Cluster 5<br>Cluster 3 Cluster 4<br>Cluster 3 Cluster 5	< 0.0001<br>< 0.0001<br>< 0.0001<br>0.0016<br>< 0.0001<br>0.0001
Liver AKT(T308)	0.1159±0.0113<br>0.1135 (0.052)	0.1617±0.0084<br>0.144 (0.061)	0.1525±0.0109<br>0.15 (0.0465)	0.2269±0.0138<br>0.222 (0.0638)	0.23±0.0233<br>0.219 (0.1405)	< 0.0001	Cluster 1 Cluster 5<br>Cluster 1 Cluster 4<br>Cluster 2 Cluster 5<br>Cluster 2 Cluster 4<br>Cluster 3 Cluster 5<br>Cluster 3 Cluster 4	< 0.0001<br>< 0.0001<br>0.0029<br>0.0075<br>0.0033<br>0.0071
Liver pAKT1(S473)	0.1041±0.0153<br>0.0975 (0.0648)	0.173±0.0118<br>0.15 (0.094)	0.1488±0.0123<br>0.139 (0.0825)	0.2338±0.021<br>0.2105 (0.107)	0.2471±0.0303<br>0.218 (0.134)	< 0.0001	Cluster 1 Cluster 5<br>Cluster 1 Cluster 4<br>Cluster 1 Cluster 2<br>Cluster 2 Cluster 5<br>Cluster 3 Cluster 5<br>Cluster 3 Cluster 4	0.0001<br>0.001<br>0.046<br>0.0258<br>0.0065<br>0.0396
Liver pAKT2(S474)	0.2299±0.0335<br>0.198 (0.105)	0.2978±0.019<br>0.273 (0.137)	0.2852±0.0197<br>0.285 (0.122)	0.3267±0.0276<br>0.3085 (0.1253)	0.3896±0.0378<br>0.422 (0.2393)	0.0167	Cluster 1 Cluster 5	0.0083
Liver AKT3	0.1782±0.0169<br>0.1615 (0.086)	0.2368±0.0156<br>0.208 (0.156)	0.2055±0.0239<br>0.1965 (0.1788)	0.4028±0.0419<br>0.3545 (0.1573)	0.4734±0.0481<br>0.487 (0.2868)	< 0.0001	Cluster 1 Cluster 5<br>Cluster 1 Cluster 4<br>Cluster 2 Cluster 5<br>Cluster 2 Cluster 4<br>Cluster 3 Cluster 5<br>Cluster 3 Cluster 4	< 0.0001<br>0.0001<br>< 0.0001<br>0.0003<br>< 0.0001<br>0.0001
Liver pAKT3(S472)	0.2972±0.0374<br>0.2305 (0.2525)	0.4514±0.034<br>0.388 (0.2755)	0.3707±0.0456<br>0.3035 (0.221)	0.7019±0.0904<br>0.64 (0.4778)	0.7407±0.117<br>0.597 (0.7483)	< 0.0001	Cluster 1 Cluster 5<br>Cluster 1 Cluster 4<br>Cluster 2 Cluster 5<br>Cluster 2 Cluster 4<br>Cluster 3 Cluster 5<br>Cluster 3 Cluster 4	0.0004<br>0.0027<br>0.0047<br>0.0327<br>0.001<br>0.0072
Liver mTOR	1.9249±0.2549<br>2.21 (1.666)	1.7142±0.1273<br>1.8735 (1.7273)	2.1813±0.1864<br>2.505 (0.9333)	1.7541±0.2575<br>1.888 (1.5755)	1.5388±0.2252<br>1.379 (1.5025)	n.s.		
Liver p-mTOR(S2481)	0.3222±0.0843<br>0.204 (0.5365)	0.2426±0.0345<br>0.107 (0.3825)	0.44±0.0575<br>0.483 (0.4565)	0.2467±0.0652<br>0.112 (0.375)	0.2123±0.0642<br>0.1185 (0.198)	0.0339	Cluster 2 Cluster 3	0.0275
Liver p-mTOR(S2448)	0.4685±0.0859<br>0.548 (0.586)	0.38±0.0436<br>0.282 (0.521)	0.5895±0.0674<br>0.673 (0.416)	0.3208±0.067<br>0.231 (0.4095)	0.2849±0.0647<br>0.1985 (0.4473)	0.0211	Cluster 3 Cluster 5	0.0274

Table S11. Comparison between levels of some representative proteins from signaling pathways in tumors from the FVB and F1 genetic backgrounds.

Blue: statistical trend. The data show the medians and the interquartile ranges. (*) Wilcoxon´s test. We represent these data in Figure S8 in Additional file 2.

Tumor Signaling	F1	FVB	*P*-value (*)
**ERK1/2**	3.42 (3.33-3.54) *n*=10	3.34 (3.23-3.45) *n*=8	n.s.
**pERK1/2(T202/Y204)**	1.78 (1.62-2.21) *n*=10	2.09 (1.4-2.71) *n*=9	n.s.
**pAKT(T308)**	0.51 (0.31-0.55) *n*=9	0.21 (0.17-0.29) *n*=7	*P*=0.0036
**AKT1**	0.15 (0.13-0.17) *n*=9	0.13 (0.12-0.14) *n*=7	n.s.
**pAKT1(S473)**	0.76 (0.58-1.13) *n*=10	0.48 (0.27-0.79) *n*=7	n.s.
**AKT2**	2.91 (2.74-3.10) *n*=10	3.13 (2.90-3.27) *n*=9	*P*=0.0724
**pAKT2(S474)**	0.31 (0.22-0.37) *n*=10	0.39 (0.32-0.55) *n*=9	*P*=0.0723
**AKT3**	1.63 (1.36-1.62) *n*=10	1.57 (1.31-1.72) *n*=9	n.s.
**pAKT3(S472)**	0.62 (0.51-0.81) *n*=9	0.56 (0.37-0.96) *n*=9	n.s.
**mTOR**	2.95 (2.25-3.14) *n*=9	3.45 (3.41-3.47) *n*=6	*P*=0.0014
**p-mTOR(S2448)**	0.88 (0.64-1.14) *n*=9	1.69 (1.34-2.04) *n*=7	*P*=0.0300
**p-mTOR(S2481)**	0.69 (0.50-0.91) *n*=9	1.17 (0.88-1.79) *n*=7	*P*=0.0172
***ErbB2* RNA**	0.49 (0.35-0.67) *n*=16	0.74 (0.41-1.14) *n*=18	*P*=0.0384
**ERBB2**	1.50 (1.30-1.69) *n*=6	1.76 (1.68-1.99) *n*=7	*P*=0.0223
**pERBB2**	1.70 (1.57-1.87) *n*=6	1.94 (1.79-2.09) *n*=7	*P*=0.0633

Table S12. Genomic regions associated with variable levels of some representative molecules from signaling pathways in the F1BX tumors. tsQTL, tumor-signaling QTL. These data are schematized in Figure 6.

Tumor Signaling	Locus	Chr.	Marker Peak	Confidence Interval (0.05)	Location (cM)	LOD Score	Average ± SEM FF	Average ± SEM FB	Higher Effect Allele
				c4.loc50	54.67	1.67			
ErbB2 RNA	tsQTL1	4	rs6386918		81.69	2.50	0.88 ± 0.06	0.60 ± 0.05	FVB
				c4.loc80	84.67	1.72			
				cel-x_39961894	23.24	1.08			
ERK1/2	tsQTL2	X	rs13483761		26.50	1.53	3.48 ± 0.02	3.61 ± 0.04	C57BL/6
				rs13484031	55.21	0.13			
				c3.loc35	38.49	0.82			
pERK1/2(T202/Y204)	tsQTL3	3	c3.loc62.5		65.99	1.84	1.66 ± 0.07	1.20 ± 0.14	FVB
				c3.loc75	78.49	0.97			
				rs13475706	2.04	0.29			
pAKT(T308)	tsQTL4	1	rs13475909		34.50	1.52	0.36 ± 0.02	0.28 ± 0.02	FVB
				rs3677638	74.70	0.02			
				rs3686936	14.35	0.31			
	tsQTL5	9	rs6152427		69.46	1.92	0.84 ± 0.04	0.65 ± 0.05	FVB
				rs6152427	69.46	1.92			
				c11.loc12.5	24.83	0.24			
	tsQTL6	11	rs3722590		55.57	1.56	0.72 ± 0.04	0.79 ± 0.04	C57BL/6
				rs13481230	75.32	0.34			
				c12.loc7.5	12.83	0.44			
	tsQTL7	12	rs3707048		33.69	1.73	0.66 ± 0.05	0.83 ± 0.04	C57BL/6
				rs13481657	61.84	0.13			
				cel-9_29909656	18.83	1.23			
pAKT1(S473)	tsQTL5	9	c9.loc55		67.15	2.14	0.84 ± 0.04	0.65 ± 0.05	FVB
				rs6152427	69.46	2.05			
				c12.loc7.5	12.83	0.44			
	tsQTL7	12	rs3707048		33.69	1.73	0.66 ± 0.05	0.83 ± 0.04	C57BL/6
				rs13481657	61.84	0.13			
				c18.loc2.5	9.09	0.69			
AKT2	tsQTL8	18	c18.loc30		36.59	1.51	3.22 ± 0.03	3.33 ± 0.03	C57BL/6
				rs6320743	46.85	0.75			
				c9.loc30	42.15	0.56			
pAKT2(S474)	tsQTL5	9	c9.loc50		62.15	1.63	0.77 ± 0.04	0.61 ± 0.04	FVB
				rs6152427	69.46	1.51			
				rs3709002	5.33	1.55			
	tsQTL9	12	rs3709002		5.33	1.55	0.60 ± 0.05	0.77 ± 0.04	C57BL/6
				rs13481657	61.84	0.74			
				rs3703275	16.29	1.89			
	tsQTL10	17	rs3703275		16.29	1.89	0.61 ± 0.04	0.79 ± 0.04	C57BL/6
				c17.loc20	36.29	0.84			
				rs6215262	2.19	1.21			
AKT3	tsQTL11	13	rs13481839		40.56	1.84	1.90 ± 0.07	1.61 ± 0.07	FVB
				c13.loc62.5	64.69	1.00			
				rs3709002	5.33	1.55			
pAKT3(S472)	tsQTL9	12	rs3709002		5.33	1.55	0.70 ± 0.07	0.98 ± 0.06	C57BL/6
				rs13481573	42.59	0.09			
				rs3699833	3.12	0.24			
mTOR	tsQTL12	6	cel-6_57082524		31.74	1.67	2.99 ± 0.04	2.82 ± 0.04	FVB
				c6.loc62.5	65.62	0.19			
				c6.loc2.5	5.62	0.24			
p-mTOR(S2448)	tsQTL12	6	rs6268125		42.86	1.66	1.41 ± 0.06	1.15 ± 0.06	FVB
				c6.loc55	58.12	0.32			

Table S13. Comparison between levels of different proteins from signaling pathways in liver, spleen and kidney from C57BL/6, FVB, F1 and F1BX genetic background mice. Blue, *P*-values with statistical trend; n/a, not applicable. (*) Kruskal-Wallis test ; (#) Dunn Method for Join Ranking post-test. Some of these values are represented in Figure S8 in Additional file 2.

Organ	Signaling Protein	C57	F1	FVB	*P*-value (*)	Pairwise Comparisons (#)		
						C57-F1	C57-FVB	F1-FVB
LIVER	ERK1/2	1.4340 (1.344-1.527) *n*=3	1.7070 (1.374-2.054) *n*=5	1.3700 (1.318-1.544) *n*=4	n.s.			
	pERK1/2(T202/Y204)	0.2920 (0.270-0.307) *n*=5	0.2545 (0.239-0.264) *n*=4	0.1920 (0.176-0.257) *n*=4	*P*=0.0268	n.s.	*P*=0.0440	n.s.
	pAKT(T308)	0.1360 (0.119-0.190) *n*=9	0.1670 (0.136-0.175) *n*=10	0.1295 (0.116-0.145) *n*=10	*P*=0.0684	n.s.	n.s.	*P*=0.0668
	AKT1	0.1232 (0.109-0.140) *n*=10	0.1455 (0.123-0.188) *n*=10	0.1165 (0.091-0.135) *n*=10	*P*=0.0585	n.s.	n.s.	*P*=0.0665
	pAKT1(S473)	0.1280 (0.115-0.168) *n*=10	0.1280 (0.112-0.134) *n*=9	0.0955 (0.080-0.134) *n*=10	*P*=0.0558	n.s.	*P*=0.0603	n.s.
	AKT2	1.0435 (0.896-1.140) *n*=10	0.8730 (0.744-1.103) *n*=9	1.2160 (0.853-1.357) *n*=9	n.s.			
	pAKT2(S474)	0.1105 (0.098-0.126) *n*=10	0.1455 (0.101-0.171) *n*=10	0.1230 (0.106-0.148) *n*=9	n.s.			
	AKT3	0.1030 (0.085-0.133) *n*=10	0.0995 (0.08-0.102) *n*=8	0.0945 (0.079-0.102) *n*=8	n.s.			
	pAKT3(S472)	0.0555 (0.033-0.096) *n*=10	0.058 (0.038-0.063) *n*=8	0.055 (0.036-0.078) *n*=7	n.s.			
	mTOR	1.6860 (1.64-2.438) *n*=5	2.6390 (2.070-2.827) *n*=5	2.6590 (2.275-2.896) *n*=5	*P*=0.0627	n.s.	n.s.	n.s.
	p-mTOR(S2448)	0.3330 (0.257-0.6155) *n*=5	0.6310 (0.540-0.791) *n*=5	0.7930 (0.625-1.185) *n*=5	*P*=0.0539	n.s.	*P*=0.0589	n.s.
	p-mTOR(S2481)	0.2630 (0.196-0.602) *n*=5	0.5970 (0.469-0.797) *n*=5	0.6110 (0.388-0.749) *n*=5	n.s.			
KIDNEY	pAKT(T308)	0.3430 (0.2955-0.37) *n*=9	0.2340 (0.1895-0.2795) *n*=9	0.1880 (0.1775-0.236) *n*=9	*P*=0.0013	n.s.	*P*=0.0010	n.s.
	AKT1	0.2460 (0.2125-0.289) *n*=9	0.1740 (0.13-0.2455) *n*=10	0.1560 (0.128-0.1725) *n*=9	*P*=0.0079	n.s.	*P*=0.0062	n.s.
	pAKT1(S473)	0.2710 (0.226-0.3165) *n*=9	0.1600 (0.119-0.1995) *n*=8	0.1415 (0.1225-0.171) *n*=8	*P*=0.0019	*P*=0.0243	*P*=0.0033	n.s.
SPLEEN	pAKT(T308)	0.7740 (0.518-1.115) *n*=10	0.4440 (0.355-0.587) *n*=10	0.3810 (0.263-0.517) *n*=7	*P*=0.0077	n.s.	*P*=0.0085	n.s.
	AKT1	0.7105 (0.432-1.089) *n*=10	0.4585 (0.286-0.537) *n*=10	0.3440 (0.241-0.474) *n*=10	*P*=0.0130	n.s.	*P*=0.0114	n.s.
	pAKT1(S473)	0.9095 (0.532-1.391) *n*=10	0.4360 (0.348-0.649) *n*=10	0.4020 (0.287-0.546) *n*=10	*P*=0.0082	*P*=0.0925	*P*=0.0089	n.s.

Table S14. Genomic regions associated with variable levels of some proteins from signaling pathways in the F1BX livers. LsQTL, Liver-signaling QTL. These data are schematized in Figure 6.

Liver Signaling	Locus	Chr.	Marker Peak	Confidence Interval (0.05)	Location (cM)	LOD Score	Average ± SEM FF	Average ± SEM FB	Higher Effect Allele
**ERK1/2**	**LsQTL-1**	4	rs3653593	c4.loc5	9.67	1.66	1.70 ± 0.06	1.99 ± 0.07	C57BL/6
					14.27	2.30			
				c4.loc67.5	72.17	1.32			
**pERK1/2(T202/Y204)**	**LsQTL-2**	3	rs3724545	c3.loc10	13.49	0.01	0.35 ± 0.01	0.53 ± 0.06	C57BL/6
					27.98	1.61			
				rs3724562	85.72	0.07			
**pAKT(T308)**	**LsQTL-3**	11	rs3698446	c11.loc42.5	54.83	0.93	0.15 ± 0.01	0.19 ± 0.01	C57BL/6
					70.75	2.39			
				c11.loc62.5	74.83	1.43			
**AKT1**	**LsQTL-3**	11	rs3698446	c11.loc42.5	54.83	2.33	0.14 ± 0.01	0.19 ± 0.01	C57BL/6
					70.75	3.22			
				c11.loc62.5	74.83	2.35			
**pAKT1(S473)**	**LsQTL-3**	11	rs3698446	c11.loc42.5	54.83	1.44	0.15 ± 0.01	0.20 ± 0.01	C57BL/6
					70.75	2.04			
				c11.loc62.5	74.83	1.37			
**pAKT2(S474)**	**LsQTL-4**	3	c3.loc57.5	c3.loc10	13.49	0.15	0.32 ± 0.01	0.24 ± 0.03	FVB
					60.99	1.57			
				rs3724562	85.72	0.54			
	**LsQTL-5**	15	rs6326790	c15.loc12.5	14.46	0.69	0.33 ± 0.02	0.27 ± 0.02	FVB
					53.91	1.58			
				rs6326790	53.91	1.58			
**mTOR**	**LsQTL-6**	7	rs6279696	rs3720735	10.39	1.07	1.44 ± 0.14	2.01 ± 0.10	C57BL/6
					44.34	2.33			
				rs3708100	53.83	1.00			
**p-mTOR(S2448)**	**LsQTL-6**	7	rs6279696	rs3720735	10.39	0.65	0.28 ± 0.05	0.48 ± 0.03	C57BL/6
					44.34	2.44			
				rs3708100	53.83	1.27			

Table S15. Pairwise associations between levels of some representative molecules from cell signaling pathways in ERBB2 tumors from F1BX mice. n.s., non-significant *P*-values.

Tumor Signaling	ERBB2	pERBB2	ERK1/2	pERK1/2 (T202/Y204)	pAKT(T308)	AKT1	pAKT1(S473)	AKT2	pAKT2(S474)	AKT3	pAKT3(S472)	mTOR	p-mTOR(S2481)	p-mTOR(S2448)
**ErbB2 RNA**	n.s.	n.s.	n.s.	n.s.	n.s.	n.s.	*r* = -0.258<br>*n* = 97<br>*P* = 0.0107	n.s.	n.s.	n.s.	*r* = -0.221<br>*n* = 94<br>*P* = 0.032	n.s.	n.s.	n.s.
**ERBB2**		*r* = 0.77<br>*n* = 108<br>*P* < 0.0001	n.s.	n.s.	*r* = -0.265<br>*n* = 106<br>*P* = 0.0061	n.s.	*r* = -0.210<br>*n* = 106<br>*P* = 0.0307	*r* = -0.217<br>*n* = 101<br>*P* = 0.0294	*r* = -0.233<br>*n* = 102<br>*P* = 0.0185	*r* = -0.247<br>*n* = 103<br>*P* = 0.0118	*r* = -0.232<br>*n* = 103<br>*P* = 0.0185	n.s.	n.s.	n.s.
**pERBB2**			n.s.	n.s.	*r* = -0.299<br>*n* = 106<br>*P* = 0.0019	n.s.	n.s.	n.s.	n.s.	n.s.	n.s.	n.s.	n.s.	n.s.
**ERK1/2**				n.s.	n.s.	n.s.	n.s.	*r* = 0.277<br>*n* = 99<br>*P* = 0.0054	n.s.	n.s.	n.s.	*r* = 0.209<br>*n* = 103<br>*P* = 0.0343	n.s.	*r* = 0.359<br>*n* = 103<br>*P* = 0.0002
**pERK1/2(T202/Y204)**					*r* = 0.353<br>*n* = 105<br>*P* = 0.0002	n.s.	*r* = 0.523<br>*n* = 104<br>*P* < 0.0001	n.s.	*r* = 0.442<br>*n* = 104<br>*P* < 0.0001	*r* = 0.297<br>*n* = 105<br>*P* = 0.0021	*r* = 0.461<br>*n* = 105<br>*P* < 0.0001	n.s.	n.s.	*r* = 0.22<br>*n* = 107<br>*P* = 0.0231
**pAKT(T308)**						n.s.	*r*=0.563<br>*n*=107<br>*P* < 0.0001	n.s.	*r* = 0.359<br>*n* = 103<br>*P* = 0.0002	*r* = 0.333<br>*n* = 103<br>*P* = 0.0006	*r* = 0.624<br>*n* = 103<br>*P* < 0.0001	n.s.	*r* = 0.237<br>*n* = 108<br>*P* = 0.0136	n.s.
**AKT1**							n.s.	n.s.	n.s.	n.s.	n.s.	n.s.	n.s.	n.s.
**pAKT1(S473)**								n.s.	*r* = 0.358<br>*n* = 102<br>*P* = 0.0002	*r* = 0.268<br>*n* = 103<br>*P* = 0.0063	*r* = 0.706<br>*n* = 102<br>*P* < 0.0001	n.s.	n.s.	n.s.
**AKT2**									*r* = 0.33<br>*n* = 102<br>*P* = 0.0007	n.s.	n.s.	*r* = 0.296<br>*n* = 102<br>*P* = 0.0025	n.s.	*r* = 0.268<br>*n* = 102<br>*P* = 0.0064
**pAKT2(S474)**										*r* = 0.274<br>*n* = 102<br>*P* = 0.0053	*r* = 0.514<br>*n* = 102<br>*P* < 0.0001	n.s.	*r* = 0.241<br>*n* = 104<br>*P* = 0.0136	*r* = 0.268<br>*n* = 104<br>*P* = 0.0059
**AKT3**											*r* = 0.599<br>*n* = 103<br>*P* < 0.0001	*r* = 0.199<br>*n* = 105<br>*P* = 0.0414	n.s.	n.s.
**pAKT3(S472)**												n.s.	n.s.	n.s.
**mTOR**													*r* = 0.35<br>*n* = 110<br>*P* = 0.0002	*r* = 0.716<br>*n* = 110<br>*P* < 0.0001
**p-mTOR(S2481)**														*r* = 0.511<br>*n* = 110<br>*P* < 0.0001

Table S16. Pairwise associations between levels of some molecules from cell signaling pathways in mouse livers from F1BX. n.s., non-significant *P* values.

Liver Signaling	ERK1/2	pERK1/2/(T202/Y204)	pAKT(T308)	AKT1	pAKT1(S473)	pAKT2(S474)	AKT3	pAKT3(S472)	mTOR	p-mTOR(S2481)	p-mTOR(S2448)
**ERK1/2**		n.s.	n.s.	n.s.	n.s.	$r = 0.186$<br>$n = 124$<br>$P = 0.0387$	$r = 0.238$<br>$n = 124$<br>$P = 0.0079$	n.s.	n.s.	n.s.	$r = -0.242$<br>$n = 119$<br>$P = 0.008$
**pERK1/2/(T202/Y204)**			$r = 0.58$<br>$n = 110$<br>$P < 0.0001$	$r = 0.638$<br>$n = 111$<br>$P < 0.0001$	$r = 0.464$<br>$n = 111$<br>$P < 0.0001$	$r = 0.639$<br>$n = 119$<br>$P < 0.0001$	$r = 0.78$<br>$n = 120$<br>$P < 0.0001$	$r = 0.698$<br>$n = 120$<br>$P < 0.0001$	n.s.	n.s.	n.s.
**pAKT(308)**				$r = 0.847$<br>$n = 115$<br>$P < 0.0001$	$r = 0.896$<br>$n = 115$<br>$P < 0.0001$	$r = 0.48$<br>$n = 114$<br>$P < 0.0001$	$r = 0.68$<br>$n = 115$<br>$P < 0.0001$	$r = 0.65$<br>$n = 115$<br>$P < 0.0001$	n.s.	n.s.	n.s.
**AKT1**					$r = 0.757$<br>$n = 116$<br>$P < 0.0001$	$r = 0.56$<br>$n = 115$<br>$P < 0.0001$	$r = 0.72$<br>$n = 116$<br>$P < 0.0001$	$r = 0.683$<br>$n = 116$<br>$P < 0.0001$	n.s.	n.s.	$r = -0.233$<br>$n = 115$<br>$P = 0.0121$
**pAKT1(S473)**						$r = 0.382$<br>$n = 115$<br>$P < 0.0001$	$r = 0.626$<br>$n = 116$<br>$P < 0.0001$	$r = 0.563$<br>$n = 116$<br>$P < 0.0001$	n.s.	n.s.	n.s.
**pAKT2(S474)**							$r = 0.628$<br>$n = 124$<br>$P < 0.0001$	$r = 0.658$<br>$n = 124$<br>$P < 0.0001$	n.s.	n.s.	n.s.
**AKT3**								$r = 0.847$<br>$n = 125$<br>$P < 0.0001$	n.s.	n.s.	$r = -0.21$<br>$n = 119$<br>$P = 0.0219$
**pAKT3(S472)**									n.s.	n.s.	n.s.
**mTOR**										$r = 0.859$<br>$n = 111$<br>$P < 0.0001$	$r = 0.864$<br>$n = 119$<br>$P < 0.0001$
**p-mTOR(S2481)**											$r = 0.89$<br>$n = 112$<br>$P < 0.0001$

Table S17. Pairwise correlations between levels of some molecules from cell signaling pathways in ERBB2 tumors and livers from F1BX mice. n.s., non-significant *P*-values.

		LIVER								
		**pERK1/2 (T202/Y204)**	**pAKT (T308)**	**AKT1**	**pAKT1 (S473)**	**pAKT2 (S474)**	**AKT3**	**pAKT3 (S472)**	**p-mTOR (S2481)**	**p-mTOR (S2448)**
TUMOR	**pERK1/2(T202/Y204)**	n.s.	n.s.	n.s.	n.s.	n.s.	n.s.	n.s.	r=0.316 n=87 P=0.0029	r=0.241 n=93 P=0.0201
	**pAKT(T308)**	r=0.316 n=96 P=0.0017	r=0.434 n=94 P<0.0001	r=0.417 n=94 P<0.0001	r=0.325 n=94 P=0.0014	r=0.224 n=100 P=0.0254	r=0.248 n=100 P=0.0129	r=0.216 n=100 P=0.0306	n.s.	n.s.
	**pAKT1(S473)**	r=0.307 n=96 P=0.0023	r=0.359 n=93 P=0.0004	r=0.337 n=93 P=0.001	r=0.237 n=93 P=0.0223	r=0.252 n=100 P=0.0115	r=0.281 n=100 P=0.0047	n.s.	n.s.	n.s.
	**pAKT2(S474)**	n.s.	n.s.	r=0.236 n=89 P=0.0261	n.s.	r=0.244 n=96 P=0.0165	n.s.	n.s.	n.s.	n.s.
	**pAKT3(S472)**	r=0.262 n=92 P=0.0116	r=0.419 n=90 P<0.0001	r=0.404 n=90 P<0.0001	r=0.321 n=90 P=0.002	r=0.281 n=97 P=0.0052	r=0.233 n=96 P=0.0225	r=0.231 n=96 P=0.0236	n.s.	n.s.

Table S18. Subphenotypes related to levels of different serum metabolites associated with tumor pathophenotypes obtained by mass spectrometry. n.s. indicates non-significant *P*-values. These data are represented in Figure S7 in Additional file 2. We include the supplementary references from this table in Additional file 3.

Serum Metabolites	Lifespan	Latency	Duration of Disease	Number of Tumors	Tumor Volume	Tumor Weight	Tumor Growth Rate	Tumor Growth Average Speed	Number of Metastasis	Supplemental References
2,3-Dihydroxybutanoic acid	n.s.	n.s.	n.s.	n.s.	n.s.	n.s.	*r* =0.22<br>*n* =83<br>*P* =0.0459	n.s.	n.s.	
2-Hydroxybutanoic acid	*r* =0.23<br>*n* =112<br>*P* =0.0148	n.s.	n.s.	n.s.	n.s.	n.s.	n.s.	n.s.	n.s.	(115-117)
2-Monopalmitin	n.s.	*r* =0.211<br>*n* =102<br>*P* =0.0329	*r* =-0.251<br>*n* =102<br>*P* =0.0109	n.s.	n.s.	*r* =-0.254<br>*n* =85<br>*P* =0.0188	n.s.	n.s.	n.s.	(118)
4-Hydroxyphenylacetic acid	n.s.	n.s.	n.s.	n.s.	n.s.	n.s.	*r* =0.231<br>*n* =83<br>*P* =0.0355	n.s.	n.s.	(119-121)
Alanine	n.s.	n.s.	n.s.	n.s.	n.s.	n.s.	n.s.	n.s.	*r* =-0.264<br>*n* =95<br>*P* =0.0099	(122, 123)
Allantoic acid	n.s.	n.s.	n.s.	n.s.	n.s.	n.s.	n.s.	*r* =-0.205<br>*n* =98<br>*P* =0.0431	n.s.	
Asparagine	n.s.	n.s.	n.s.	n.s.	n.s.	n.s.	n.s.	n.s.	*r* =-0.213<br>*n* =95<br>*P* =0.0386	(124-127)
β-Mannosylglycerate	*r* =0.192<br>*n* =112<br>*P* =0.0429	n.s.	n.s.	n.s.	n.s.	n.s.	n.s.	n.s.	n.s.	
Citric acid	n.s.	*r* =0.207<br>*n* =102<br>*P* =0.037	n.s.	n.s.	n.s.	*r* =-0.311<br>*n* =85<br>*P* =0.0038	n.s.	n.s.	*r* =-0.301<br>*n* =95<br>*P* =0.003	(115, 128, 129)
Creatinine	*r* =-0.187<br>*n* =112<br>*P* =0.0481	n.s.	n.s.	n.s.	n.s.	n.s.	n.s.	n.s.	n.s.	(125)
Cystine	n.s.	n.s.	*r* =-0.356<br>*n* =102<br>*P* =0.0002	n.s.	*r* =-0.218<br>*n* =98<br>*P* =0.0313	n.s.	n.s.	n.s.	n.s.	(130-132)
Cytidine	n.s.	*r* =-0.223<br>*n* =102<br>*P* =0.0243	n.s.	n.s.	n.s.	n.s.	n.s.	n.s.	n.s.	(133-135)
Erythritol	n.s.	n.s.	n.s.	n.s.	n.s.	n.s.	n.s.	n.s.	*r* =-0.243<br>*n* =95<br>*P* =0.0176	(136, 137)
Fructose	n.s.	n.s.	n.s.	n.s.	n.s.	n.s.	n.s.	n.s.	*r* =-0.219<br>*n* =95<br>*P* =0.0327	(138, 140)
Fumaric acid	n.s.	*r* =0.248<br>*n* =102<br>*P* =0.0121	*r* =-0.221<br>*n* =102<br>*P* =0.0257	n.s.	n.s.	n.s.	n.s.	n.s.	n.s.	(141-144)
Galactonic acid	n.s.	n.s.	n.s.	n.s.	*r* =-0.229<br>*n* =98<br>*P* =0.0233	*r* =-0.219<br>*n* =85<br>*P* =0.0439	n.s.	*r* =-0.233<br>*n* =98<br>*P* =0.0209	n.s.	
Glucuronic acid	n.s.	n.s.	*r* =-0.22<br>*n* =102<br>*P* =0.0261	n.s.	n.s.	n.s.	n.s.	n.s.	n.s.	(145-149)
Glutamine	n.s.	n.s.	n.s.	n.s.	n.s.	n.s.	n.s.	n.s.	*r* =-0.308<br>*n* =95<br>*P* =0.0024	(150-154)
Glycerol	n.s.	n.s.	n.s.	n.s.	n.s.	n.s.	n.s.	n.s.	*r* =-0.295<br>*n* =95<br>*P* =0.0037	(155-158)
Glycolic acid	n.s.	n.s.	n.s.	n.s.	n.s.	n.s.	n.s.	*r* =0.201<br>*n* =98<br>*P* =0.0477	n.s.	(159, 160)
Hippuric acid	n.s.	n.s.	n.s.	n.s.	n.s.	n.s.	n.s.	n.s.	*r* =-0.288<br>*n* =95<br>*P* =0.0046	(136, 161-164)
Hydrocinnamic acid	n.s.	n.s.	n.s.	n.s.	n.s.	n.s.	n.s.	n.s.	*r* =-0.203<br>*n* =95<br>*P* =0.0484	(137)
Hydroxylamine	n.s.	n.s.	*r* =-0.291<br>*n* =102<br>*P* =0.003	*r* =-0.206<br>*n* =112<br>*P* =0.0291	n.s.	n.s.	n.s.	n.s.	n.s.	(115)
Icosenoic acid	n.s.	n.s.	*r* =-0.208<br>*n* =102<br>*P* =0.036	n.s.	n.s.	*r* =-0.281<br>*n* =85<br>*P* =0.0091	n.s.	n.s.	n.s.	(165)
Inositol-4-monophosphate	n.s.	n.s.	n.s.	*r* =-0.354<br>*n* =112<br>*P* =0.0001	n.s.	n.s.	n.s.	n.s.	n.s.	(166, 167)

Serum Metabolites	Lifespan	Latency	Duration of Disease	Number of Tumors	Tumor Volume	Tumor Weight	Tumor Growth Rate	Tumor Growth Average Speed	Number of Metastasis	Supplemental References
Lactic acid	*r* =-0.205<br>*n* =112<br>*P* =0.0298	*r* =-0.198<br>*n* =102<br>*P* =0.0459	n.s.	n.s.	n.s.	n.s.	n.s.	n.s.	n.s.	(168-172)
Lauric acid	n.s.	n.s.	n.s.	*r* =-0.187<br>*n* =112<br>*P* =0.0478	n.s.	n.s.	n.s.	n.s.	n.s.	(173-176)
Linoleic acid	n.s.	n.s.	n.s.	n.s.	n.s.	n.s.	n.s.	n.s.	*r* =-0.252<br>*n* =95<br>*P* =0.0138	(176-181)
Linolenic acid	n.s.	n.s.	n.s.	n.s.	n.s.	n.s.	*r* =0.226<br>*n* =83<br>*P* =0.0403	n.s.	n.s.	(182-185)
Malic acid	n.s.	*r* =0.207<br>*n* =102<br>*P* =0.0369	*r* =-0.249<br>*n* =102<br>*P* =0.0118	n.s.	n.s.	*r* =-0.239<br>*n* =85<br>*P* =0.0276	n.s.	n.s.	n.s.	(121, 186)
Maltose	n.s.	n.s.	n.s.	n.s.	n.s.	n.s.	*r* =0.261<br>*n* =83<br>*P* =0.0172	n.s.	n.s.	(187, 188)
Methylhexadecanoic acid	n.s.	n.s.	n.s.	n.s.	n.s.	n.s.	n.s.	n.s.	*r* =-0.21<br>*n* =95<br>*P* =0.0413	(189)
Monopalmitin-1-glyceride	n.s.	n.s.	n.s.	n.s.	n.s.	n.s.	*r* =0.234<br>*n* =83<br>*P* =0.0333	n.s.	n.s.	(190)
Myristic acid	n.s.	n.s.	n.s.	n.s.	n.s.	n.s.	n.s.	n.s.	*r* =-0.223<br>*n* =95<br>*P* =0.0298	(174, 175)
N-Acetyl-D-mannosamine	*r* =-0.252<br>*n* =112<br>*P* =0.0073	*r* =-0.257<br>*n* =102<br>*P* =0.0091	n.s.	n.s.	n.s.	n.s.	n.s.	n.s.	n.s.	(191)
Octadecanol	n.s.	n.s.	n.s.	*r* =-0.232<br>*n* =112<br>*P* =0.0137	n.s.	n.s.	n.s.	n.s.	n.s.	
Oleic acid	n.s.	n.s.	n.s.	n.s.	n.s.	n.s.	n.s.	n.s.	*r* =-0.293<br>*n* =95<br>*P* =0.004	(173, 192-194)
Palmitic acid	n.s.	n.s.	n.s.	n.s.	n.s.	n.s.	n.s.	n.s.	*r* = -0.201<br>*n* =95<br>*P* =0.0505	(195-198)
Pelargonic acid	*r* =0.193<br>*n* =112<br>*P* =0.0411	n.s.	n.s.	*r* =-0.273<br>*n* =112<br>*P* =0.0036	n.s.	n.s.	n.s.	n.s.	n.s.	(199)
Phosphoethanolamine	n.s.	n.s.	n.s.	n.s.	n.s.	*r* =0.267<br>*n* =85<br>*P* =0.0137	*r* =0.226<br>*n* =83<br>*P* =0.0398	n.s.	n.s.	(200-202)
Pseudo-uridine	n.s.	n.s.	*r* =-0.296<br>*n* =102<br>*P* =0.0025	n.s.	*r* =-0.223<br>*n* =98<br>*P* =0.0274	n.s.	*r* =0.22<br>*n* =83<br>*P* =0.0454	n.s.	n.s.	(203, 204)
Serine	n.s.	n.s.	n.s.	n.s.	n.s.	n.s.	n.s.	n.s.	*r* =-0.216<br>*n* =95<br>*P* =0.0357	(205)
Sorbitol	n.s.	n.s.	n.s.	n.s.	n.s.	n.s.	n.s.	n.s.	*r* =-0.297<br>*n* =95<br>*P* =0.0035	(206, 207)
Sorbose	n.s.	n.s.	n.s.	n.s.	n.s.	n.s.	n.s.	n.s.	*r* =-0.214<br>*n* =95<br>*P* =0.0376	(208)
Succinic acid	n.s.	n.s.	*r* =-0.248<br>*n* =102<br>*P* =0.0121	n.s.	n.s.	n.s.	n.s.	n.s.	n.s.	(209)
Taurine	n.s.	n.s.	n.s.	n.s.	n.s.	n.s.	n.s.	n.s.	*r* =-0.211<br>*n* =95<br>*P* =0.0404	(210)
Thymidine	n.s.	n.s.	n.s.	n.s.	n.s.	n.s.	n.s.	n.s.	n.s.	(211, 212)
Tryptophan	n.s.	n.s.	n.s.	n.s.	*r* =0.213<br>*n* =98<br>*P* =0.0353	*r* =0.217<br>*n* =85<br>*P* =0.0461	n.s.	n.s.	n.s.	(213-220)
Uracil	n.s.	*r* =0.237<br>*n* =102<br>*P* =0.0166	n.s.	n.s.	n.s.	n.s.	n.s.	n.s.	n.s.	(121, 221)
Urea	n.s.	n.s.	n.s.	n.s.	*r* =0.293<br>*n* =98<br>*P* =0.0034	*r* =0.285<br>*n* =85<br>*P* =0.0083	n.s.	*r* =0.298<br>*n* =98<br>*P* =0.0029	n.s.	(222, 223)
Xanthosine	n.s.	n.s.	*r* =0.229<br>*n* =102<br>*P* =0.0208	n.s.	n.s.	n.s.	n.s.	n.s.	n.s.	(224-226)

Table S19. Subphenotypes of several typical serum clinical biochemical markers associated with tumor pathophenotypes; n.s., indicates non-significant *P*-values. These data are represented in Figure S7 in Additional file 2.

	Lifespan	Latency	Number of Tumors	Tumor Volume	Tumor Weight	Tumor Growth Rate	Number of Metastasis	Tumor Growth Average Speed
**Final Body Weight**	*r* =0.282 *n* =123 *P* =0.0016	*r* =0.365 n=105 *P* =0.0001	n.s.	n.s.	*r* =-0.242 *n* =97 *P* =0.0169	n.s.	*r* =-0.222 *n* =103 *P* =0.0245	n.s.
**Total Proteins**	*r* =0.195 *n* =146 *P* =0.0181	n.s.	n.s.	n.s.	n.s.	n.s.	n.s.	n.s.
**Glucose**	*r* =0.173 *n* =146 *P* =0.0368	*r* =0.26 *n* =123 *P* =0.0036	n.s.	n.s.	n.s.	n.s.	n.s.	n.s.
**Amylase**	*r* =-0.197 *n* =137 *P* =0.0209	n.s.	*r* =0.199 *n* =137 *P* =0.0199	n.s.	n.s.	n.s.	n.s.	n.s.
**Calcium**	n.s.	n.s.	*r* =0.25 *n* =146 *P* =0.0023	n.s.	n.s.	n.s.	n.s.	n.s.
**Magnesium**	n.s.	n.s.	*r* =-0.162 *n* =145 *P* =0.0509	n.s.	n.s.	n.s.	n.s.	n.s.
**C3-complement**	n.s.	n.s.	*r* =0.253 *n* =140 *P* =0.0026	n.s.	n.s.	*r* =-0.198 *n* =99 *P* =0.0496	n.s.	n.s.
**C4-complement**	n.s.	n.s.	n.s.	*r* =0.216 *n* =119 *P* =0.0183	*r* =0.298 *n* =104 *P* =0.0021	n.s.	n.s.	*r* =0.247 *n* =119 *P* =0.0067

Table S20. Different serum metabolites levels among clusters of prognosis based on tumor pathophenotypes. (a) Serum metabolites that differentiate the cluster of mice without tumors (cluster 5) from the rest of the mice with tumors encompassing a common cluster. (*) t-test. (b) Serum metabolites that distinguish among clusters of mice with tumors at disease-free stage. In both panels (a) and (b), values indicate means with the interquartile range in brackets. The values of section (b) are schematized in **Figure 7C**. Blue: *P*-values with a significant statistical trend ($P < 0.07$). (#) Kruskal-Wallis test; ($) Dunn´s post-test. n.s.: Not statistically significant.

a

**Metabolites**	**Mice Without Tumors (*n* =23)**	**Mice With Tumors (*n* =124)**	***P* -value** (*)
**β-Mannosylglycerate**	23766.00 (26334.50-19757.00)	19788.00 (22972.50-17497.25)	0.018
**2-Hydroxybutanoic acid**	24276.50(28245.50-22199.00)	20511.50 (23988.75-16413.50)	0.003
**Cyclohexylamine**	6454.00 (16410.00-3068.75)	2937.50 (6127.00-2102.25)	0.016

b

**Metabolites**	**Cluster 1 (*n* =25)**	**Cluster 2 (*n* =59)**	**Cluster 3 (*n* =26)**	**Cluster 4 (*n* =14)**	***P* -value** (#)	**Pairwise Comparisons**	
						***P* -value ($)**	**Different Groups**
**Glucuronic acid**	6040 (7123.5-5548.5)	5936 (6713.75-5534)	6687.5 (7512.25-6252.5)	6555 (7833.75-5854.25)	0.029	0.068	**2 < 3**
**Maltose**	1257 (1586.5-922.25)	1141.5 (1330.75-947.75)	1337.5 (1460.5-1111.75)	1554 (1923.75-1187.25)	0.012	0.016	**2 < 4**
**Mannitol**	64696.5 (86881.25-56053.25)	70328.5 (94429.5-53966)	100591.5 (130608-67604)	77246 (111638.75-63554.25)	0.021	0.063	**1 > 3**
						0.033	**2 < 3**
**Sorbitol**	72792 (93457.75-44532.5)	62339 (92528.5-46638.75)	106699 (142164.5-55995)	77319.5 (138577.75-57690.75)	0.043	0.055	**2 < 3**
**N-acetyl-D-mannosamine**	1352.5 (3503-982.25)	1216 (2423.25-886.25)	1360 (3536.5-1123.5)	783 (1200.5-654.75)	0.03	0.027	**3 > 4**
						0.051	**1 > 4**
**Glucose**	924655.5 (963799.25-825673.25)	891082.5 (946184-773137.25)	952293.5 (998030.75-860205.25)	961611.5 (1035505.75-910025.75)	0.021	0.048	**2 < 4**
**Creatinkinase**	306 (687-184.50)	444 (852-189)	678.00 (1332-379.50)	315.00 (594.00-156.00)	0.056	n.s.	
**Lactate dehydrogenase**	999 (1146-651)	867 (1221 -669)	1059.00 (1516.50-817.50)	850.50 (892.50-644.25)	0.051	0.047	**3 > 4**

Table S21. Genomic regions associated with the variable levels of different metabolites simultaneously. These data are schematized in Figure 6. mQTL, metabolic QTL. Pos, position.

Chr.	mQTL	Marker Peak	Pos (Mb)	Metabolite	LOD Score
1	**mQTL-1**	rs6250257	138	**C3-complement**	2.63
		rs13476137	139	**Monopalmitin-1-glyceride**	1.76
	**mQTL-2**	rs13476248	174	**HDL cholesterol**	12.57
				**Total cholesterol**	11.33
	**mQTL-3**	rs3689947	192	**Monopalmitin-1-glyceride**	2.28
2	**mQTL-4**	rs13476540	64	**Iron**	2.70
		rs13476573	73	**Thymidine**	1.65
	**mQTL-5**	rs6193859	158	**Amylase**	2.89
	**mQTL-6**	rs13476913	171	**Sorbitol**	2.15
3	**mQTL-7**	rs13476973	11	**Lactic acid**	1.53
	**mQTL-8**	rs3683507	80	**Cholinesterase (CHE)**	1.97
4	**mQTL-9**	rs6365760	118	**Urea**	2.48
	**mQTL-10**	rs6386918	138	**Alkaline phosphatase**	6.83
5	**mQTL-11**	rs6159963	27	**Oleic acid**	1.71
				**Glycerol**	1.68
		rs6256504	38	**Monopalmitin-1-glyceride**	1.78
	**mQTL-12**	rs3662161	115	**Oleic acid**	1.51
				**4-Hydroxyphenylacetic acid**	2.16
	**mQTL-13**	ut_5_143.236614	142	**Lactic acid**	2.06
6	**mQTL-14**	gnf06.026.418	29	**Cytidine**	2.73
	**mQTL-15**	rs6268125	87	**Ferritin**	4.95
	**mQTL-16**	rs6387265	146	**Octadecanol**	1.57
7	**mQTL-17**	rs3724197	8	**HDL cholesterol**	3.01
				**Total cholesterol**	3.14
	**mQTL-18**	rs3677657	62	**Albumine**	2.76
		rs13479392	76	**Glucose**	2.31
				**Uric Acid**	5.56
				**Calcium**	3.12
				**Phosphorus**	4.25
				**Alanine**	5.28
		rs6279696	81	**Total Proteins**	3.06
				**2-Monopalmitin**	4.32
				**Fumaric acid**	2.98
				**Cystine**	4.71
				**Malic acid**	4.35
				**Alanine**	5.03
				**Tryptophan**	3.37
	**mQTL-19**	rs3708100	99	**Linoleic acid**	2.36
8	**mQTL-20**	cel-8_51076519	51	**Thymidine**	2.69
9	**mQTL-21**	cel-9_29909656	30	**Alanine aminotransferase**	2.27
	**mQTL-22**	rs3693209	72	**Triglycerides**	1.80
				**2-Monopalmitin**	1.78
	**mQTL-23**	rs3694903	114	**Lactate dehydrogenase**	2.45
				**Ferritin**	9.45
10	**mQTL-24**	cel-10_20959105	21	**Succinic acid**	1.62
	**mQTL-25**	rs3688351	104	**Iron**	1.99
11	**mQTL-26**	rs3716790	18	**Sorbose**	1.68
	**mQTL-27**	rs13481032	54	**Fructose**	2.09
				**Linoleic acid**	2.00
				**G-immunogloblulin**	1.57
		rs13481045	58	**Serine**	1.81
				**Sorbitol**	1.69
	**mQTL-28**	rs3698446	105	**Serine**	1.65
12	**mQTL-29**	rs6195664	65	**C4-complement**	1.49
13	**mQTL-30**	rs6215262	4	**Amylase**	3.39
	**mQTL-31**	rs3721858	17	**Iron**	2.94
				**Malic acid**	1.83
	**mQTL-32**	gnf13.057.501	59	**Iron**	1.91
				**Amylase**	1.98
		rs13481839	60	**Amylase**	1.93
				**Sorbose**	1.60
				**Linoleic acid**	1.53
14	**mQTL-33**	rs13482141	35	**Alanine**	1.56
15	**mQTL-34**	rs6326790	91	**Thymidine**	3.30
16	**mQTL-35**	rs4188505	57	**Alkaline phosphatase**	6.32
	**mQTL-36**	rs4207980	78	**Linoleic acid**	1.54
17	**mQTL-37**	rs3657117	67	**Lactate dehydrogenase**	1.86
	**mQTL-38**	rs13459155	86	**Amylase**	1.89
19	**mQTL-39**	rs3686467	16	**Serine**	1.56
				**Uric acid**	1.51
	**mQTL-40**	rs3669236	26	**Glutamine**	1.83
X	**mQTL-41**	rs13483909	92	**Tryptophan**	1.57
	**mQTL-42**	rs13484031	130	**Iron**	1.83

Table S22. Prediction models. The Cox proportional hazards model was used to identify independent prognostic factors that predict (*a*) tumor latency and (*b*) duration of disease (survival with tumor). HR, hazard ratio. For genetic markers (rs6193859, rs4231934, gnf13.057.501, rs3696018 and rs13481230) homozigosity was the reference category. ManNAc, N-acetyl-D-mannosamine. This table is related to Figures 8B and 8C.

a

Latency

Variables in the Equation	*P* -value	HR	95.0% CI for HR	
			Lower	Upper
pERK Liver	0.0052	0.58	0.40	0.85
pAKT(T308) Liver	0.0241	0.66	0.46	0.95
Glucose	0.0092	0.73	0.58	0.93
ManNAc	0.0054	1.37	1.10	1.72
rs6193859	0.0015	0.43	0.25	0.72
rs4231934	0.1088	1.48	0.92	2.39
gnf13.057.501	0.0223	0.56	0.34	0.92

b

Duration of disease

Variables in the Equation	*P* -value	HR	95.0% CI for HR	
			Lower	Upper
Icosenoic acid	0.0002	1.60	1.25	2.06
pAKT (T308) Tumor	<0.0001	0.58	0.45	0.76
Latency	<0.0001	2.49	1.75	3.53
Tumor weight	0.0064	1.52	1.12	2.05
rs3696018	0.0089	0.50	0.30	0.84
rs13481230	0.0836	0.65	0.39	1.06

Table S23. Genetic marker peaks common to some tQTLs, tsQTLs, LsQTLs, and mQTLs. tQTL, tumor QTL. tsQTL, tumor-signaling QTL. LsQTL, liver-signaling QTL. mQTL, metabolic QTL.

Chr.	tQTL	Marker Peak	Location (Mb)	Tumor Pathophenotype	tsQTL	Marker Peak	Location (Mb)	Tumor signaling	LsQTL	Marker Peak	Location (Mb)	Liver Signaling	mQTL	Marker Peak	Location (Mb)	Metabolites
**2**	**tQTL 1**	c2.loc87.5	158.98	Latency									**mQTL5**	rs6193859	158	Amylase
**3**					**tsQTL3**	c3.loc62.5	131.97	pERK1/2	**LsQTL4**	c3.loc57.5	121.97	pAKT2(S474)				
**7**	**tQTL 2**	rs3724197	7.96	Latency<br>Lifespan									**mQTL17**	rs3724197	8.00	HDL cholesterol<br>Total cholesterol
									**LsQTL6**	rs6279696	80.92	mTOR	**mQTL18**	rs3677657<br>rs13479392<br>rs13479392<br>rs13479392<br>rs13479392<br>rs13479392<br>rs6279696<br>rs6279696<br>rs6279696<br>rs6279696<br>rs6279696<br>rs6279696<br>rs6279696<br>rs6279696	61.68<br>75.80<br>75.80<br>75.87<br>75.87<br>76.00<br>80.90<br>81.00<br>81.00<br>81.00<br>81.00<br>81.00<br>81.00<br>81.00	Albumine<br>Glucose<br>Uric Acid<br>Calcium<br>Phosphorus<br>Alanine<br>Total Proteins<br>2-Monopalmitin<br>Fumaric acid<br>Cystine<br>Malic acid<br>Alanine<br>Tryptophan<br>Linoleic acid
**9**					**tsQTL5**	rs6152427	114.11	pAKT1(S473)<br>pAKT(T308)<br>pAKT2(S474)					**mQTL23**	rs3694903	114.00	Lactate dehydrogenase<br>Ferritine
**11**	**tQTL 11**	rs13481230	110.83	Tumor Growth<br>Average Speed					**LsQTL3**	rs3698446	104.91	pAKT(T308)<br>AKT1	**mQTL28**	rs3698446	105.00	Serine
**12**					**tsQTL7**	rs3707048	64.06	pAKT(T308)<br>pAKT1(S473					**mQTL29**	rs6195664	65.50	C4-complement
**13**	**tQTL 3**	gnf13.057.501	58.83	Latency<br>Tumor Incidence<br>Lifespan	**tsQTL11**	rs13481839	59.56	AKT3					**mQTL32**	gnf13.057.501<br>gnf13.057.501<br>rs13481839<br>rs13481839	58.59<br>58.59<br>60.00<br>60.00	Iron<br>Amylase<br>Sorbose<br>Linoleic acid
**15**	**tQTL 7**	rs6326790	90.94	Tumor Incidence					**LsQTL5**	rs6326790	90.94	pAKT2(S474)	**mQTL34**	rs6326790	91.00	Thymidine
**17**	**tQTL 9**	c17.loc25	60.57	Tumor Growth Rate<br>Tumor Weight									**mQTL37**	rs3657117	67.40	Lactate dehydrogenase
**X**	**tQTL 13**	rs13483994	124.29	Number of Metastasis									**mQTL38**	rs13484031	129.85	Iron
