# Supplementary material for: Unraveling heterogeneous susceptibility and the evolution of breast cancer using a systems biology approach
Source: Genome Biol. 2015 Feb 21;16(1):40. doi: 10.1186/s13059-015-0599-z (PMC4389302; doi:10.1186/s13059-015-0599-z)
Supplement: Additional file 2: — Figures S1 to S9. Figure S1. Localization of FVB and F1 mice in the clusters of prognosis. Figure S2. Similarity between human and mouse tumors at the gene expression level. Figure S3. RNA expression analysis in tumors. Figure S4. Distribution of tumors from other clusters along the seven unsupervised clusters formed with the mouse gene expression signature (MCMS) shown in Figure 3A. Figure S5. Evaluation of signaling pathways in tumors and livers from F1BX mice. Figure S6. Analysis of pAKT pathway expression in the tumors. Figure S7. Serum metabolites are associated with cancer progression before disease onset. Figure S8. Signaling levels in tumor, liver, spleen and kidney from FVB and F1 mice. Figure S9. Hypothesis to identify part of the missing heritability. [file 13059_2015_599_MOESM2_ESM.pdf]

Additional File 2

Unraveling heterogeneous susceptibility and the evolution of breast cancer using a systems biology approach

Andres Castellanos-Martin, *et al.*

1) Supplementary figures index…………………………………………….....Page 1

Figure S1. Localization of FVB and F1 mice in the prognosis clusters. *[Related to Figure 1D]*……………………………..Page 2

Figure S2. Similarity between human and backcross tumors at gene expression level. *[Related to Figure 3C]*…………………….............................Page 3

Figure S3. RNA expression analysis in tumors. *[Related to Figure 3]*………………...Page 4

Figure S4. Distribution of tumors from others clusters along the seven unsupervised clusters formed with the mouse gene expression signature (MCMS) shown in Figure 3A. *[Related to Figure 3]*…...................................Page 5

Figure S5. Evaluation of some representative signaling molecules in tumors and livers from F1BX mice. *[Related to Figure 4]*………………………….....Page 6

Figure S6. Analysis of pAKT pathway expression in the tumors. *[Related to Figure 5]* ………………………………....................................Page 7

Figure S7. Serum metabolites are associated with cancer progression before the onset of the disease. *[Related to Figure 7]*………………………………..Page 8

Figure S8. Signaling levels in tumor, liver, spleen and kidney from FVB and F1 mice. ……………………………………………………………………….....Page 9

Figure S9. Hypothesis to identify part of the missing heritability.....................Page 10

2) Supplementary figure legends…………………………………………..........Page 11

Figure S1

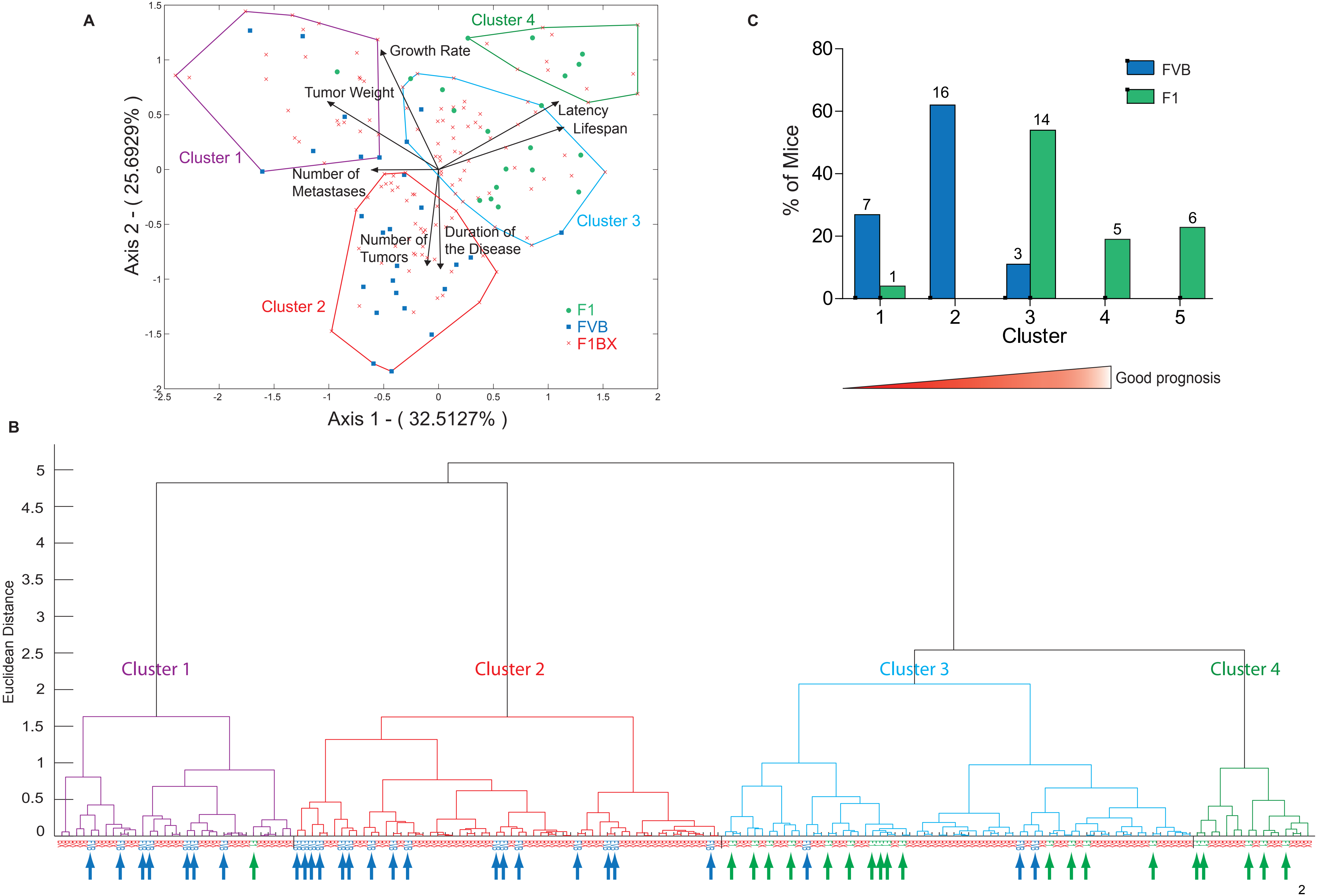
A
Cluster 1
Cluster 2
Cluster 3
Cluster 4
Growth Rate
Tumor Weight
Latency
Lifespan
Number of Metastases
Number of Tumors
Duration of the Disease
F1
FVB
F1BX
Axis 1 - (32.5127%)
Axis 2 - (25.6929%)
B
Euclidean Distance
Cluster 1
Cluster 2
Cluster 3
Cluster 4
C
FVB
F1
% of Mice
Cluster
Good prognosis
7
1
16
3
14
5
6

Figure S2

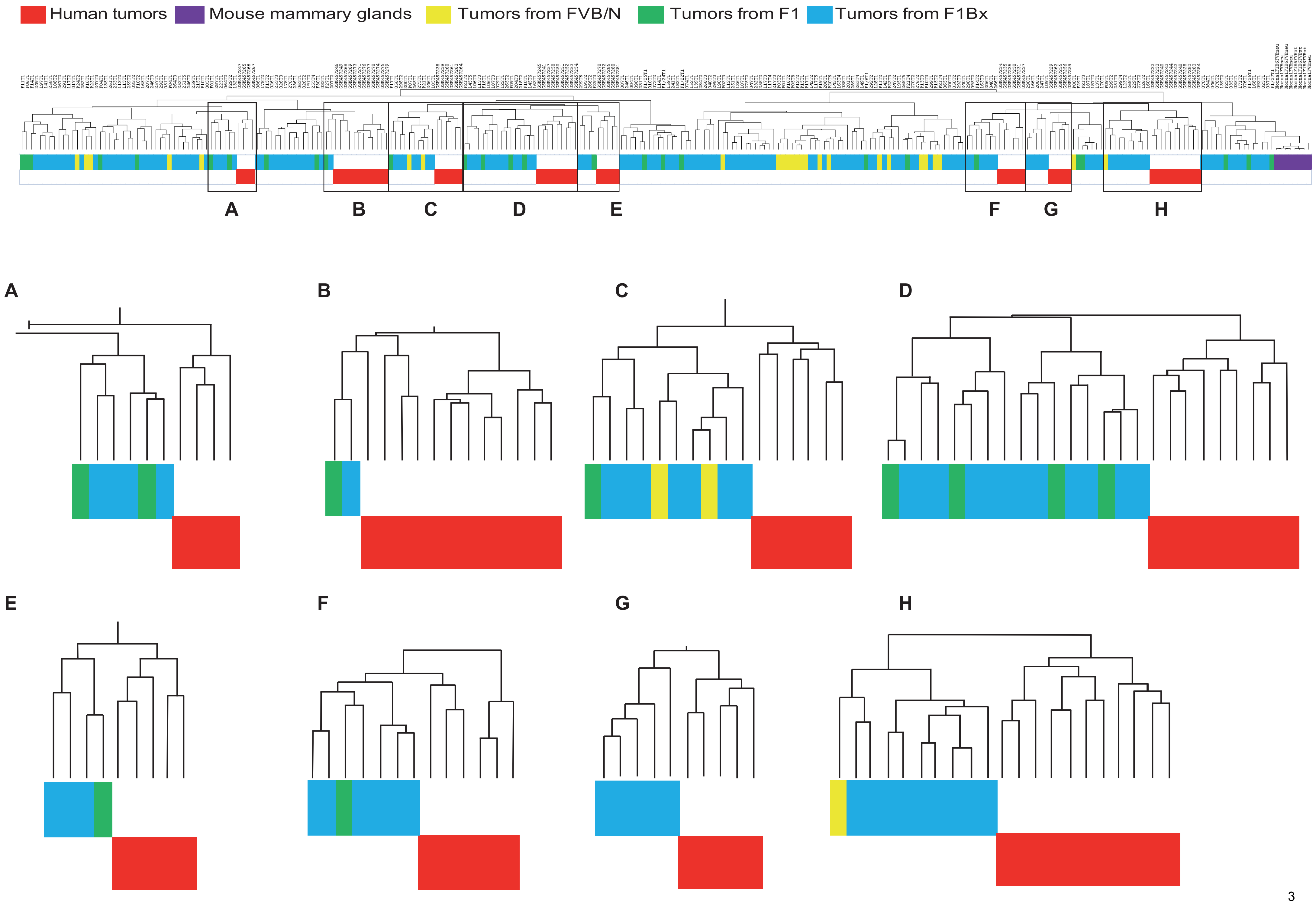
Human tumors
Mouse mammary glands
Tumors from FVB/N
Tumors from F1
Tumors from F1Bx
A
B
C
D
E
F
G
H

Figure S3

A

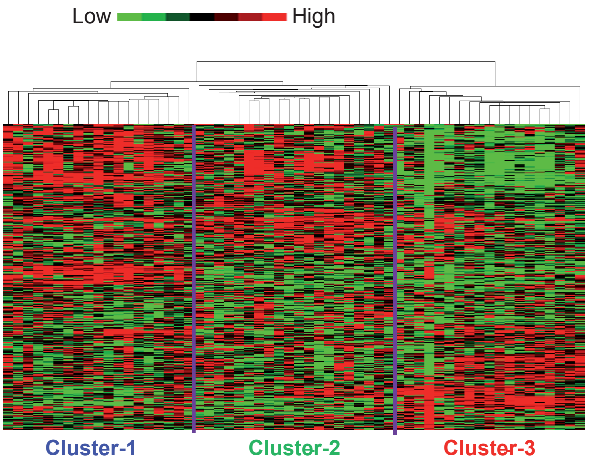

		Clusters (Mouse signature)			Total
		1	2	3	
Borg's Clusters (Human signature)	1	14	5	0	19
	2	2	12	4	18
	3	3	3	15	21
Total		19	20	19	58

B

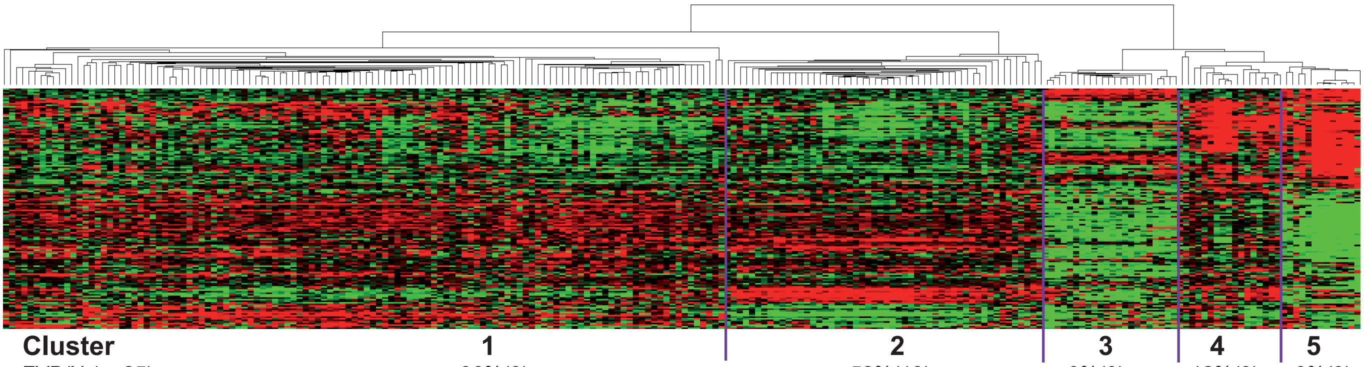

**Cluster**	**1**	**2**	**3**	**4**	**5**
FVB/N (*n*=25)	36%(9)	52%(13)	0%(0)	12%(3)	0%(0)
F1 (*n*=27)	66.7%(18)	3.7%(1)	18.5%(5)	7.4%(2)	3.7%(1)
F1BX (*n*=162)	56.2%(91)	23.4%(38)	10.5%(17)	7.4%(12)	2.5%(4)

C

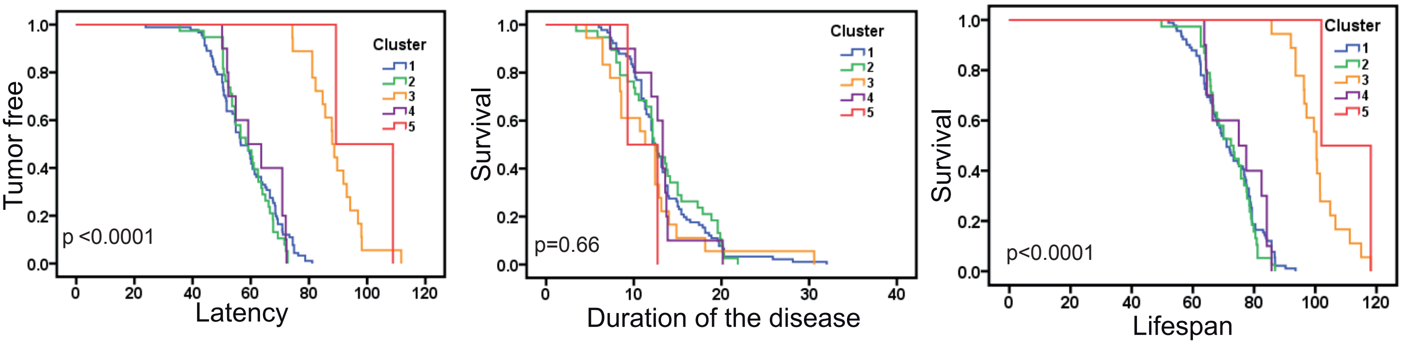

Figure S4

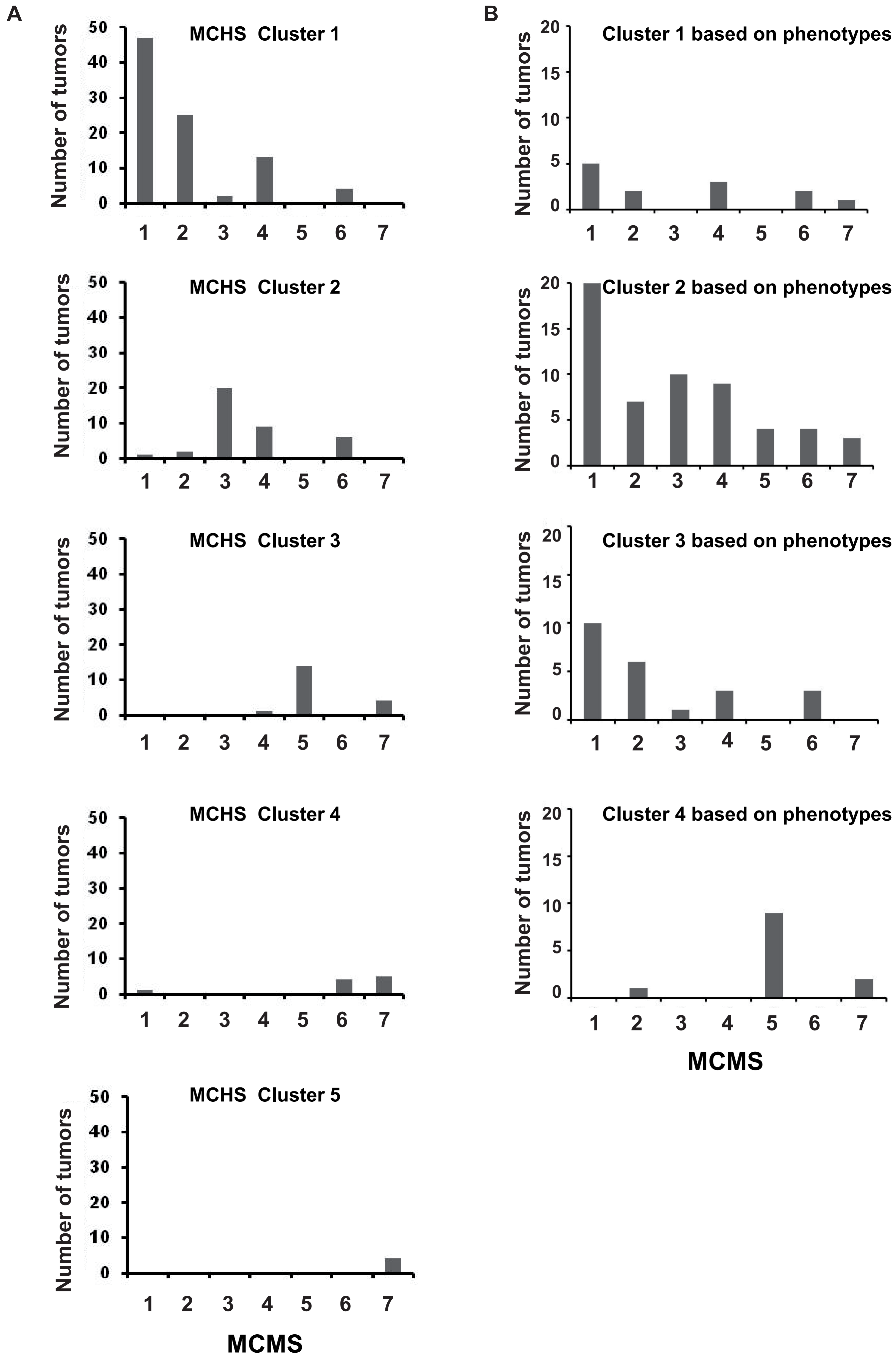
A
MCHS Cluster 1
MCHS Cluster 2
MCHS Cluster 3
MCHS Cluster 4
MCHS Cluster 5
Number of tumors
MCMS
B
Cluster 1 based on phenotypes
Cluster 2 based on phenotypes
Cluster 3 based on phenotypes
Cluster 4 based on phenotypes
Number of tumors
MCMS

Figure S5

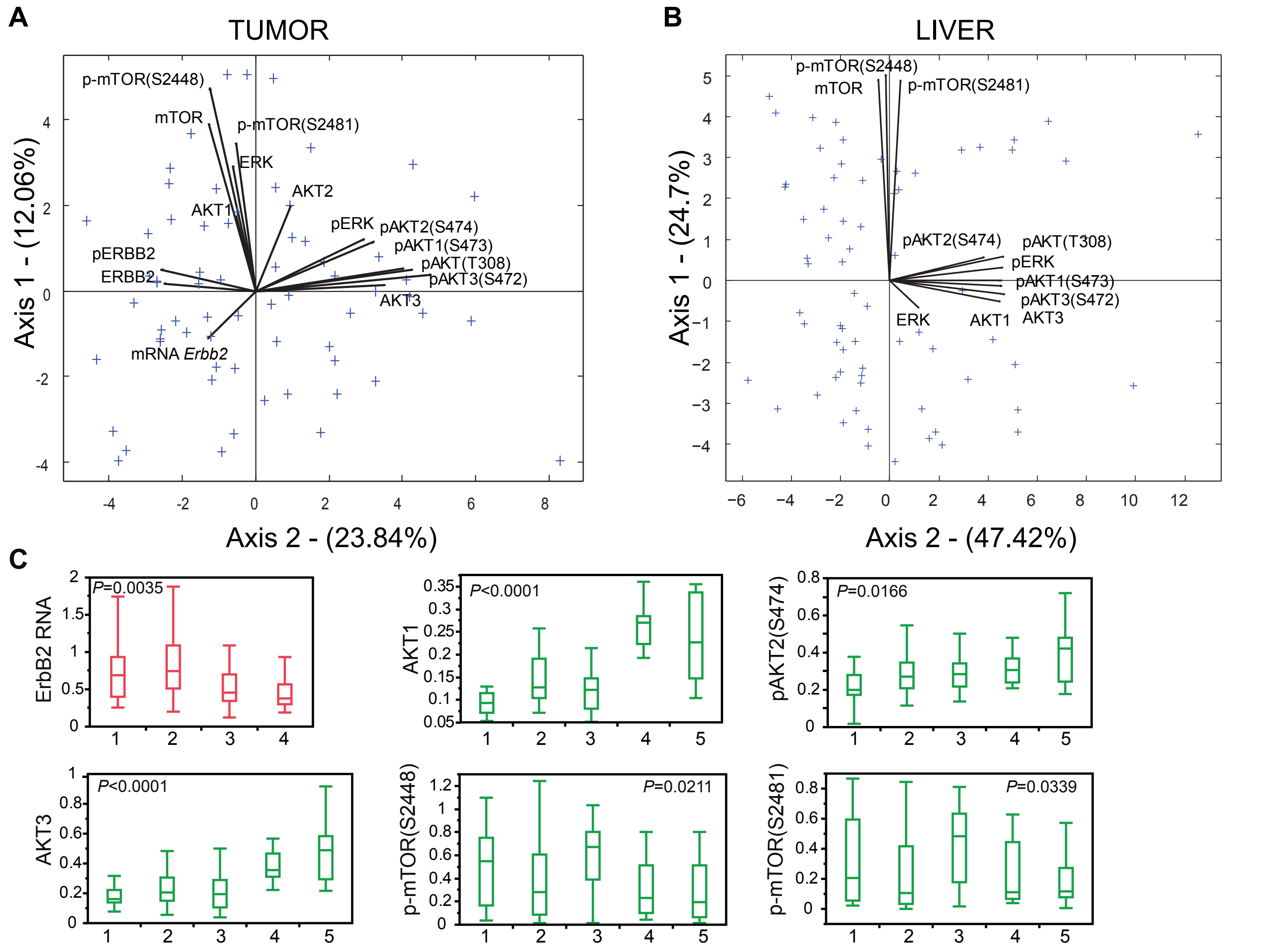

D

	Cluster-2	Cluster-3	Cluster-4
Cluster-1	pAKT(T308)<br>(*P*=0.0499)	pAKT(T308)<br>(*P*=0.0470)	pAKT1(S473) (*P*=0.0003)<br>pAKT3(S472) (*P*=0.0033)<br>pAKT(T308) (*P*<0.0001)<br>pERK1/2 (*P*=0.0277)
Cluster-2		ErbB2 RNA<br>(*P*=0.0198)	ErbB2 RNA (*P*=0.0211)<br>pAKT1(S473) (*P*<0.0001)<br>pAKT(T308) (*P*=0.0096)<br>pAKT3(S472) (*P*=0.0222)

F

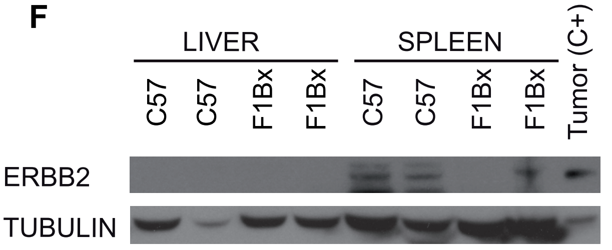

E

	Cluster-2	Cluster-3	Cluster-4	Cluster-5
Cluster-1	pAKT1(S473)<br>(*P*=0.0459)		pERK1/2 (*P*=0.0065)<br>AKT1 (*P*<0.0001)<br>pAKT1(S473) (*P*=0.0010)<br>pAKT(T308) (*P*<0.0001)<br>AKT3 (*P*=0.0001)<br>pAKT3(S472) (*P*=0.0027)	pERK1/2 (*P*=0.0003)<br>AKT1 (*P*<0.0001)<br>pAKT1(S473) (*P*=0.0001)<br>pAKT(T308) (*P*<0.0001)<br>pAKT2(S474) (*P*= 0.0083)<br>AKT3 (*P*<0.0001)<br>pAKT3(S472) (*P*=0.0004)
Cluster-2		p-mTOR(S2481)<br>(*P*=0.0275)	pERK1/2 (*P*=0.0087)<br>AKT1 (*P*<0.0001)<br>pAKT(T308) (*P*= 0.0075)<br>AKT3 (*P*=0.0003)<br>pAKT3(S472) (*P*=0.0327)	pERK1/2 (*P*=0.0002)<br>AKT1 (*P*=0.0016)<br>pAKT1(S473) (*P*=0.0258)<br>pAKT(T308) (*P*= 0.0029)<br>AKT3 (*P*<0.0001)<br>pAKT3(S472) (*P*=0.0047)
Cluster-3			pERK1/2 (*P*=0.0403)<br>AKT1 (*P*<0.0001)<br>pAKT1(S473) (*P*=0.0396)<br>pAKT(T308) (*P*=0.0071)<br>AKT3 (*P*=0.0001)<br>pAKT3(S472) (*P*=0.0072)	pERK1/2 (*P*=0.0021)<br>AKT1 (*P*=0.0001)<br>pAKT1(S473) (*P*=0.0065)<br>pAKT(T308) (*P*=0.0033)<br>AKT3 (*P*<0.0001)<br>pAKT3(S472) (*P*=0.0010)<br>p-mTOR(S2448)(*P*=0.0274)

Figure S6

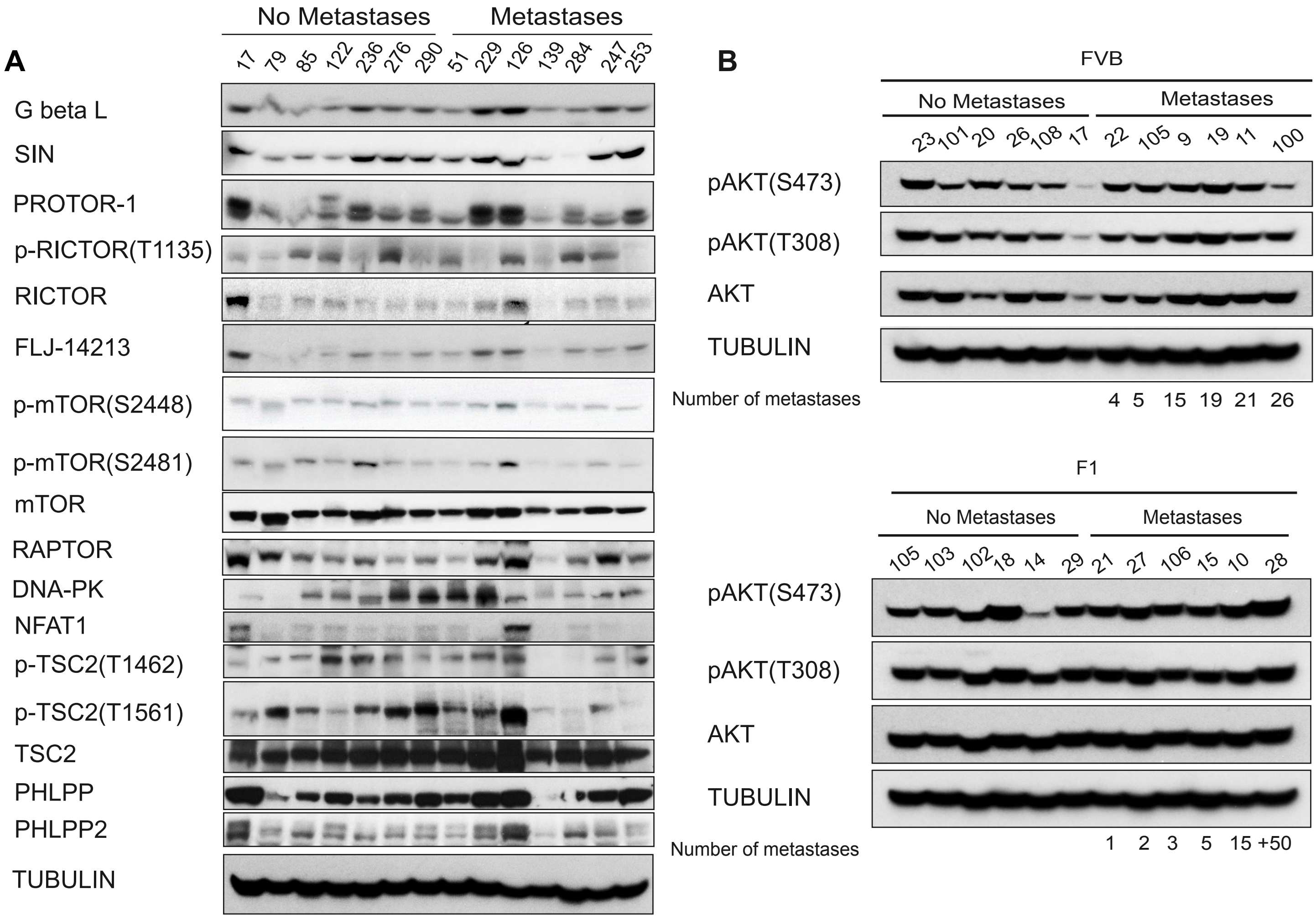
A
No Metastases
Metastases
17 79 85 122 236 276 290 51 229 126 139 284 247 253
G beta L
SIN
PROTOR-1
p-RICTOR(T1135)
RICTOR
FLJ-14213
p-mTOR(S2448)
p-mTOR(S2481)
mTOR
RAPTOR
DNA-PK
NFAT1
p-TSC2(T1462)
p-TSC2(T1561)
TSC2
PHLPP
PHLPP2
TUBULIN
B
FVB
No Metastases
Metastases
23 101 20 26 108 17 22 105 9 19 11 100
pAKT(S473)
pAKT(T308)
AKT
TUBULIN
Number of metastases
4 5 15 19 21 26
F1
No Metastases
Metastases
105 103 102 18 14 29 21 27 106 15 10 28
pAKT(S473)
pAKT(T308)
AKT
TUBULIN
Number of metastases
1 2 3 5 15 +50

Figure S7

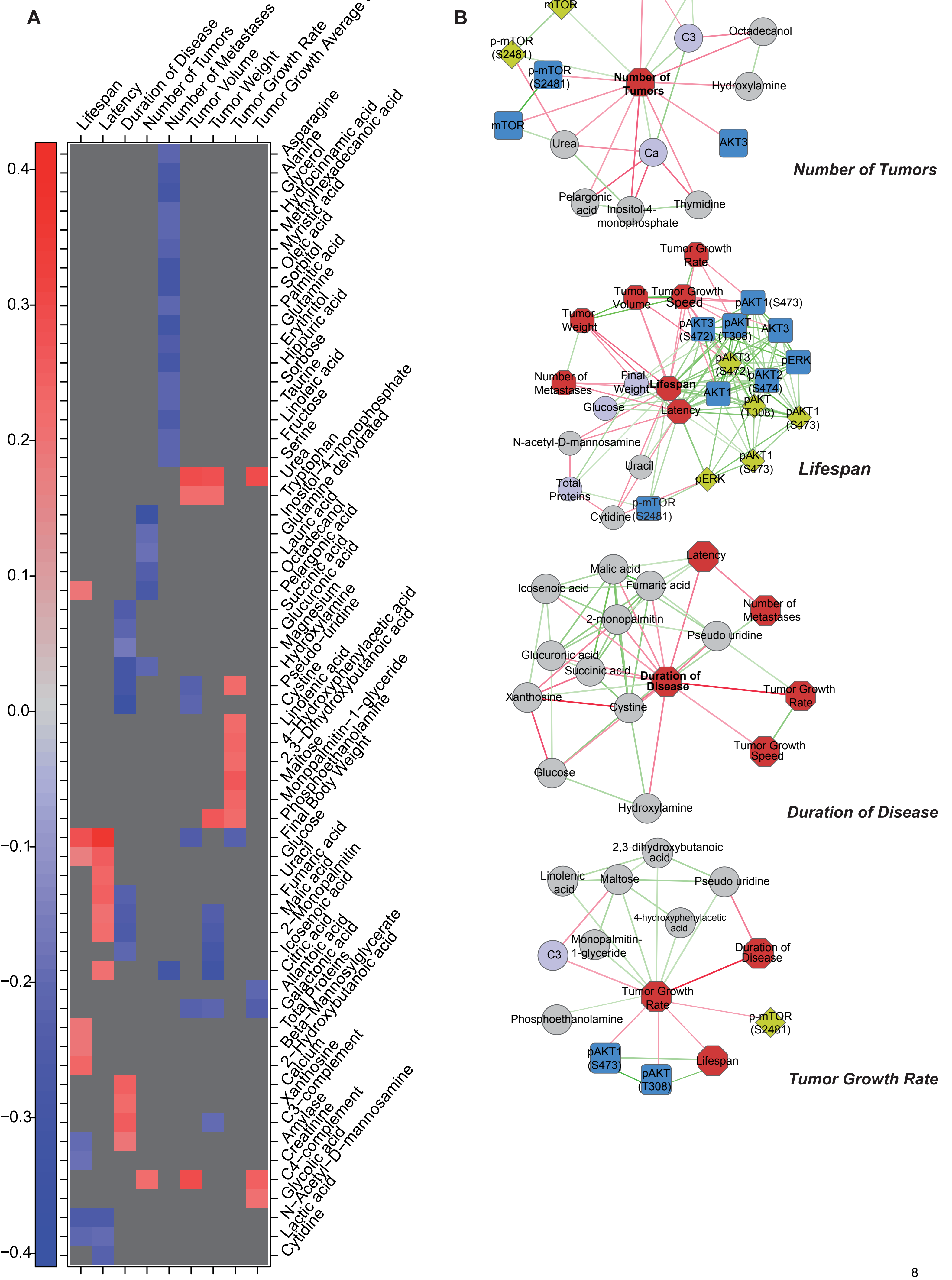
A
Lifespan
Latency
Duration of Disease
Number of Tumors
Number of Metastases
Tumor Volume
Tumor Weight
Tumor Growth Rate
Tumor Growth Average Speed
0.4
0.3
0.2
0.1
0.0
−0.1
−0.2
−0.3
−0.4
Asparagine
Alanine
Glycerol
Hydrocinnamic acid
Methylhexadecanoic acid
Myristic acid
Oleic acid
Sorbitol
Palmitic acid
Glutamine
Erythritol
Hippuric acid
Sorbose
Taurine
Linoleic acid
Fructose
Serine
Urea
Tryptophan
Inositol−4−monophosphate
Glutamine dehydrated
Lauric acid
Octadecanol
Pelargonic acid
Succinic acid
Glucuronic acid
Magnesium
Hydroxylamine
Pseudo−uridine
Cystine
Linolenic acid
4−Hydroxyphenylacetic acid
2,3−Dihydroxybutanoic acid
Maltose
Monopalmitin−1−glyceride
Phosphoethanolamine
Final Body Weight
Glucose
Uracil
Fumaric acid
Malic acid
2−Monopalmitin
Icosenoic acid
Citric acid
Allantoic acid
Galactonic acid
Total Proteins
Beta−Mannosylglycerate
2−Hydroxybutanoic acid
Calcium
Xanthosine
C3−complement
Amylase
Creatinine
C4−complement
Glycolic acid
N−Acetyl−D−mannosamine
Lactic acid
Cytidine
B
ErbB2 RNA
Maltose
mTOR
p-mTOR (S2481)
C3
Octadecanol
Number of Tumors
Hydroxylamine
AKT3
Urea
Ca
Pelargonic acid
Inositol-4-monophosphate
Thymidine
Number of Tumors
Tumor Growth Rate
Tumor Volume
Tumor Growth Speed
pAKT1(S473)
Tumor Weight
pAKT3 (S472)
pAKT (T308)
pERK
Number of Metastases
Final Weight
Lifespan
pAKT2 (S474)
AKT1
Glucose
Latency
N-acetyl-D-mannosamine
Uracil
Total Proteins
Cytidine
Lifespan
Malic acid
Icosenoic acid
Fumaric acid
2-monopalmitin
Pseudo uridine
Glucuronic acid
Succinic acid
Duration of Disease
Xanthosine
Cystine
Glucose
Hydroxylamine
Duration of Disease
2,3-dihydroxybutanoic acid
Linolenic acid
4-hydroxyphenylacetic acid
Monopalmitin-1-glyceride
Phosphoethanolamine
Tumor Growth Rate

Figure S8

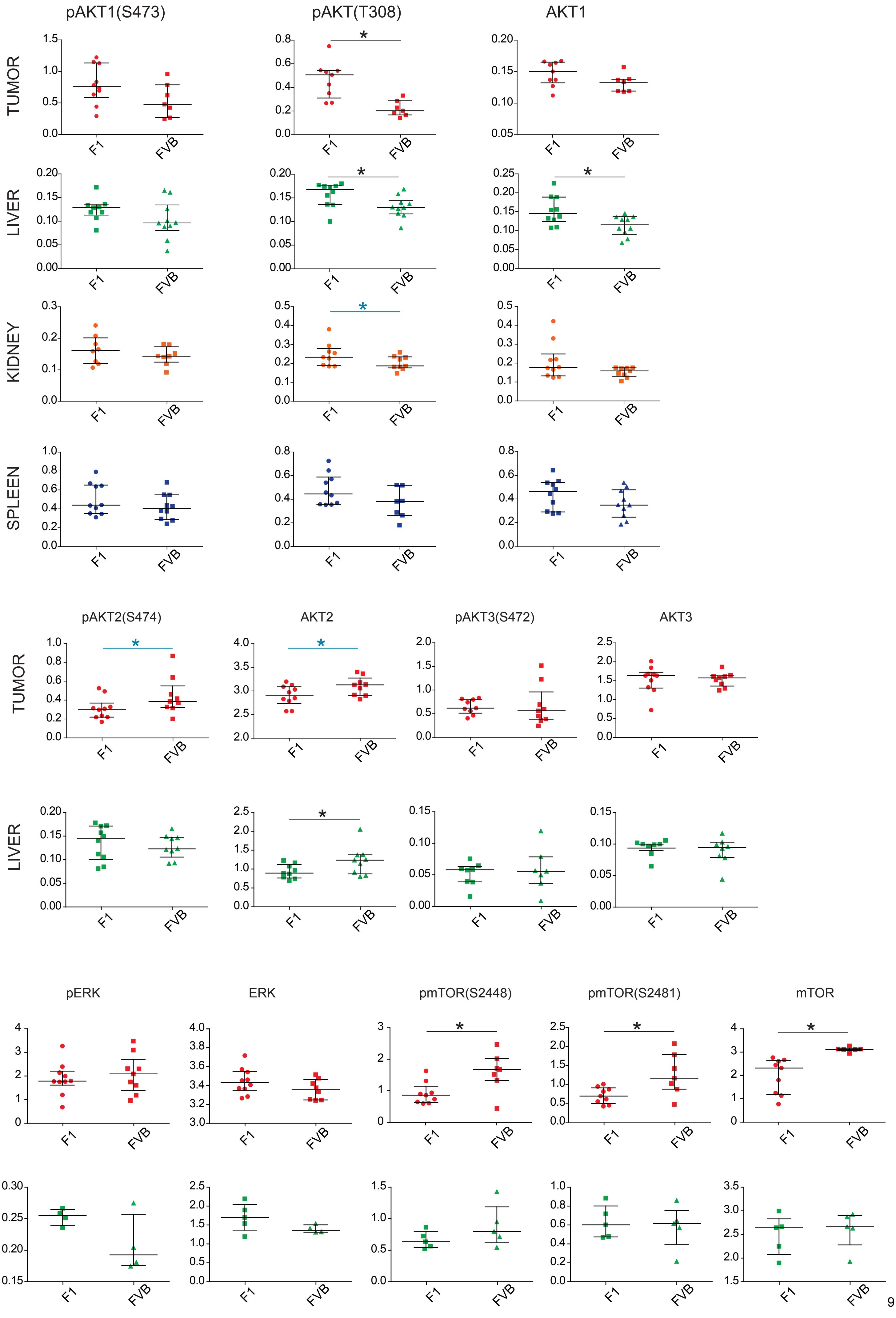

pAKT1(S473)
pAKT(T308)
AKT1
TUMOR
LIVER
KIDNEY
SPLEEN
pAKT2(S474)
AKT2
pAKT3(S472)
AKT3
TUMOR
LIVER
pERK
ERK
pmTOR(S2448)
pmTOR(S2481)
mTOR
TUMOR
LIVER
F1
FVB
*

Figure S9

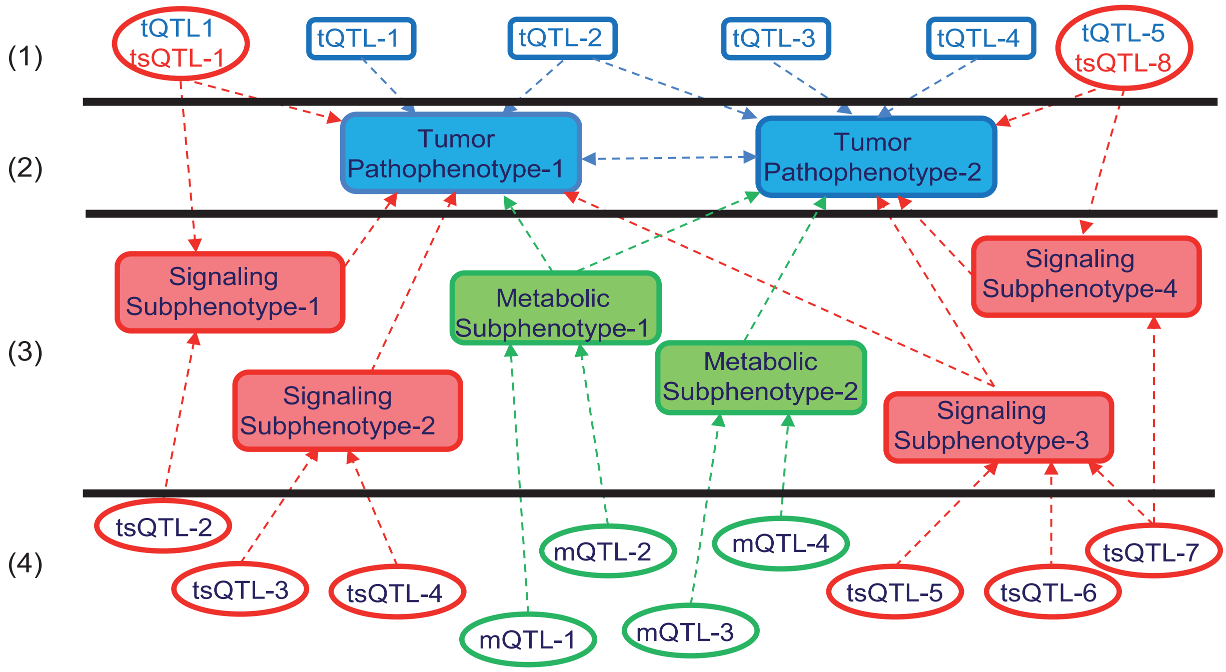

(1)
(2)
(3)
(4)
tQTL1
tsQTL-1
tQTL-1
tQTL-2
tQTL-3
tQTL-4
tQTL-5
tsQTL-8
Tumor Pathophenotype-1
Tumor Pathophenotype-2
Signaling Subphenotype-1
Metabolic Subphenotype-1
Metabolic Subphenotype-2
Signaling Subphenotype-4
Signaling Subphenotype-2
Signaling Subphenotype-3
tsQTL-2
tsQTL-3
tsQTL-4
mQTL-1
mQTL-2
mQTL-3
mQTL-4
tsQTL-5
tsQTL-6
tsQTL-7

Supplementary Figure Legends

Figure S1. Localization of FVB and F1 mice in the prognosis clusters. (Related to Figure 1D). (**A**) The biplot shows that most of the FVB mice are located in the worst prognosis clusters 1 and 2; whereas F1 mice are located in clusters 3 and 4. (**B**) Dendrogram obtained from the biplot to better visualize the location of FVB (blue arrows) and F1 (green arrows) mice. (**C**) Histogram representing the percentage of FVB and F1 mice in each cluster. Here we have included cluster 5, formed by the mice that did not develop tumors. Importantly, this cluster 5 is only formed of F1 and backcross mice (not represented here to better visualize FVB and F1 mice). The absolute number of mice in each cluster is represented in the upper part of each column.

Figure S2. Similarity between human and backcross tumors at gene expression level. (Related to Figure 3C). We calculated the samples closest to the human subgroups. The eight subgroups of human samples (red) are clustered with tumors originated in the backcross population (blue) and sometimes with tumors from the F1 origin (green). The groups of FVB tumors (yellow) and normal mouse mammary glands (purple) are clearly separated from the human tumours. This indicates that tumors originated from mice with a heterogeneous background, mostly backcross and sometimes F1, are more similar to human tumors than those originating in the FVB inbred strain.

Figure S3. RNA expression analysis in tumors. (Related to Figure 3). (**A**) Left: a common list of 354 transcripts between mouse and human tumors allowed a classification of human patients in three unsupervised clusters with prognosis significance as was achieved with the human signature by Staaf *et al.,* 2010. We have included the 354-transcript signature in Table S8 in Additional file 1. Right: the table shows how clusters of

human tumors classified by the human signature in comparison with those classified by the mouse signature tended to be overlapped. Thus, as seen in the table, out of 58 human tumors studied there are 14 common tumors out of 19 (73.7% of overlapping) in cluster 1, 12 out of 18 (66.7%) in cluster 2, and 15 out of 21 (71.4%) in cluster 3. Based on correlation analysis, clusters of human tumors classified by the mouse signature were significantly similar to the clusters identified by Staaf *et al.* (interval by interval; $P = 1.05\ E^{-8}$; $r = 0.667$). (**B**) The signature of 184 transcripts obtained from human ERBB2 tumors by Staaf *et al.* (see text) differentiated five expression clusters in the mouse backcross (Mouse Clusters from Human Signature or MCHS). The percentages and absolute number of tumors from the FVB, F1 and F1BX mice included in each cluster are shown at the bottom. (**C**) The MCHS also had implications in prognosis and the best prognosis clusters in terms of latency and survival in the F1BX mice were MCHS #3 and MCHS #5.

Figure S4. Distribution of tumors from others clusters along the seven unsupervised clusters formed with the mouse gene expression signature (MCMS) shown in Figure 3A. (Supplementary figure related to Figure 3). The Y axis in all graphics indicates the absolute number of tumors; the X axis indicates each of the seven MCMS. (**A**) Distribution of mouse tumors from each of the five clusters originated by the human signature (MCHS) (shown in Figure S3B in Additional file 2) along the seven MCMS; e.g. MCHS #4 contains 17 tumors, of which 9 were included in MCMS #7; 7 tumors in MCMS #6 and 1 tumor in MCMS #1. Notice that the best prognosis MCHS #3 and MCHS #5, as expected, mainly overlapped with the best prognosis MCMS #5 and #7. (**B**) Distribution of the absolute number of tumors from each of the four clusters of prognosis based on the pathophenotypes shown in Figure 1D along the seven MCMS.

Figure S5. Evaluation of some representative signaling molecules in tumors and livers from F1BX mice. (Related to Figure 4). (**A** and **B**) Complex associations identified by principal component analysis among different molecules from signaling pathways in (A) tumors, and (B) livers. (**C**) Distribution of some molecules from signaling pathways along the different prognosis clusters based on tumor pathophenotypes (ANOVA). Tumor (red). Liver (green). (**D** and **E**) Pairwise differentiation between clinical prognosis clusters by some specific signaling molecules from (D) tumors and (E) livers (Tukey´s post-test). Data from panels C-E are shown in Table S10 in Additional file 1. (**F**) We were unable to detect ERBB2 protein in the livers of the F1BX mice (the endogenous protein was detected in spleen). Protein lysates from mouse ERBB2+ tumors were used as positive controls (C+). C57 are protein lysates from livers and spleens of C57BL/6 mice which were used as non-transgenic negative controls.

Figure S6. Analysis of pAKT pathway expression in the tumors. (Related to Figure 5). (**A**) Evaluation of the AKT pathway in F1BX tumors with and without metastasis to the lung. We also analyze these tumors in Figure 5C. (**B**) There were no differences in the expression of total and pAKT levels in tumors from the FVB and F1 mice, regardless of their dissemination capability, indicating that this is a backcross-specific phenotype.

Figure S7. Serum metabolites are associated with cancer progression before the onset of the disease. (Related to Figure 7). (**A**) The figure shows the associations between specific serum metabolites obtained in disease-free mice that later developed tumor traits. All simple correlations with a $P < 0.05$ are included in the figure. The coefficient of correlation (*r*) is represented by a red-blue scale and non-significant correlations are in gray. The values represented are included in Tables S18 and S19 in Additional file 1. (**B**) Networks of subphenotypes associated with different tumor

pathophenotypes. Red edges indicate a negative correlation; green edges a positive correlation. All simple correlations with a $P < 0.05$ and $r > 0.3$ are included (Cytoscape Software).

Figure S8. Signaling levels in tumor, liver, spleen and kidney from FVB and F1 mice. Note the similar behavior of signaling levels in different tissues from the same strains, still present even in non-statistically significant cases. Black and blue asterisks mean $P < 0.05$ and $P < 0.08$ (statistical trend). The values represented in the figure can be found in Tables S11 and S13 in Additional file 1.

Figure S9. Hypothesis to identify part of the "missing heritability". Tumor pathophenotypes (level-2) would be influenced by different subphenotypes such as those related to cell signaling pathways and metabolism (level-3), among others. The variability of each tumor pathophenotype is influenced by the genetic background, which can be demonstrated by the identification of different tQTLs (level-1). tQTLs modify tumor behavior through their influence in known subphenotypes (these are overlapping QTLs such as tQTL1/tsQTL1 and tQTL5/tsQTL8 in the figure) or unknown subphenotypes (the rest of tQTLs at level 1 in the figure). In addition, each different subphenotype from level 3 is also influenced by the genetic background (tsQTLs and mQTLs in the figure) (level 4). Most of the QTLs from level 4 associated with subphenotypes (level 3), in turn related to tumor pathophenotypes at level 2, would not be detected at the tQTL level 1, which would contribute to the "missing heritability" of complex diseases (see Discussion section). The identification of non-overlapping QTLs associated with pathogenic subphenotypes functionally linked to clinical manifestations of the disease would explain and offer a way to identify part of the "missing heritability" characteristic of complex diseases.
