# Supplementary material for: Unraveling heterogeneous susceptibility and the evolution of breast cancer using a systems biology approach
Source: Genome Biol. 2015 Feb 21;16(1):40. doi: 10.1186/s13059-015-0599-z (PMC4389302; doi:10.1186/s13059-015-0599-z)
Supplement: Additional file 3: — Supplementary references for Tables S7 and S18. [file 13059_2015_599_MOESM3_ESM.pdf]

# Additional File 3

## Unraveling heterogeneous susceptibility and the evolution of breast cancer using a systems biology approach

Andres Castellanos-Martin, *et al.*

**Supplementary references index…………………………………………….. Page 1**

Supplementary references for Table S7……………………………………. Page 2

Supplementary references for Table S18…………………………………... Page 11

**Supplementary references for Table S7**


1. Guan XY, Xu J, Anzick SL, Zhang H, Trent JM, Meltzer PS: **Hybrid selection of transcribed sequences from microdissected DNA: isolation of genes within amplified region at 20q11-q13.2 in breast cancer.** *Cancer Res* 1996, **56:**3446-3450.
2. Tanner MM, Grenman S, Koul A, Johannsson O, Meltzer P, Pejovic T, Borg A, Isola JJ: **Frequent amplification of chromosomal region 20q12-q13 in ovarian cancer.** *Clin Cancer Res* 2000, **6:**1833-1839.
3. Rennstam K, Ahlstedt-Soini M, Baldetorp B, Bendahl PO, Borg A, Karhu R, Tanner M, Tirkkonen M, Isola J: **Patterns of chromosomal imbalances defines subgroups of breast cancer with distinct clinical features and prognosis. A study of 305 tumors by comparative genomic hybridization.** *Cancer Res* 2003, **63:**8861-8868.
4. Thomassen M, Tan Q, Kruse TA: **Gene expression meta-analysis identifies chromosomal regions and candidate genes involved in breast cancer metastasis.** *Breast Cancer Res Treat* 2009, **113:**239-249.
5. Gamwell LF, Gambaro K, Merziotis M, Crane C, Arcand SL, Bourada V, Davis C, Squire JA, Huntsman DG, Tonin PN, Vanderhyden BC: **Small cell ovarian carcinoma: genomic stability and responsiveness to therapeutics.** *Orphanet J Rare Dis* 2013, **8:**33.
6. Prescott J, Thompson DJ, Kraft P, Chanock SJ, Audley T, Brown J, Leyland J, Folkerd E, Doody D, Hankinson SE, et al: **Genome-wide association study of circulating estradiol, testosterone, and sex hormone-binding globulin in postmenopausal women.** *PLoS One* 2012, **7:**e37815.
7. Arnutti P, Kotepui M, Asanprakit W, Punyarit P, Chavalitshewinkoon-Petmitr P, Harnroongroj T, Petmitr S: **Determination of whole transcription profiles and specific pathways in invasive ductal breast carcinoma.** *Int J Clin Exp Pathol* 2013, **6:**1112-1120.
8. Shimizu H, Horii A, Sunamura M, Motoi F, Egawa S, Unno M, Fukushige S: **Identification of epigenetically silenced genes in human pancreatic cancer by a novel method "microarray coupled with methyl-CpG targeted transcriptional activation" (MeTA-array).** *Biochem Biophys Res Commun* 2011, **411:**162-167.
9. Agnihotri N, Kumar S, Mehta K: **Tissue transglutaminase as a central mediator in inflammation-induced progression of breast cancer.** *Breast Cancer Res* 2013, **15:**202.
10. Brown KD: **Transglutaminase 2 and NF-kappaB: an odd couple that shapes breast cancer phenotype.** *Breast Cancer Res Treat* 2013, **137:**329-336.
11. Kallioniemi A, Kallioniemi OP, Piper J, Tanner M, Stokke T, Chen L, Smith HS, Pinkel D, Gray JW, Waldman FM: **Detection and mapping of amplified DNA sequences in breast cancer by comparative genomic hybridization.** *Proc Natl Acad Sci U S A* 1994, **91:**2156-2160.
12. Bergamaschi A, Kim YH, Wang P, Sorlie T, Hernandez-Boussard T, Lonning PE, Tibshirani R, Borresen-Dale AL, Pollack JR: **Distinct patterns of DNA copy number alteration are associated with different clinicopathological features and gene-expression subtypes of breast cancer.** *Genes Chromosomes Cancer* 2006, **45:**1033-1040.

13. Richardson AL, Wang ZC, De Nicolo A, Lu X, Brown M, Miron A, Liao X, Iglehart JD, Livingston DM, Ganesan S: **X chromosomal abnormalities in basal-like human breast cancer.** *Cancer cell* 2006, **9:**121-132.
14. Choi C, Kim MH, Juhng SW: **Loss of heterozygosity on chromosome XP22.2-p22.13 and Xq26.1-q27.1 in human breast carcinomas.** *Journal of Korean medical science* 1998, **13:**311-316.
15. Tsou JH, Chang KC, Chang-Liao PY, Yang ST, Lee CT, Chen YP, Lee YC, Lin BW, Lee JC, Shen MR, et al: **Aberrantly expressed AURKC enhances the transformation and tumourigenicity of epithelial cells.** *J Pathol* 2011, **225:**243-254.
16. Duell EJ, Millikan RC, Pittman GS, Winkel S, Lunn RM, Tse CK, Eaton A, Mohrenweiser HW, Newman B, Bell DA: **Polymorphisms in the DNA repair gene XRCC1 and breast cancer.** *Cancer Epidemiol Biomarkers Prev* 2001, **10:**217-222.
17. Saadat M, Ansari-Lari M: **Polymorphism of XRCC1 (at codon 399) and susceptibility to breast cancer, a meta-analysis of the literatures.** *Breast Cancer Res Treat* 2009, **115:**137-144.
18. Buck MB, Knabbe C: **TGF-beta signaling in breast cancer.** *Ann N Y Acad Sci* 2006, **1089:**119-126.
19. Ishiguro T, Avila H, Lin SY, Nakamura T, Yamamoto M, Boyd DD: **Gene trapping identifies chloride channel 4 as a novel inducer of colon cancer cell migration, invasion and metastases.** *British journal of cancer* 2010, **102:**774-782.
20. Loo LW, Grove DI, Williams EM, Neal CL, Cousens LA, Schubert EL, Holcomb IN, Massa HF, Glogovac J, Li CI, et al: **Array comparative genomic hybridization analysis of genomic alterations in breast cancer subtypes.** *Cancer Res* 2004, **64:**8541-8549.
21. Johannsdottir HK, Jonsson G, Johannesdottir G, Agnarsson BA, Eerola H, Arason A, Heikkila P, Egilsson V, Olsson H, Johannsson OT, et al: **Chromosome 5 imbalance mapping in breast tumors from BRCA1 and BRCA2 mutation carriers and sporadic breast tumors.** *Int J Cancer* 2006, **119:**1052-1060.
22. Oldenburg RA, Kroeze-Jansema KH, Houwing-Duistermaat JJ, Bayley JP, Dambrot C, van Asperen CJ, van den Ouweland AM, Bakker B, van Beers EH, Nederlof PM, et al: **Genome-wide linkage scan in Dutch hereditary non-BRCA1/2 breast cancer families identifies 9q21-22 as a putative breast cancer susceptibility locus.** *Genes Chromosomes Cancer* 2008, **47:**947-956.
23. Minobe K, Onda M, Iida A, Kasumi F, Sakamoto G, Nakamura Y, Emi M: **Allelic loss on chromosome 9q is associated with lymph node metastasis of primary breast cancer.** *Jpn J Cancer Res* 1998, **89:**916-922.
24. Schulz WA, Ingenwerth M, Djuidje CE, Hader C, Rahnenfuhrer J, Engers R: **Changes in cortical cytoskeletal and extracellular matrix gene expression in prostate cancer are related to oncogenic ERG deregulation.** *BMC Cancer* 2010, **10:**505.
25. Volinia S, Croce CM: **Prognostic microRNA/mRNA signature from the integrated analysis of patients with invasive breast cancer.** *Proc Natl Acad Sci U S A* 2013, **110:**7413-7417.
26. Rousseau S, Morrice N, Peggie M, Campbell DG, Gaestel M, Cohen P: **Inhibition of SAPK2a/p38 prevents hnRNP A0 phosphorylation by MAPKAP-K2 and its interaction with cytokine mRNAs.** *EMBO J* 2002, **21:**6505-6514.

27. Pihur V, Datta S: **Finding common genes in multiple cancer types through meta-analysis of microarray experiments: a rank aggregation approach.** *Genomics* 2008, **92:**400-403.
28. Wulfkuhle JD, Sgroi DC, Krutzsch H, McLean K, McGarvey K, Knowlton M, Chen S, Shu H, Sahin A, Kurek R, et al: **Proteomics of human breast ductal carcinoma in situ.** *Cancer Res* 2002, **62:**6740-6749.
29. Bonifaci N, Berenguer A, Diez J, Reina O, Medina I, Dopazo J, Moreno V, Pujana MA: **Biological processes, properties and molecular wiring diagrams of candidate low-penetrance breast cancer susceptibility genes.** *BMC Med Genomics* 2008, **1:**62.
30. Broberg K, Huynh E, Schlawicke Engstrom K, Bjork J, Albin M, Ingvar C, Olsson H, Hoglund M: **Association between polymorphisms in RMI1, TOP3A, and BLM and risk of cancer, a case-control study.** *BMC Cancer* 2009, **9:**140.
31. Richard F, Pacyna-Gengelbach M, Schluns K, Fleige B, Winzer KJ, Szymas J, Dietel M, Petersen I, Schwendel A: **Patterns of chromosomal imbalances in invasive breast cancer.** *Int J Cancer* 2000, **89:**305-310.
32. Dellas A, Torhorst J, Schultheiss E, Mihatsch MJ, Moch H: **DNA sequence losses on chromosomes 11p and 18q are associated with clinical outcome in lymph node-negative ductal breast cancer.** *Clin Cancer Res* 2002, **8:**1210-1216.
33. Yokota T, Matsumoto S, Yoshimoto M, Kasumi F, Akiyama F, Sakamoto G, Nakamura Y, Emi M: **Mapping of a breast cancer tumor suppressor gene locus to a 4-cM interval on chromosome 18q21.** *Jpn J Cancer Res* 1997, **88:**959-964.
34. Jakob J, Nagase S, Gazdar A, Chien M, Morozova I, Russo JJ, Nandula SV, Murty VV, Li CM, Tycko B, Parsons R: **Two somatic biallelic lesions within and near SMAD4 in a human breast cancer cell line.** *Genes Chromosomes Cancer* 2005, **42:**372-383.
35. Kim DH, Yoon DS, Dooley WC, Nam ES, Ryu JW, Jung KC, Park HR, Sohn JH, Shin HS, Park YE: **Association of maspin expression with the high histological grade and lymphocyte-rich stroma in early-stage breast cancer.** *Histopathology* 2003, **42:**37-42.
36. Maass N, Hojo T, Rosel F, Ikeda T, Jonat W, Nagasaki K: **Down regulation of the tumor suppressor gene maspin in breast carcinoma is associated with a higher risk of distant metastasis.** *Clin Biochem* 2001, **34:**303-307.
37. Petry IB, Fieber E, Schmidt M, Gehrmann M, Gebhard S, Hermes M, Schormann W, Selinski S, Freis E, Schwender H, et al: **ERBB2 induces an antiapoptotic expression pattern of Bcl-2 family members in node-negative breast cancer.** *Clin Cancer Res* 2010, **16:**451-460.
38. Whyte J, Bergin O, Bianchi A, McNally S, Martin F: **Key signalling nodes in mammary gland development and cancer. Mitogen-activated protein kinase signalling in experimental models of breast cancer progression and in mammary gland development.** *Breast Cancer Res* 2009, **11:**209.
39. Kytola S, Rummukainen J, Nordgren A, Karhu R, Farnebo F, Isola J, Larsson C: **Chromosomal alterations in 15 breast cancer cell lines by comparative genomic hybridization and spectral karyotyping.** *Genes, chromosomes & cancer* 2000, **28:**308-317.

40. Draheim KM, Chen HB, Tao Q, Moore N, Roche M, Lyle S: **ARRDC3 suppresses breast cancer progression by negatively regulating integrin beta4.** *Oncogene* 2010, **29:**5032-5047.
41. Olson JE, Wang X, Pankratz VS, Fredericksen ZS, Vachon CM, Vierkant RA, Cerhan JR, Couch FJ: **Centrosome-related genes, genetic variation, and risk of breast cancer.** *Breast cancer research and treatment* 2011, **125:**221-228.
42. Le Dily F, Metivier R, Gueguen MM, Le Peron C, Flouriot G, Tas P, Pakdel F: **COUP-TFI modulates estrogen signaling and influences proliferation, survival and migration of breast cancer cells.** *Breast cancer research and treatment* 2008, **110:**69-83.
43. Nygaard S, Jacobsen A, Lindow M, Eriksen J, Balslev E, Flyger H, Tolstrup N, Moller S, Krogh A, Litman T: **Identification and analysis of miRNAs in human breast cancer and teratoma samples using deep sequencing.** *BMC medical genomics* 2009, **2:**35.
44. Fu YP, Yu JC, Cheng TC, Lou MA, Hsu GC, Wu CY, Chen ST, Wu HS, Wu PE, Shen CY: **Breast cancer risk associated with genotypic polymorphism of the nonhomologous end-joining genes: a multigenic study on cancer susceptibility.** *Cancer research* 2003, **63:**2440-2446.
45. Orphanos V, McGown G, Hey Y, Boyle JM, Santibanez-Koref M: **Proximal 6q, a region showing allele loss in primary breast cancer.** *British journal of cancer* 1995, **71:**290-293.
46. Weber-Mangal S, Sinn HP, Popp S, Klaes R, Emig R, Bentz M, Mansmann U, Bastert G, Bartram CR, Jauch A: **Breast cancer in young women (< or = 35 years): Genomic aberrations detected by comparative genomic hybridization.** *International journal of cancer Journal international du cancer* 2003, **107:**583-592.
47. Sheng ZM, Marchetti A, Buttitta F, Champeme MH, Campani D, Bistocchi M, Lidereau R, Callahan R: **Multiple regions of chromosome 6q affected by loss of heterozygosity in primary human breast carcinomas.** *British journal of cancer* 1996, **73:**144-147.
48. Fox BP, Kandpal RP: **Invasiveness of breast carcinoma cells and transcript profile: Eph receptors and ephrin ligands as molecular markers of potential diagnostic and prognostic application.** *Biochemical and biophysical research communications* 2004, **318:**882-892.
49. Neil JR, Schiemann WP: **Altered TAB1:I kappaB kinase interaction promotes transforming growth factor beta-mediated nuclear factor-kappaB activation during breast cancer progression.** *Cancer research* 2008, **68:**1462-1470.
50. Li H, Lahti JM, Valentine M, Saito M, Reed SI, Look AT, Kidd VJ: **Molecular cloning and chromosomal localization of the human cyclin C (CCNC) and cyclin E (CCNE) genes: deletion of the CCNC gene in human tumors.** *Genomics* 1996, **32:**253-259.
51. Sun M, Srikantan V, Ma L, Li J, Zhang W, Petrovics G, Makarem M, Strovel JW, Horrigan SG, Augustus M, et al: **Characterization of frequently deleted 6q locus in prostate cancer.** *DNA and cell biology* 2006, **25:**597-607.
52. Jiao Y, Cui L, Gao C, Li W, Zhao X, Liu S, Wu M, Deng G, Li Z: **CASP8AP2 is a promising prognostic indicator in pediatric acute lymphoblastic leukemia.** *Leukemia research* 2012, **36:**67-71.

53. Antoniou AC, Kuchenbaecker KB, Soucy P, Beesley J, Chen X, McGuffog L, Lee A, Barrowdale D, Healey S, Sinilnikova OM, et al: **Common variants at 12p11, 12q24, 9p21, 9q31.2 and in ZNF365 are associated with breast cancer risk for BRCA1 and/or BRCA2 mutation carriers.** *Breast cancer research : BCR* 2012, **14:**R33.
54. Ghoussaini M, Fletcher O, Michailidou K, Turnbull C, Schmidt MK, Dicks E, Dennis J, Wang Q, Humphreys MK, Luccarini C, et al: **Genome-wide association analysis identifies three new breast cancer susceptibility loci.** *Nature genetics* 2012, **44:**312-318.
55. Bourdon V, Naef F, Rao PH, Reuter V, Mok SC, Bosl GJ, Koul S, Murty VV, Kucherlapati RS, Chaganti RS: **Genomic and expression analysis of the 12p11-p12 amplicon using EST arrays identifies two novel amplified and overexpressed genes.** *Cancer research* 2002, **62:**6218-6223.
56. Castells A, Gusella JF, Ramesh V, Rustgi AK: **A region of deletion on chromosome 22q13 is common to human breast and colorectal cancers.** *Cancer research* 2000, **60:**2836-2839.
57. Deneen B, Welford SM, Ho T, Hernandez F, Kurland I, Denny CT: **PIM3 proto-oncogene kinase is a common transcriptional target of divergent EWS/ETS oncoproteins.** *Molecular and cellular biology* 2003, **23:**3897-3908.
58. Hirano A, Emi M, Tsuneizumi M, Utada Y, Yoshimoto M, Kasumi F, Akiyama F, Sakamoto G, Haga S, Kajiwara T, Nakamura Y: **Allelic losses of loci at 3p25.1, 8p22, 13q12, 17p13.3, and 22q13 correlate with postoperative recurrence in breast cancer.** *Clinical cancer research : an official journal of the American Association for Cancer Research* 2001, **7:**876-882.
59. Dong-Dong L: **Up-regulation expression of MLC1 in human liver cancer tissue and enhanced SMMC7721 cell tumorigenesis in vivo and vitro.** *Hepato-gastroenterology* 2005, **52:**1186-1190.
60. Popivanova BK, Li YY, Zheng H, Omura K, Fujii C, Tsuneyama K, Mukaida N: **Proto-oncogene, Pim-3 with serine/threonine kinase activity, is aberrantly expressed in human colon cancer cells and can prevent Bad-mediated apoptosis.** *Cancer science* 2007, **98:**321-328.
61. Zheng HC, Tsuneyama K, Takahashi H, Miwa S, Sugiyama T, Popivanova BK, Fujii C, Nomoto K, Mukaida N, Takano Y: **Aberrant Pim-3 expression is involved in gastric adenoma-adenocarcinoma sequence and cancer progression.** *Journal of cancer research and clinical oncology* 2008, **134:**481-488.
62. Osada H, Tatematsu Y, Saito H, Yatabe Y, Mitsudomi T, Takahashi T: **Reduced expression of class II histone deacetylase genes is associated with poor prognosis in lung cancer patients.** *International journal of cancer Journal international du cancer* 2004, **112:**26-32.
63. Schwertfeger KL: **Fibroblast growth factors in development and cancer: insights from the mammary and prostate glands.** *Current drug targets* 2009, **10:**632-644.
64. Kymionis GD, Dimitrakakis CE, Konstadoulakis MM, Arzimanoglou I, Leandros E, Chalkiadakis G, Keramopoulos A, Michalas S: **Can expression of apoptosis genes, bcl-2 and bax, predict survival and responsiveness to chemotherapy in node-negative breast cancer patients?** *The Journal of surgical research* 2001, **99:**161-168.

65. Gibson LF, Fortney J, Magro G, Ericson SG, Lynch JP, Landreth KS: **Regulation of BAX and BCL-2 expression in breast cancer cells by chemotherapy.** *Breast cancer research and treatment* 1999, **55:**107-117.
66. Shi SQ, Xu L, Zhao G, Yang Y, Peng JP: **Apoptosis and tumor inhibition induced by human chorionic gonadotropin beta in mouse breast carcinoma.** *Journal of molecular medicine* 2006, **84:**933-941.
67. Bieche I, Tozlu S, Girault I, Onody P, Driouch K, Vidaud M, Lidereau R: **Expression of PEA3/E1AF/ETV4, an Ets-related transcription factor, in breast tumors: positive links to MMP2, NRG1 and CGB expression.** *Carcinogenesis* 2004, **25:**405-411.
68. Yu Y, Feig LA: **Involvement of R-Ras and Ral GTPases in estrogen-independent proliferation of breast cancer cells.** *Oncogene* 2002, **21:**7557-7568.
69. Li G, Li W, Angelastro JM, Greene LA, Liu DX: **Identification of a novel DNA binding site and a transcriptional target for activating transcription factor 5 in c6 glioma and mcf-7 breast cancer cells.** *Molecular cancer research : MCR* 2009, **7:**933-943.
70. Ansems M, Hontelez S, Looman MW, Karthaus N, Bult P, Bonenkamp JJ, Jansen JH, Sweep FC, Span PN, Adema GJ: **DC-SCRIPT: nuclear receptor modulation and prognostic significance in primary breast cancer.** *Journal of the National Cancer Institute* 2010, **102:**54-68.
71. Csiszar K, Fong SF, Ujfalusi A, Krawetz SA, Salvati EP, Mackenzie JW, Boyd CD: **Somatic mutations of the lysyl oxidase gene on chromosome 5q23.1 in colorectal tumors.** *International journal of cancer Journal international du cancer* 2002, **97:**636-642.
72. Loo LW, Ton C, Wang YW, Grove DI, Bouzek H, Vartanian N, Lin MG, Yuan X, Lawton TL, Daling JR, et al: **Differential patterns of allelic loss in estrogen receptor-positive infiltrating lobular and ductal breast cancer.** *Genes, chromosomes & cancer* 2008, **47:**1049-1066.
73. Allan GJ, Cottrell S, Trowsdale J, Foulkes WD: **Loss of heterozygosity on chromosome 5 in sporadic ovarian carcinoma is a late event and is not associated with mutations in APC at 5q21-22.** *Human mutation* 1994, **3:**283-291.
74. Schoch C, Kohlmann A, Dugas M, Kern W, Hiddemann W, Schnittger S, Haferlach T: **Genomic gains and losses influence expression levels of genes located within the affected regions: a study on acute myeloid leukemias with trisomy 8, 11, or 13, monosomy 7, or deletion 5q.** *Leukemia* 2005, **19:**1224-1228.
75. Xiao GG, Zhou BS, Somlo G, Portnow J, Juhasz A, Un F, Chew H, Gandara D, Yen Y: **Identification of F-box/LLR-repeated protein 17 as potential useful biomarker for breast cancer therapy.** *Cancer genomics & proteomics* 2008, **5:**151-160.
76. Pasder O, Shpungin S, Salem Y, Makovsky A, Vilchick S, Michaeli S, Malovani H, Nir U: **Downregulation of Fer induces PP1 activation and cell-cycle arrest in malignant cells.** *Oncogene* 2006, **25:**4194-4206.
77. Przybytkowski E, Girouard S, Allard B, Lamarre L, Basik M: **Widespread bimodal intrachromosomal genomic instability in sporadic breast cancers associated with 13q allelic imbalance.** *Cancer research* 2003, **63:**4588-4593.
78. Guan XY, Meltzer PS, Dalton WS, Trent JM: **Identification of cryptic sites of DNA sequence amplification in human breast cancer by chromosome microdissection.** *Nature genetics* 1994, **8:**155-161.

79. Lowery AJ, Miller N, McNeill RE, Kerin MJ: **MicroRNAs as prognostic indicators and therapeutic targets: potential effect on breast cancer management.** *Clinical cancer research : an official journal of the American Association for Cancer Research* 2008, **14:**360-365.
80. Hossain A, Kuo MT, Saunders GF: **Mir-17-5p regulates breast cancer cell proliferation by inhibiting translation of AIB1 mRNA.** *Molecular and cellular biology* 2006, **26:**8191-8201.
81. Leivonen SK, Makela R, Ostling P, Kohonen P, Haapa-Paananen S, Kleivi K, Enerly E, Aakula A, Hellstrom K, Sahlberg N, et al: **Protein lysate microarray analysis to identify microRNAs regulating estrogen receptor signaling in breast cancer cell lines.** *Oncogene* 2009, **28:**3926-3936.
82. Zhang X, Yu H, Lou JR, Zheng J, Zhu H, Popescu NI, Lupu F, Lind SE, Ding WQ: **MicroRNA-19 (miR-19) regulates tissue factor expression in breast cancer cells.** *The Journal of biological chemistry* 2011, **286:**1429-1435.
83. Li JY, Zhang Y, Zhang WH, Jia S, Kang Y, Zhu XY: **Differential distribution of miR-20a and miR-20b may underly metastatic heterogeneity of breast cancers.** *Asian Pacific journal of cancer prevention : APJCP* 2012, **13:**1901-1906.
84. Nilsson S, Moller C, Jirstrom K, Lee A, Busch S, Lamb R, Landberg G: **Downregulation of miR-92a is associated with aggressive breast cancer features and increased tumour macrophage infiltration.** *PLoS One* 2012, **7:**e36051.
85. Plummer SJ, Adams L, Simmons JA, Casey G: **Localization of a growth suppressor activity in MCF7 breast cancer cells to chromosome 17q24-q25.** *Oncogene* 1997, **14:**2339-2345.
86. Knuutila S, Bjorkqvist AM, Autio K, Tarkkanen M, Wolf M, Monni O, Szymanska J, Larramendy ML, Tapper J, Pere H, et al: **DNA copy number amplifications in human neoplasms: review of comparative genomic hybridization studies.** *The American journal of pathology* 1998, **152:**1107-1123.
87. Forozan F, Mahlamaki EH, Monni O, Chen Y, Veldman R, Jiang Y, Gooden GC, Ethier SP, Kallioniemi A, Kallioniemi OP: **Comparative genomic hybridization analysis of 38 breast cancer cell lines: a basis for interpreting complementary DNA microarray data.** *Cancer research* 2000, **60:**4519-4525.
88. Jonsson G, Staaf J, Olsson E, Heidenblad M, Vallon-Christersson J, Osoegawa K, de Jong P, Oredsson S, Ringner M, Hoglund M, Borg A: **High-resolution genomic profiles of breast cancer cell lines assessed by tiling BAC array comparative genomic hybridization.** *Genes, chromosomes & cancer* 2007, **46:**543-558.
89. Yook JI, Li XY, Ota I, Hu C, Kim HS, Kim NH, Cha SY, Ryu JK, Choi YJ, Kim J, et al: **A Wnt-Axin2-GSK3beta cascade regulates Snail1 activity in breast cancer cells.** *Nature cell biology* 2006, **8:**1398-1406.
90. Proikas-Cezanne T, Waddell S, Gaugel A, Frickey T, Lupas A, Nordheim A: **WIPI-1alpha (WIPI49), a member of the novel 7-bladed WIPI protein family, is aberrantly expressed in human cancer and is linked to starvation-induced autophagy.** *Oncogene* 2004, **23:**9314-9325.

91. Pramanik R, Qi X, Borowicz S, Choubey D, Schultz RM, Han J, Chen G: **p38 isoforms have opposite effects on AP-1-dependent transcription through regulation of c-Jun. The determinant roles of the isoforms in the p38 MAPK signal specificity.** *The Journal of biological chemistry* 2003, **278:**4831-4839.
92. Arribas R, Ribas M, Risques RA, Masramon L, Tortola S, Marcuello E, Aiza G, Miro R, Capella G, Peinado MA: **Prospective assessment of allelic losses at 4p14-16 in colorectal cancer: two mutational patterns and a locus associated with poorer survival.** *Clinical cancer research : an official journal of the American Association for Cancer Research* 1999, **5:**3454-3459.
93. Sun J, Wiklund F, Zheng SL, Chang B, Balter K, Li L, Johansson JE, Li G, Adami HO, Liu W, et al: **Sequence variants in Toll-like receptor gene cluster (TLR6-TLR1-TLR10) and prostate cancer risk.** *Journal of the National Cancer Institute* 2005, **97:**525-532.
94. Poh W, Wong W, Ong H, Aung MO, Lim SG, Chua BT, Ho HK: **Klotho-beta overexpression as a novel target for suppressing proliferation and fibroblast growth factor receptor-4 signaling in hepatocellular carcinoma.** *Molecular cancer* 2012, **11:**14.
95. Ulrich CM, Curtin K, Potter JD, Bigler J, Caan B, Slattery ML: **Polymorphisms in the reduced folate carrier, thymidylate synthase, or methionine synthase and risk of colon cancer.** *Cancer epidemiology, biomarkers & prevention : a publication of the American Association for Cancer Research, cosponsored by the American Society of Preventive Oncology* 2005, **14:**2509-2516.
96. Eklof V, Van Guelpen B, Hultdin J, Johansson I, Hallmans G, Palmqvist R: **The reduced folate carrier (RFC1) 80G > A and folate hydrolase 1 (FOLH1) 1561C > T polymorphisms and the risk of colorectal cancer: a nested case-referent study.** *Scandinavian journal of clinical and laboratory investigation* 2008, **68:**393-401.
97. Shivapurkar N, Sood S, Wistuba, II, Virmani AK, Maitra A, Milchgrub S, Minna JD, Gazdar AF: **Multiple regions of chromosome 4 demonstrating allelic losses in breast carcinomas.** *Cancer research* 1999, **59:**3576-3580.
98. Naylor TL, Greshock J, Wang Y, Colligon T, Yu QC, Clemmer V, Zaks TZ, Weber BL: **High resolution genomic analysis of sporadic breast cancer using array-based comparative genomic hybridization.** *Breast cancer research : BCR* 2005, **7:**R1186-1198.
99. Kiemeney LA, Sulem P, Besenbacher S, Vermeulen SH, Sigurdsson A, Thorleifsson G, Gudbjartsson DF, Stacey SN, Gudmundsson J, Zanon C, et al: **A sequence variant at 4p16.3 confers susceptibility to urinary bladder cancer.** *Nature genetics* 2010, **42:**415-419.
100. Bassett T, Harpur B, Poon HY, Kuo KH, Lee CH: **Effective stimulation of growth in MCF-7 human breast cancer cells by inhibition of syntaxin18 by external guide sequence and ribonuclease P.** *Cancer letters* 2008, **272:**167-175.
101. Sliwinski T, Synowiec E, Czarny P, Gomulak P, Forma E, Morawiec Z, Morawiec J, Dziki L, Wasylecka M, Blasiak J: **The c.469+46_56del mutation in the homeobox MSX1 gene--a novel risk factor in breast cancer?** *Cancer epidemiology* 2010, **34:**652-655.

102. Shih JY, Yang SC, Hong TM, Yuan A, Chen JJ, Yu CJ, Chang YL, Lee YC, Peck K, Wu CW, Yang PC: **Collapsin response mediator protein-1 and the invasion and metastasis of cancer cells.** *Journal of the National Cancer Institute* 2001, **93:**1392-1400.
103. Okai I, Wang L, Gong L, Arko-Boham B, Hao L, Zhou X, Qi X, Hu J, Shao S: **Overexpression of JAKMIP1 associates with Wnt/beta-catenin pathway activation and promotes cancer cell proliferation in vitro.** *Biomedicine & pharmacotherapy = Biomedecine & pharmacotherapie* 2013, **67:**228-234.
104. Saito S, Morita K, Hirano T: **High frequency of common DNA copy number abnormalities detected by bacterial artificial chromosome array comparative genomic hybridization in 24 breast cancer cell lines.** *Human cell* 2009, **22:**1-10.
105. Mitra AB, Murty VV, Li RG, Pratap M, Luthra UK, Chaganti RS: **Allelotype analysis of cervical carcinoma.** *Cancer research* 1994, **54:**4481-4487.
106. Edelmann J, Richter K, Hanel C, Hering S, Horn LC: **X chromosomal and autosomal loss of heterozygosity and microsatellite instability in human cervical carcinoma.** *International journal of gynecological cancer : official journal of the International Gynecological Cancer Society* 2006, **16:**1248-1253.
107. Guey LT, Garcia-Closas M, Murta-Nascimento C, Lloreta J, Palencia L, Kogevinas M, Rothman N, Vellalta G, Calle ML, Marenne G, et al: **Genetic susceptibility to distinct bladder cancer subphenotypes.** *European urology* 2010, **57:**283-292.
108. Kawamata H, Furihata T, Omotehara F, Sakai T, Horiuchi H, Shinagawa Y, Imura J, Ohkura Y, Tachibana M, Kubota K, et al: **Identification of genes differentially expressed in a newly isolated human metastasizing esophageal cancer cell line, T.Tn-AT1, by cDNA microarray.** *Cancer science* 2003, **94:**699-706.
109. Laes JF, Quan X, Ravoet M, Stieber D, Van Vooren P, Van Reeth T, Szpirer J, Szpirer C: **Analysis of candidate genes included in the mammary cancer susceptibility 1 (Mcs1) region.** *Mammalian genome : official journal of the International Mammalian Genome Society* 2001, **12:**199-206.
110. Karlsson E, Danielsson A, Delle U, Olsson B, Karlsson P, Helou K: **Chromosomal changes associated with clinical outcome in lymph node-negative breast cancer.** *Cancer genetics and cytogenetics* 2007, **172:**139-146.
111. Zhu Y, Stevens RG, Leaderer D, Hoffman A, Holford T, Zhang Y, Brown HN, Zheng T: **Non-synonymous polymorphisms in the circadian gene NPAS2 and breast cancer risk.** *Breast cancer research and treatment* 2008, **107:**421-425.
112. Collins CS, Hong J, Sapinoso L, Zhou Y, Liu Z, Micklash K, Schultz PG, Hampton GM: **A small interfering RNA screen for modulators of tumor cell motility identifies MAP4K4 as a promigratory kinase.** *Proceedings of the National Academy of Sciences of the United States of America* 2006, **103:**3775-3780.
113. Carolan BJ, Heguy A, Harvey BG, Leopold PL, Ferris B, Crystal RG: **Up-regulation of expression of the ubiquitin carboxyl-terminal hydrolase L1 gene in human airway epithelium of cigarette smokers.** *Cancer research* 2006, **66:**10729-10740.
114. Rahmatpanah FB, Carstens S, Guo J, Sjahputera O, Taylor KH, Duff D, Shi H, Davis JW, Hooshmand SI, Chitma-Matsiga R, Caldwell CW: **Differential DNA methylation patterns of small B-cell lymphoma subclasses with different clinical behavior.** *Leukemia* 2006, **20:**1855-1862.

**Supplementary references for Table S18**


115. Denkert C, Bucher E, Hilvo M, Salek R, Oresic M, Griffin J, Brockmoller S, Klauschen F, Loibl S, Barupal DK, et al: **Metabolomics of human breast cancer: new approaches for tumor typing and biomarker discovery.** *Genome medicine* 2012, **4:**37.
116. Qiu Y, Cai G, Su M, Chen T, Zheng X, Xu Y, Ni Y, Zhao A, Xu LX, Cai S, Jia W: **Serum metabolite profiling of human colorectal cancer using GC-TOFMS and UPLC-QTOFMS.** *Journal of proteome research* 2009, **8:**4844-4850.
117. Yoshida M, Hatano N, Nishiumi S, Irino Y, Izumi Y, Takenawa T, Azuma T: **Diagnosis of gastroenterological diseases by metabolome analysis using gas chromatography-mass spectrometry.** *Journal of gastroenterology* 2012, **47:**9-20.
118. Yabushita S, Fukamachi K, Tanaka H, Fukuda T, Sumida K, Deguchi Y, Mikata K, Nishioka K, Kawamura S, Uwagawa S, et al: **Metabolomic and transcriptomic profiling of human K-ras oncogene transgenic rats with pancreatic ductal adenocarcinomas.** *Carcinogenesis* 2013, **34:**1251-1259.
119. Kobayashi T, Nishiumi S, Ikeda A, Yoshie T, Sakai A, Matsubara A, Izumi Y, Tsumura H, Tsuda M, Nishisaki H, et al: **A novel serum metabolomics-based diagnostic approach to pancreatic cancer.** *Cancer epidemiology, biomarkers & prevention : a publication of the American Association for Cancer Research, cosponsored by the American Society of Preventive Oncology* 2013, **22:**571-579.
120. Han J, Talorete TP, Yamada P, Isoda H: **Anti-proliferative and apoptotic effects of oleuropein and hydroxytyrosol on human breast cancer MCF-7 cells.** *Cytotechnology* 2009, **59:**45-53.
121. Hori S, Nishiumi S, Kobayashi K, Shinohara M, Hatakeyama Y, Kotani Y, Hatano N, Maniwa Y, Nishio W, Bamba T, et al: **A metabolomic approach to lung cancer.** *Lung cancer* 2011, **74:**284-292.
122. Albers MJ, Bok R, Chen AP, Cunningham CH, Zierhut ML, Zhang VY, Kohler SJ, Tropp J, Hurd RE, Yen YF, et al: **Hyperpolarized 13C lactate, pyruvate, and alanine: noninvasive biomarkers for prostate cancer detection and grading.** *Cancer research* 2008, **68:**8607-8615.
123. Tessem MB, Swanson MG, Keshari KR, Albers MJ, Joun D, Tabatabai ZL, Simko JP, Shinohara K, Nelson SJ, Vigneron DB, et al: **Evaluation of lactate and alanine as metabolic biomarkers of prostate cancer using 1H HR-MAS spectroscopy of biopsy tissues.** *Magnetic resonance in medicine : official journal of the Society of Magnetic Resonance in Medicine / Society of Magnetic Resonance in Medicine* 2008, **60:**510-516.
124. Wetzler M, Sanford BL, Kurtzberg J, DeOliveira D, Frankel SR, Powell BL, Kolitz JE, Bloomfield CD, Larson RA: **Effective asparagine depletion with pegylated asparaginase results in improved outcomes in adult acute lymphoblastic leukemia: Cancer and Leukemia Group B Study 9511.** *Blood* 2007, **109:**4164-4167.
125. Tiziani S, Lopes V, Gunther UL: **Early stage diagnosis of oral cancer using 1H NMR-based metabolomics.** *Neoplasia* 2009, **11:**269-276, 264p following 269.
126. Morvan D, Demidem A: **Metabolomics by proton nuclear magnetic resonance spectroscopy of the response to chloroethylnitrosourea reveals drug efficacy and tumor adaptive metabolic pathways.** *Cancer research* 2007, **67:**2150-2159.

127. Miyagi Y, Higashiyama M, Gochi A, Akaike M, Ishikawa T, Miura T, Saruki N, Bando E, Kimura H, Imamura F, et al: **Plasma free amino acid profiling of five types of cancer patients and its application for early detection.** *PLoS One* 2011, **6:**e24143.
128. Potter VR, Busch H: **Citric acid content of normal and tumor tissues in vivo following injection of fluoroacetate.** *Cancer research* 1950, **10:**353-356.
129. Costello LC, Franklin RB, Narayan P: **Citrate in the diagnosis of prostate cancer.** *The Prostate* 1999, **38:**237-245.
130. Zhang W, Trachootham D, Liu J, Chen G, Pelicano H, Garcia-Prieto C, Lu W, Burger JA, Croce CM, Plunkett W, et al: **Stromal control of cystine metabolism promotes cancer cell survival in chronic lymphocytic leukaemia.** *Nature cell biology* 2012, **14:**276-286.
131. Doxsee DW, Gout PW, Kurita T, Lo M, Buckley AR, Wang Y, Xue H, Karp CM, Cutz JC, Cunha GR, Wang YZ: **Sulfasalazine-induced cystine starvation: potential use for prostate cancer therapy.** *The Prostate* 2007, **67:**162-171.
132. Eck HP, Mertens T, Rosokat H, Fatkenheuer G, Pohl C, Schrappe M, Daniel V, Naher H, Petzoldt D, Drings P, et al.: **T4+ cell numbers are correlated with plasma glutamate and cystine levels: association of hyperglutamataemia with immunodeficiency in diseases with different aetiologies.** *International immunology* 1992, **4:**7-13.
133. Ma Y, Liu G, Du M, Stayton I: **Recent developments in the determination of urinary cancer biomarkers by capillary electrophoresis.** *Electrophoresis* 2004, **25:**1473-1484.
134. Jeng LB, Lo WY, Hsu WY, Lin WD, Lin CT, Lai CC, Tsai FJ: **Analysis of urinary nucleosides as helper tumor markers in hepatocellular carcinoma diagnosis.** *Rapid communications in mass spectrometry : RCM* 2009, **23:**1543-1549.
135. Bullinger D, Frohlich H, Klaus F, Neubauer H, Frickenschmidt A, Henneges C, Zell A, Laufer S, Gleiter CH, Liebich H, Kammerer B: **Bioinformatical evaluation of modified nucleosides as biomedical markers in diagnosis of breast cancer.** *Analytica chimica acta* 2008, **618:**29-34.
136. Catchpole G, Platzer A, Weikert C, Kempkensteffen C, Johannsen M, Krause H, Jung K, Miller K, Willmitzer L, Selbig J, Weikert S: **Metabolic profiling reveals key metabolic features of renal cell carcinoma.** *Journal of cellular and molecular medicine* 2011, **15:**109-118.
137. Urayama S, Zou W, Brooks K, Tolstikov V: **Comprehensive mass spectrometry based metabolic profiling of blood plasma reveals potent discriminatory classifiers of pancreatic cancer.** *Rapid communications in mass spectrometry : RCM* 2010, **24:**613-620.
138. Stamp D, Zhang XM, Medline A, Bruce WR, Archer MC: **Sucrose enhancement of the early steps of colon carcinogenesis in mice.** *Carcinogenesis* 1993, **14:**777-779.
139. Giovannucci E, Rimm EB, Wolk A, Ascherio A, Stampfer MJ, Colditz GA, Willett WC: **Calcium and fructose intake in relation to risk of prostate cancer.** *Cancer research* 1998, **58:**442-447.
140. Liu H, Huang D, McArthur DL, Boros LG, Nissen N, Heaney AP: **Fructose induces transketolase flux to promote pancreatic cancer growth.** *Cancer research* 2010, **70:**6368-6376.
141. Boone CW, Steele VE, Kelloff GJ: **Screening for chemopreventive (anticarcinogenic) compounds in rodents.** *Mutation research* 1992, **267:**251-255.

142. Kelloff GJ, Boone CW, Crowell JA, Steele VE, Lubet R, Sigman CC: **Chemopreventive drug development: perspectives and progress.** *Cancer epidemiology, biomarkers & prevention : a publication of the American Association for Cancer Research, cosponsored by the American Society of Preventive Oncology* 1994, **3:**85-98.
143. Kuroda K, Terao K, Akao M: **Inhibitory effect of fumaric acid on hepatocarcinogenesis by thioacetamide in rats.** *Journal of the National Cancer Institute* 1987, **79:**1047-1051.
144. Akao M, Kuroda K: **Inhibitory effect of fumaric acid on hepatocarcinogenesis by thioacetamide in mice.** *Chemical & pharmaceutical bulletin* 1990, **38:**2012-2014.
145. Bezabeh T, Ijare OB, Albiin N, Arnelo U, Lindberg B, Smith IC: **Detection and quantification of D-glucuronic acid in human bile using 1H NMR spectroscopy: relevance to the diagnosis of pancreatic cancer.** *Magma* 2009, **22:**267-275.
146. El-Mezayen HA, Toson el SA, Darwish H, Metwally FM: **Development of a novel metastatic breast cancer score based on hyaluronic acid metabolism.** *Medical oncology* 2013, **30:**404.
147. Dickinson LE, Gerecht S: **Micropatterned surfaces to study hyaluronic acid interactions with cancer cells.** *Journal of visualized experiments : JoVE* 2010.
148. Wang TP, Pan YR, Fu CY, Chang HY: **Down-regulation of UDP-glucose dehydrogenase affects glycosaminoglycans synthesis and motility in HCT-8 colorectal carcinoma cells.** *Experimental cell research* 2010, **316:**2893-2902.
149. Stern R: **Hyaluronan catabolism: a new metabolic pathway.** *European journal of cell biology* 2004, **83:**317-325.
150. Katt WP, Cerione RA: **Glutaminase regulation in cancer cells: a druggable chain of events.** *Drug discovery today* 2013.
151. Hensley CT, Wasti AT, DeBerardinis RJ: **Glutamine and cancer: cell biology, physiology, and clinical opportunities.** *The Journal of clinical investigation* 2013, **123:**3678-3684.
152. Daye D, Wellen KE: **Metabolic reprogramming in cancer: unraveling the role of glutamine in tumorigenesis.** *Seminars in cell & developmental biology* 2012, **23:**362-369.
153. DeBerardinis RJ, Cheng T: **Q's next: the diverse functions of glutamine in metabolism, cell biology and cancer.** *Oncogene* 2010, **29:**313-324.
154. Shanware NP, Mullen AR, DeBerardinis RJ, Abraham RT: **Glutamine: pleiotropic roles in tumor growth and stress resistance.** *Journal of molecular medicine* 2011, **89:**229-236.
155. Jobard E, Pontoizeau C, Blaise BJ, Bachelot T, Elena-Herrmann B, Tredan O: **A serum nuclear magnetic resonance-based metabolomic signature of advanced metastatic human breast cancer.** *Cancer letters* 2013.
156. Cao B, Li M, Zha W, Zhao Q, Gu R, Liu L, Shi J, Zhou J, Zhou F, Wu X, et al: **Metabolomic approach to evaluating adriamycin pharmacodynamics and resistance in breast cancer cells.** *Metabolomics : Official journal of the Metabolomic Society* 2013, **9:**960-973.
157. Cao XC, Zhang WR, Cao WF, Liu BW, Zhang F, Zhao HM, Meng R, Zhang L, Niu RF, Hao XS, Zhang B: **Aquaporin3 is required for FGF-2-induced migration of human breast cancers.** *PLoS One* 2013, **8:**e56735.

158. Verkman AS: **Aquaporins in clinical medicine.** *Annual review of medicine* 2012, **63:**303-316.
159. Hong JT, Kim EJ, Ahn KS, Jung KM, Yun YP, Park YK, Lee SH: **Inhibitory effect of glycolic acid on ultraviolet-induced skin tumorigenesis in SKH-1 hairless mice and its mechanism of action.** *Molecular carcinogenesis* 2001, **31:**152-160.
160. Ahn KS, Park KS, Jung KM, Jung HK, Lee SH, Chung SY, Yang KH, Yun YP, Pyo HB, Park YK, et al: **Inhibitory effect of glycolic acid on ultraviolet B-induced c-fos expression, AP-1 activation and p53-p21 response in a human keratinocyte cell line.** *Cancer letters* 2002, **186:**125-135.
161. Henning SM, Wang P, Abgaryan N, Vicinanza R, de Oliveira DM, Zhang Y, Lee RP, Carpenter CL, Aronson WJ, Heber D: **Phenolic acid concentrations in plasma and urine from men consuming green or black tea and potential chemopreventive properties for colon cancer.** *Molecular nutrition & food research* 2013, **57:**483-493.
162. Mutsaers HA, van den Heuvel LP, Ringens LH, Dankers AC, Russel FG, Wetzels JF, Hoenderop JG, Masereeuw R: **Uremic toxins inhibit transport by breast cancer resistance protein and multidrug resistance protein 4 at clinically relevant concentrations.** *PLoS One* 2011, **6:**e18438.
163. Kim KB, Yang JY, Kwack SJ, Park KL, Kim HS, Ryu do H, Kim YJ, Hwang GS, Lee BM: **Toxicometabolomics of urinary biomarkers for human gastric cancer in a mouse model.** *Journal of toxicology and environmental health Part A* 2010, **73:**1420-1430.
164. Zhen Y, Krausz KW, Chen C, Idle JR, Gonzalez FJ: **Metabolomic and genetic analysis of biomarkers for peroxisome proliferator-activated receptor alpha expression and activation.** *Molecular endocrinology* 2007, **21:**2136-2151.
165. Oda M, Ueno T, Kasai N, Takahashi H, Yoshida H, Sugawara F, Sakaguchi K, Hayashi H, Mizushina Y: **Inhibition of telomerase by linear-chain fatty acids: a structural analysis.** *The Biochemical journal* 2002, **367:**329-334.
166. Alonso T, Morgan RO, Marvizon JC, Zarbl H, Santos E: **Malignant transformation by ras and other oncogenes produces common alterations in inositol phospholipid signaling pathways.** *Proceedings of the National Academy of Sciences of the United States of America* 1988, **85:**4271-4275.
167. Todo T, Fahlbusch R: **Accumulation of inositol phosphates in low-passage human meningioma cells following treatment with epidermal growth factor.** *Journal of neurosurgery* 1994, **80:**890-896.
168. Choi SY, Collins CC, Gout PW, Wang Y: **Cancer-generated lactic acid: a regulatory, immunosuppressive metabolite?** *The Journal of pathology* 2013, **230:**350-355.
169. Feron O: **Pyruvate into lactate and back: from the Warburg effect to symbiotic energy fuel exchange in cancer cells.** *Radiotherapy and oncology : journal of the European Society for Therapeutic Radiology and Oncology* 2009, **92:**329-333.
170. Zhou W, Liotta LA, Petricoin EF: **Cancer metabolism: what we can learn from proteomic analysis by mass spectrometry.** *Cancer genomics & proteomics* 2012, **9:**373-381.
171. Icard P, Lincet H: **A global view of the biochemical pathways involved in the regulation of the metabolism of cancer cells.** *Biochimica et biophysica acta* 2012, **1826:**423-433.

172. Giskeodegard GF, Lundgren S, Sitter B, Fjosne HE, Postma G, Buydens LM, Gribbestad IS, Bathen TF: **Lactate and glycine-potential MR biomarkers of prognosis in estrogen receptor-positive breast cancers.** *NMR in biomedicine* 2012, **25:**1271-1279.
173. Kuwata S, Ohkubo K, Kumamoto S, Yamaguchi N, Izuka N, Murota K, Tsujiuchi T, Iwamori M, Fukushima N: **Extracellular lipid metabolism influences the survival of ovarian cancer cells.** *Biochemical and biophysical research communications* 2013, **439:**280-284.
174. Zollner A, Dragan CA, Pistorius D, Muller R, Bode HB, Peters FT, Maurer HH, Bureik M: **Human CYP4Z1 catalyzes the in-chain hydroxylation of lauric acid and myristic acid.** *Biological chemistry* 2009, **390:**313-317.
175. Yu W, Chai H, Li Y, Zhao H, Xie X, Zheng H, Wang C, Wang X, Yang G, Cai X, et al: **Increased expression of CYP4Z1 promotes tumor angiogenesis and growth in human breast cancer.** *Toxicology and applied pharmacology* 2012, **264:**73-83.
176. Bartsch H, Nair J, Owen RW: **Dietary polyunsaturated fatty acids and cancers of the breast and colorectum: emerging evidence for their role as risk modifiers.** *Carcinogenesis* 1999, **20:**2209-2218.
177. Aro A, Mannisto S, Salminen I, Ovaskainen ML, Kataja V, Uusitupa M: **Inverse association between dietary and serum conjugated linoleic acid and risk of breast cancer in postmenopausal women.** *Nutrition and cancer* 2000, **38:**151-157.
178. Heinze VM, Actis AB: **Dietary conjugated linoleic acid and long-chain n-3 fatty acids in mammary and prostate cancer protection: a review.** *International journal of food sciences and nutrition* 2012, **63:**66-78.
179. Ip C: **Review of the effects of trans fatty acids, oleic acid, n-3 polyunsaturated fatty acids, and conjugated linoleic acid on mammary carcinogenesis in animals.** *The American journal of clinical nutrition* 1997, **66:**1523S-1529S.
180. Belury MA: **Inhibition of carcinogenesis by conjugated linoleic acid: potential mechanisms of action.** *The Journal of nutrition* 2002, **132:**2995-2998.
181. Ip MM, Masso-Welch PA, Shoemaker SF, Shea-Eaton WK, Ip C: **Conjugated linoleic acid inhibits proliferation and induces apoptosis of normal rat mammary epithelial cells in primary culture.** *Experimental cell research* 1999, **250:**22-34.
182. Jiang WG, Hiscox S, Hallett MB, Horrobin DF, Mansel RE, Puntis MC: **Regulation of the expression of E-cadherin on human cancer cells by gamma-linolenic acid (GLA).** *Cancer research* 1995, **55:**5043-5048.
183. Simon JA, Chen YH, Bent S: **The relation of alpha-linolenic acid to the risk of prostate cancer: a systematic review and meta-analysis.** *The American journal of clinical nutrition* 2009, **89:**1558S-1564S.
184. Chavarro JE, Stampfer MJ, Li H, Campos H, Kurth T, Ma J: **A prospective study of polyunsaturated fatty acid levels in blood and prostate cancer risk.** *Cancer epidemiology, biomarkers & prevention : a publication of the American Association for Cancer Research, cosponsored by the American Society of Preventive Oncology* 2007, **16:**1364-1370.
185. Attar-Bashi NM, Frauman AG, Sinclair AJ: **Alpha-linolenic acid and the risk of prostate cancer. What is the evidence?** *The Journal of urology* 2004, **171:**1402-1407.

186. Hirayama A, Kami K, Sugimoto M, Sugawara M, Toki N, Onozuka H, Kinoshita T, Saito N, Ochiai A, Tomita M, et al: **Quantitative metabolome profiling of colon and stomach cancer microenvironment by capillary electrophoresis time-of-flight mass spectrometry.** *Cancer research* 2009, **69:**4918-4925.
187. Aa J, Yu L, Sun M, Liu L, Li M, Cao B, Shi J, Xu J, Cheng L, Zhou J, et al: **Metabolic features of the tumor microenvironment of gastric cancer and the link to the systemic macroenvironment.** *Metabolomics : Official journal of the Metabolomic Society* 2012, **8:**164-173.
188. Ma Y, Liu W, Peng J, Huang L, Zhang P, Zhao X, Cheng Y, Qin H: **A pilot study of gas chromatograph/mass spectrometry-based serum metabolic profiling of colorectal cancer after operation.** *Molecular biology reports* 2010, **37:**1403-1411.
189. Tuhackova Z, Gryga P, Helmich O, Dusek Z, Hradec J: **Enhanced utilization of 14-methylhexadecanoic acid for the synthesis of lipids during the growth of Walker 256 carcinoma in rats.** *Neoplasma* 1985, **32:**323-334.
190. Lee do Y, Bowen BP, Nguyen DH, Parsa S, Huang Y, Mao JH, Northen TR: **Low-dose ionizing radiation-induced blood plasma metabolic response in a diverse genetic mouse population.** *Radiation research* 2012, **178:**551-555.
191. Kemmner W, Kessel P, Sanchez-Ruderisch H, Moller H, Hinderlich S, Schlag PM, Detjen K: **Loss of UDP-N-acetylglucosamine 2-epimerase/N-acetylmannosamine kinase (GNE) induces apoptotic processes in pancreatic carcinoma cells.** *FASEB journal : official publication of the Federation of American Societies for Experimental Biology* 2012, **26:**938-946.
192. Ismail AM, In LL, Tasyriq M, Syamsir DR, Awang K, Omer Mustafa AH, Idris OF, Fadl-Elmula I, Hasima N: **Extra virgin olive oil potentiates the effects of aromatase inhibitors via glutathione depletion in estrogen receptor-positive human breast cancer (MCF-7) cells.** *Food and chemical toxicology : an international journal published for the British Industrial Biological Research Association* 2013, **62C:**817-824.
193. Hilvo M, Gade S, Hyotylainen T, Nekljudova V, Seppanen-Laakso T, Sysi-Aho M, Untch M, Huober J, von Minckwitz G, Denkert C, et al: **Monounsaturated fatty acids in serum triacylglycerols are associated with response to neoadjuvant chemotherapy in breast cancer patients.** *International journal of cancer Journal international du cancer* 2013.
194. Pucer A, Brglez V, Payre C, Punger Ar JE, Lambeau G, Petan T: **Group X secreted phospholipase A2 induces lipid droplet formation and prolongs breast cancer cell survival.** *Molecular cancer* 2013, **12:**111.
195. Abaffy T, Moller MG, Riemer DD, Milikowski C, Defazio RA: **Comparative analysis of volatile metabolomics signals from melanoma and benign skin: a pilot study.** *Metabolomics : Official journal of the Metabolomic Society* 2013, **9:**998-1008.
196. Louie SM, Roberts LS, Mulvihill MM, Luo K, Nomura DK: **Cancer cells incorporate and remodel exogenous palmitate into structural and oncogenic signaling lipids.** *Biochimica et biophysica acta* 2013, **1831:**1566-1572.
197. Bassett JK, Severi G, Hodge AM, MacInnis RJ, Gibson RA, Hopper JL, English DR, Giles GG: **Plasma phospholipid fatty acids, dietary fatty acids and prostate cancer risk.** *International journal of cancer Journal international du cancer* 2013, **133:**1882-1891.

198. Budhu A, Roessler S, Zhao X, Yu Z, Forgues M, Ji J, Karoly E, Qin LX, Ye QH, Jia HL, et al: **Integrated metabolite and gene expression profiles identify lipid biomarkers associated with progression of hepatocellular carcinoma and patient outcomes.** *Gastroenterology* 2013, **144:**1066-1075 e1061.
199. Denkert C, Budczies J, Weichert W, Wohlgemuth G, Scholz M, Kind T, Niesporek S, Noske A, Buckendahl A, Dietel M, Fiehn O: **Metabolite profiling of human colon carcinoma--deregulation of TCA cycle and amino acid turnover.** *Molecular cancer* 2008, **7:**72.
200. Ferreira AK, Meneguelo R, Pereira A, Filho OM, Chierice GO, Maria DA: **Synthetic phosphoethanolamine induces cell cycle arrest and apoptosis in human breast cancer MCF-7 cells through the mitochondrial pathway.** *Biomedicine & pharmacotherapy = Biomedecine & pharmacotherapie* 2013, **67:**481-487.
201. Ferreira AK, Freitas VM, Levy D, Ruiz JL, Bydlowski SP, Rici RE, Filho OM, Chierice GO, Maria DA: **Anti-angiogenic and anti-metastatic activity of synthetic phosphoethanolamine.** *PLoS One* 2013, **8:**e57937.
202. Ferreira AK, Meneguelo R, Pereira A, Mendonca Filho O, Chierice GO, Maria DA: **Anticancer effects of synthetic phosphoethanolamine on Ehrlich ascites tumor: an experimental study.** *Anticancer research* 2012, **32:**95-104.
203. Russo T, Colonna A, Salvatore F, Cimino F, Bridges S, Gurgo C: **Serum pseudouridine as a biochemical marker in the development of AKR mouse lymphoma.** *Cancer research* 1984, **44:**2567-2570.
204. Itoh K, Mizugaki M, Ishida N: **Detection of elevated amounts of urinary pseudouridine in cancer patients by use of a monoclonal antibody.** *Clinica chimica acta; international journal of clinical chemistry* 1989, **181:**305-315.
205. Possemato R, Marks KM, Shaul YD, Pacold ME, Kim D, Birsoy K, Sethumadhavan S, Woo HK, Jang HG, Jha AK, et al: **Functional genomics reveal that the serine synthesis pathway is essential in breast cancer.** *Nature* 2011, **476:**346-350.
206. Perroud B, Lee J, Valkova N, Dhirapong A, Lin PY, Fiehn O, Kultz D, Weiss RH: **Pathway analysis of kidney cancer using proteomics and metabolic profiling.** *Molecular cancer* 2006, **5:**64.
207. Bando T, Fujimura M, Kasahara K, Shibata K, Shirasaki H, Heki U, Iwasa K, Ueda A, Tomikawa S, Matsuda T: **Exposure to sorbitol induces resistance to cisplatin in human non-small-cell lung cancer cell lines.** *Anticancer research* 1997, **17:**3345-3348.
208. Cheng Y, Xie G, Chen T, Qiu Y, Zou X, Zheng M, Tan B, Feng B, Dong T, He P, et al: **Distinct urinary metabolic profile of human colorectal cancer.** *Journal of proteome research* 2012, **11:**1354-1363.
209. Yoshie T, Nishiumi S, Izumi Y, Sakai A, Inoue J, Azuma T, Yoshida M: **Regulation of the metabolite profile by an APC gene mutation in colorectal cancer.** *Cancer science* 2012, **103:**1010-1021.
210. El Agouza IM, Eissa SS, El Houseini MM, El-Nashar DE, Abd El Hameed OM: **Taurine: a novel tumor marker for enhanced detection of breast cancer among female patients.** *Angiogenesis* 2011, **14:**321-330.

211. Witherspoon M, Chen Q, Kopelovich L, Gross SS, Lipkin SM: **Unbiased metabolite profiling indicates that a diminished thymidine pool is the underlying mechanism of colon cancer chemoprevention by alpha-difluoromethylornithine.** *Cancer discovery* 2013, **3:**1072-1081.
212. Giatromanolaki A, Koukourakis MI, Koutsopoulos A, Mendrinos S, Sivridis E: **The metabolic interactions between tumor cells and tumor-associated stroma (TAS) in prostatic cancer.** *Cancer biology & therapy* 2012, **13:**1284-1289.
213. Rose DP: **The influence of sex, age and breast cancer on tryptophan metabolism.** *Clinica chimica acta; international journal of clinical chemistry* 1967, **18:**221-225.
214. Rose DP: **Tryptophan metabolism in carcinoma of the breast.** *Lancet* 1967, **1:**239-241.
215. Lyon DE, Walter JM, Starkweather AR, Schubert CM, McCain NL: **Tryptophan degradation in women with breast cancer: a pilot study.** *BMC research notes* 2011, **4:**156.
216. Juhasz C, Nahleh Z, Zitron I, Chugani DC, Janabi MZ, Bandyopadhyay S, Ali-Fehmi R, Mangner TJ, Chakraborty PK, Mittal S, Muzik O: **Tryptophan metabolism in breast cancers: molecular imaging and immunohistochemistry studies.** *Nuclear medicine and biology* 2012, **39:**926-932.
217. de Jong RA, Nijman HW, Boezen HM, Volmer M, Ten Hoor KA, Krijnen J, van der Zee AG, Hollema H, Kema IP: **Serum tryptophan and kynurenine concentrations as parameters for indoleamine 2,3-dioxygenase activity in patients with endometrial, ovarian, and vulvar cancer.** *International journal of gynecological cancer : official journal of the International Gynecological Cancer Society* 2011, **21:**1320-1327.
218. Suzuki Y, Suda T, Furuhashi K, Suzuki M, Fujie M, Hahimoto D, Nakamura Y, Inui N, Nakamura H, Chida K: **Increased serum kynurenine/tryptophan ratio correlates with disease progression in lung cancer.** *Lung cancer* 2010, **67:**361-365.
219. Schroecksnadel K, Winkler C, Fuith LC, Fuchs D: **Tryptophan degradation in patients with gynecological cancer correlates with immune activation.** *Cancer letters* 2005, **223:**323-329.
220. Zamanakou M, Germenis AE, Karanikas V: **Tumor immune escape mediated by indoleamine 2,3-dioxygenase.** *Immunology letters* 2007, **111:**69-75.
221. Sreekumar A, Poisson LM, Rajendiran TM, Khan AP, Cao Q, Yu J, Laxman B, Mehra R, Lonigro RJ, Li Y, et al: **Metabolomic profiles delineate potential role for sarcosine in prostate cancer progression.** *Nature* 2009, **457:**910-914.
222. Nam H, Chung BC, Kim Y, Lee K, Lee D: **Combining tissue transcriptomics and urine metabolomics for breast cancer biomarker identification.** *Bioinformatics* 2009, **25:**3151-3157.
223. Nishiumi S, Shinohara M, Ikeda A, Yoshie T, Hatano N, Kakuyama S, Mizuno S, Sanuki T, Kutsumi H, Fukusaki E, et al: **Serum metabolomics as a novel diagnostic approach for pancreatic cancer.** *Metabolomics : Official journal of the Metabolomic Society* 2010, **6:**518-528.
224. Zheng YF, Kong HW, Xiong JH, Lv S, Xu GW: **Clinical significance and prognostic value of urinary nucleosides in breast cancer patients.** *Clinical biochemistry* 2005, **38:**24-30.

225. Henneges C, Bullinger D, Fux R, Friese N, Seeger H, Neubauer H, Laufer S, Gleiter CH, Schwab M, Zell A, Kammerer B: **Prediction of breast cancer by profiling of urinary RNA metabolites using Support Vector Machine-based feature selection.** *BMC Cancer* 2009, **9:**104.
226. La S, Cho J, Kim J-H, Kim K-R: **Capillary electrophoretic profiling and pattern recognition analysis of urinary nucleosides from thyroid cancer patients.** *Analytica chimica acta* 2003, **486:**171-182.
